# Dynamic multi-omics and mechanistic modeling approach uncovers novel mechanisms of kidney fibrosis progression

**DOI:** 10.1038/s44320-025-00116-2

**Published:** 2025-06-05

**Authors:** Nadine Tuechler, Mira Lea Burtscher, Martin Garrido-Rodriguez, Muzamil Majid Khan, Dénes Türei, Christian Tischer, Sarah Kaspar, Jennifer Jasmin Schwarz, Frank Stein, Mandy Rettel, Rafael Kramann, Mikhail M Savitski, Julio Saez-Rodriguez, Rainer Pepperkok

**Affiliations:** 1 European Molecular Biology Laboratory (EMBL), Heidelberg, Germany; 2 Molecular Medicine Partnership Unit (MMPU), European Molecular Biology Laboratory and Heidelberg University, Heidelberg, Germany; 3 Heidelberg University, Faculty of Medicine, and Heidelberg University Hospital, Institute for Computational Biomedicine (ICB), Heidelberg, Germany; 4 Collaboration for Joint PhD Degree Between the European Molecular Biology Laboratory and Heidelberg University, Faculty of Biosciences, Heidelberg, Germany; 5 European Molecular Biology Laboratory, European Bioinformatics Institute (EMBL-EBI), Hinxton, Cambridgeshire, UK; 6 Translational Lung Research Center Heidelberg (TLRC), German Center for Lung Research (DZL), Heidelberg, Germany; 7 Department of Medicine 2 (Nephrology, Immunology, Rheumatology, Hypertension), RWTH Aachen University, Medical Faculty, Aachen, Germany; 8 Department of Internal Medicine, Nephrology and Transplantation, Erasmus Medical Center, Rotterdam, The Netherlands

**Keywords:** Kidney Fibrosis, Extracellular Matrix, Multi-omics, Time Points, Network Modeling, Chromatin, Transcription & Genomics, Urogenital System

## Abstract

Kidney fibrosis, characterized by excessive extracellular matrix deposition, is a progressive disease that, despite affecting 10% of the population, lacks specific treatments and suitable biomarkers. This study presents a comprehensive, time-resolved multi-omics analysis of kidney fibrosis using an in vitro model system based on human kidney PDGFRβ^+^ mesenchymal cells aimed at unraveling disease mechanisms. Using transcriptomics, proteomics, phosphoproteomics, and secretomics, we quantified over 14,000 biomolecules across seven time points following TGF-β stimulation. This revealed distinct temporal patterns in the expression and activity of known and potential kidney fibrosis markers and modulators. Data integration resulted in time-resolved multi-omic network models which allowed us to propose mechanisms related to fibrosis progression through early transcriptional reprogramming. Using siRNA knockdowns and phenotypic assays, we validated predictions and regulatory mechanisms underlying kidney fibrosis. In particular, we show that several early-activated transcription factors, including FLI1 and E2F1, act as negative regulators of collagen deposition and propose underlying molecular mechanisms. This work advances our understanding of the pathogenesis of kidney fibrosis and provides a resource to be further leveraged by the community.

## Synopsis

Multi-omics analysis of human kidney PDGFRβ^+^ mesenchymal cells quantified over 14,000 biomolecules across 8 time points, revealing temporal dynamics of fibrosis progression and identifying potentially novel regulatory mechanisms through integrated transcriptomic, proteomic, and secretomic profiling.

- Dynamic changes in molecular profiles were mapped through integrating 4 omics datasets, demonstrating distinct temporal patterns across 8 time points post TGF-β stimulation.
- Time-resolved multi-omics network analysis identified FLI1 and E2F1 as key early-stage negative regulators of TGF-β induced fibrotic mechanisms.
- The potential functionality of these early activated transcription factors was validated by siRNA knockdown experiments, suggesting that they play a yet uncharacterized role in TGF-β-driven fibrotic mechanisms.

## Abstract (web summary)

Multi-omics analysis of human kidney PDGFRβ^+^ mesenchymal cells quantified over 14,000 biomolecules across 8 time points, revealing temporal dynamics of fibrosis progression and identifying potentially novel regulatory mechanisms through integrated transcriptomic, proteomic, and secretomic profiling.

## Graphical abstract

**Figure:**
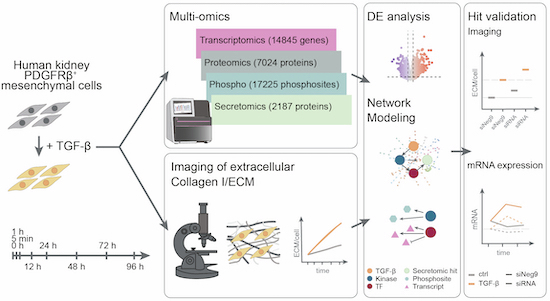

**Figure:**
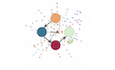

## Introduction

Kidney fibrosis is a characteristic component of chronic kidney disease (CKD), characterized by the progressive accumulation and remodeling of extracellular matrix (ECM) components (Yamashita and Kramann, 2024; Kim et al, 2022). This pathogenic process ultimately leads to disruption of tissue architecture and organ failure (Mullins et al, 2016; Kramann and Humphreys, 2014; Friedman et al, 2013). Despite the high prevalence and severity of CKD, there are no specific treatments available and the few existing biomarkers are either impractical or too expensive for routine use, such as CKD273 (Rodríguez-Ortiz et al, 2018; Verbeke et al, 2021; Argilés et al, 2013; Cañadas-Garre et al, 2018; Yamashita and Kramann, 2024; Prakash and Pinzani, 2017; Lousa et al, 2020; Yan et al, 2021; Rasmussen et al, 2019; Huang et al, 2023). This gap in clinical management of the disease underscores the urgent need for a deeper understanding of the underlying molecular mechanisms. Central to the pathogenesis of kidney fibrosis is the transdifferentiation of various cell types, including tissue-resident pericytes and fibroblasts, into myofibroblasts (Kendall and Feghali-Bostwick, 2014). Activated myofibroblasts serve as the primary source of ECM components, driving the fibrotic process (Kramann and Humphreys, 2014; Kuppe et al, 2021; Kim et al, 2022). Recent studies have shed light on cellular plasticity during fibrosis progression, but many aspects of the complexity of the disease remain poorly understood due to limitations such as single readouts, single time points, the inherent limitations of mouse models and the limited availability of human tissue samples (Prakash and Pinzani, 2017; Liang and Liu, 2023; Wei et al, 2024; Kuppe et al, 2021). Transcription factors (TFs) play a crucial role in myofibroblast differentiation and activation but remain challenging targets for drug development (Humphreys, 2018; Edeling et al, 2016; Zhang et al, 2018); (Yamashita and Kramann, 2024; Chang et al, 2012; Humphreys, 2018; Edeling et al, 2016; Zhang et al, 2018). This highlights the importance of exploring novel approaches to modulate TF activity as well as upstream signaling processes and downstream phenotypic effects in the context of fibrosis. Omics technologies combined with advanced computational methods offer powerful tools for studying complex processes like fibrosis progression (Lindenmeyer et al, 2021; Saez-Rodriguez et al, 2019; Niewczas et al, 2019). These include transcriptomics for tracking cellular differentiation, proteomics and phosphoproteomics for insights into signaling pathways, and secretomics for measuring related ECM and signaling components. Incorporating temporal resolution and integrating with phenotypic readouts are crucial for linking these molecular mechanisms to cellular and tissue-level processes. In this study, we use transforming growth factor beta (TGF-β) to induce fibrotic progression in PDGFRβ^+^ human kidney mesenchymal cells and investigate the underlying molecular mechanisms in detail. This addresses many of the limitations of existing approaches by providing (i) a perturbable system, (ii) a robust phenotypic readout of ECM deposition and collagen secretion, (iii) comprehensive multi-omics profiling of TGF-β-induced myofibroblast differentiation with high temporal resolution, and (iv) a mechanistic, multi-omics network-based integration approach to generate testable hypotheses.

By leveraging this in vitro model system and our integrative analysis approach, we uncovered new mechanistic insights into the pathogenesis of kidney fibrosis and identified potential biomarkers and therapeutic targets for this challenging disease.

## Results

### Phenotypic and molecular features defining fibrosis in PDGFRβ+ mesenchymal cells over time

**Figure 1:**
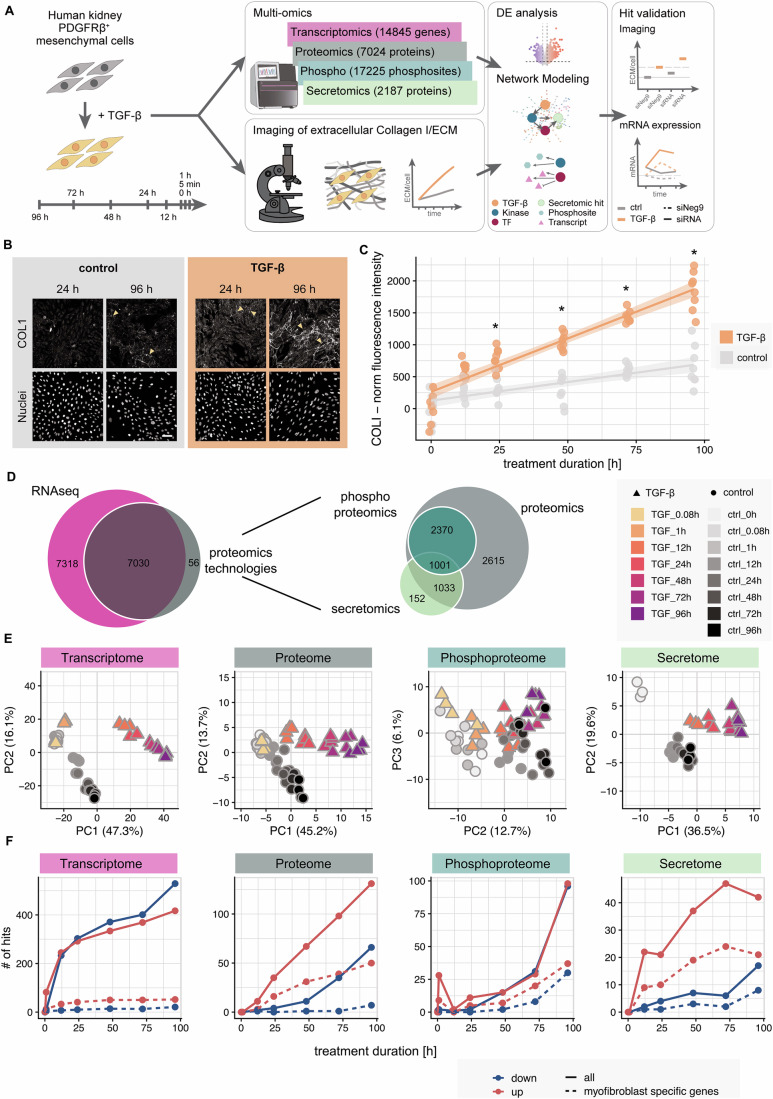
Phenotypic and molecular features defining fibrosis in PDGFRβ^+^ mesenchymal cells. (**A**) Overview of experimental and bioinformatic strategy. Human kidney PDGFRβ^+^ mesenchymal cells were treated with 10 ng/ml TGF-β for 5 min (0.08 h), 1, 12, 24, 48, 72, 96 h. Investigation of extracellular matrix changes over time and multi-omics data integration via mechanistic modeling. Factors of interest were further validated. (**B**) Immunofluorescence staining of extracellular COL1 and nuclei is shown at 96 h in control (macromolecular crowding + ascorbic acid) and TGF-β (TGF-β + macromolecular crowding + ascorbic acid) treated condition. Arrows highlight fibrillar collagen. Scale bar = 100 µm. (**C**) Extracellular COL1 fluorescence intensity over the specified time points and treatment conditions. Quantification of COL1 fluorescence staining was performed following background correction, correction for cell autofluorescence, and normalization to the number of nuclei. Single replicates are depicted by the dots (*n* = 36 images are averaged per replicate, *n* = 8 replicates per condition and time point). Data was square root transformed for statistical analysis using a linear mixed model (* corresponds to adjusted *P* value < 0.05, post hoc Sidak adjusted estimated marginal means comparisons test). (**D**) Venn diagrams representing the overlap of factors measured in the different data modalities after data processing filtering and normalization. (**E**) PCA scatter plots of PC1 and PC2 for the different omics modalities (top 10% most variable features). For phosphoproteomics, PC2 and PC3 are depicted. The gray color scale shows control samples over time, the yellow to violet gradient resolves the time for the TGF-β-treated samples. The shape of the points shows the control samples compared to the samples treated with TGF-β. Transcriptomics and secretomics samples were measured in triplicates, while for (phospho)proteomics four samples were measured. (**F**) Number of hits per modality and time point. Solid lines show the number of significantly deregulated transcripts (limma, absolute log2 fold change > log2(2) and BH-adjusted *P* value < 0.05, *n* = 14845) and proteins (limma, absolute log2 fold change > log2(1.5) and BH-adjusted *P* value < 0.05, group sizes are as in the plotting order *n* = 7024, *n* = 15186, *n* = 2187). Dashed lines show the number of significantly affected transcripts and proteins which overlap with genes specifically expressed in myofibroblasts of CKD patients (based on human PDGFRβ^+^ level 2 specificity scores by Kuppe et al, 2021).

In this study, we present a comprehensive investigation of kidney fibrosis using an in vitro model system that enables detailed phenotypic and molecular disease characterization and has previously been used in the context of kidney fibrosis research (Kuppe et al, 2021; Bouwens et al, 2025). The use of human PDGFRβ+ mesenchymal cells in this tissue culture setup enables the induction of fibrotic differentiation with accelerated collagen deposition and ECM remodeling after stimulation with TGF-β (Rønnow et al, 2020; Chen et al, 2009; Coentro et al, 2021; Khan et al, 2024). This system not only enables the time-resolved characterization of the phenotypic and molecular features of fibrotic processes, but also facilitates subsequent perturbation and validation (Fig. 1A).

Within 96 h of TGF-β stimulation, we observed a robust fibrosis-like phenotype as shown by COL1 secretion (Figs. 1B and EV1A,B), morphological changes (actin stress fiber formation, Fig. EV1C) and SMAD2/3 phosphorylation (Fig. EV2). Quantification of the extracellular COL1 fluorescence intensity revealed a significant increase of COL1 deposition upon TGF-β stimulation after 24 h compared to the corresponding control at 24 h, while we also observed increased collagen levels in the control conditions likely due to the macromolecular crowding agent and ascorbic acid used to accelerate collagen deposition (Fig. 1C). This highlights the accelerated time scale in which a fibrosis-like phenotype can be achieved with this setup. To gain insights into the underlying molecular mechanisms, we generated a comprehensive omics dataset spanning multiple time points after TGF-β stimulation. By combining mRNAseq with a multi-proteomics approach, more than 14,000 biomolecules could be reproducibly quantified in this system, including RNA moieties as well as intracellular, secreted, and phosphorylated proteins (Figs. 1D and EV3A). Compared to existing datasets that previously investigated TGF-β signaling or fibrosis, this multi-omics approach provides unprecedented depth and breadth of molecular insights with temporal resolution (Eddy et al, 2020; D’Souza et al, 2014; Arif et al, 2023; Lassé et al, 2023; Zhou et al, 2020a).

**Figure EV1:**

Time course of COL1 expression and cytoskeletal changes in response to TGF-β treatment. (**A**) Widefield microscopy images showing COL1 (top rows) and nuclear (Hoechst, bottom rows) staining in control and TGF-β-treated conditions at 0, 12, 24, 48, 72, and 96 h. (**B**) Quantification of COL1 expression per cell over time. Cells were cultured in four biological replicates (**A–C**) ± TGF-β for 0-96 h. Data points represent individual images, color-coded for control (gray) and TGF-β (orange) conditions. *Y* axis shows normalized COL1 intensity per cell, *x* axis shows nuclei count per image. Images with less than 20 nuclei were excluded from the analysis. (**C**) Sum Z-projections of confocal microscopy images displaying F-actin (phalloidin, top rows) and nuclear (bottom rows) staining in control and TGF-β treated conditions at the same time points as in (**A**). In panels (**A**, **C**), the control condition is shown in the lower half (gray background), while the TGF-β-treated condition is presented in the upper half (orange background). Scale bar = 100 µm. Yellow arrowheads indicate specific features of interest (fibrillar collagen or F-actin, respectively), while hollow arrowheads indicate autofluorescence of cells.

**Figure EV2:**

TGF-β-induced SMAD2 phosphorylation dynamics. (**A**) Western blot analysis of phosphorylated SMAD2 (p-SMAD2), total SMAD2, and α-tubulin (loading control) in response to TGF-β treatment over time. Time points range from 30 s to 96 h. siSMAD2 condition is included as a control. A spill-over has been labeled with ‘#’, the correct corresponding band and position has been labeled with ‘*’. (**B**) Western blot analysis of a biological replicate, similar to (**A**). Time points range from 2 min to 96 h. siSMAD2 and siNeg9 conditions are included as controls. (**C**) Quantification of p-SMAD2 levels normalized to total SMAD2 from (**A**, **B**). Each of the measurements was normalized to the corresponding loading control before calculating the log2 fold change between TGF-β and control-treated samples. The graph shows the log2 ratio of TGF-β/ctrl for p-SMAD2/SMAD2 across all time points. Two replicates (**A**, **B**) are represented. Source data are available online for this figure.

**Figure EV3:**

Reproducible multi-omic characterisation of TGF-β-induced effects over time. (**A**) Heatmap showing Pearson correlation coefficients between individual biological replicates, calculated using TMT reporter intensities (proteomics/phosphoproteomics) or gene counts (transcriptomic). Each row/column represents a single biological replicate. The color gradients indicate the time points for TGF-β-treated (yellow to purple) and control (greyscale) samples. (**B**) PCA scatter plot for phosphoproteomics data (PC1 vs PC2). Triangles represent TGF-β-treated samples, circles represent controls. Color gradients indicate time points as in (A). (**C**) Results of the differential expression analysis per time point and omics modality. Colors indicate transcripts and proteins significantly deregulated in abundance upon TGF-β stimulation in comparison to control samples (limma *t* test, adjusted *P* value < 0.05, absolute log2 fold change > log2(2) for transcripts, absolute log2 fold change > log2 (1.5) for proteins). Sample sizes are as in Fig. 1F. (**D**) Specificity score distribution for myofibroblasts from CKD patients (Human PDGFRβ+ level 2) retrieved from Kuppe et al, 2021 (Kuppe et al, 2021). The black line indicates the chosen specificity cutoff for comparisons to this study (0.2). (**E**) Heatmap showing Pearson correlations between time points and omics modalities for genes with a significantly deregulated transcript or protein in at least one modality (limma, adjusted *P* value < 0.05, absolute log2 fold change > log2(2) for transcripts, absolute log2 fold change > log2(1.5) for proteins, *n* = 242 and *n* = 780 for phosphopeitdes), filtered for genes detected in all modalities.

Stimulation of PDGFRβ+ mesenchymal cells with TGF-β leads to extensive perturbation of transcript and protein abundances as well as post-translational modifications (2435 molecules affected in at least one condition) that become more pronounced over time (Figs. 1E,F and EV3B,C; Dataset EV1). Comparing differentially expressed factors of the different time points and modalities shows good correlation at later time points, while differences at earlier time points suggest a shift in the timing of processes on the transcriptome and proteome level (Fig. EV3E). This is reinforced by the observation that transcriptome changes in the first phases of TGF-β stimulation exceed changes at the proteome level (Fig. 1F). This temporal resolution allows for a dynamic description of the fibrosis process, providing insights into the timing and progression of molecular events.

**Figure EV4:**
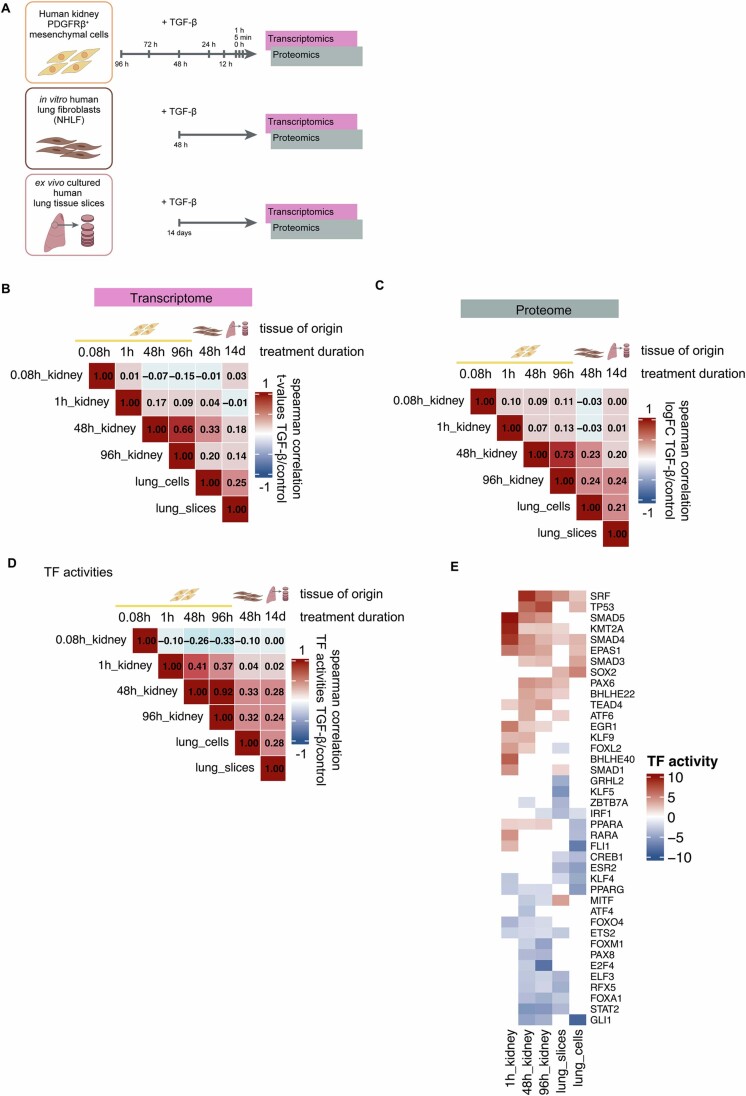
Cross-study Comparison of TGF-β Response in Cellular and Tissue Models. (**A**) Overview of different studies and datasets included into the comparison analysis. The transcriptomics and proteomics data generated in this study were compared to omics data from TGF-β-treated normal human lung fibroblasts (NHLF) (Khan et al, 2024) and human lung tissue slices (Khan et al, 2021). (**B**) Spearman correlation of all transcript t-values (DEseq2 or limma, *t*-test) comparing TGF-β treatment and the DMSO or untreated control for the samples of the different studies. Note: DMSO as control was only used in the lung-related studies. (**C**) Spearman correlation of all protein log2 fold-changes comparing TGF-β treatment and the DMSO control (limma, *t* test) for the samples of the different studies. (**D**) Spearman correlation of transcription factor activities affected upon TGF-β treatment or the samples of the different studies (enzyme activity enrichment analysis using decoupleR, normalized mean method, *P* value < 0.1 in at least one condition). (**E**) Top hits of transcription factor activities affected upon TGF-β treatment of the samples of the different studies (enzyme activity enrichment analysis using decoupleR, normalized mean method, *P* value < 0.1, top 10 TFs per sample).

To understand the extent to which these changes are comparable to fibrotic signaling in CKD patients, and thus to assess the translational relevance of the data presented, we compared our results with scRNAseq data from healthy and CKD patient donors (Kuppe et al, 2021). A considerable proportion (48% secretomics, 36% phosphoproteomics, 32% proteome, 1% transcriptomics) of the deregulated transcripts and proteins observed in this study belong to the set of myofibroblast-specific genes identified based on the cell type specificity scores of (Kuppe et al, 2021), (Figs. 1F and EV3D). Specifically, we observed the increase of myofibroblast-specific protein expression as the fibrotic process progresses, linking long-term patient data with in vitro data obtained over the course of hours. Together with the observed increased ECM accumulation, this indicates the activation of high-ECM-producing myofibroblasts (Kuppe et al, 2021) that are the main source of ECM in CKD. We further compared our findings with multi-omics data from two recent lung fibrosis studies (Khan et al, 2024, 2021) to determine cell line and organ specificity. These datasets included: (i) transcriptomics and proteomics from human lung fibroblasts treated with TGF-β for 48 h, and (ii) similar data from ex vivo cultured human lung tissue slices treated with TGF-β for 14 days (Fig. EV4A). We compared the results of the differential analysis for the transcriptomics and proteomics data for both datasets with two early and late time points of our human kidney cell dataset. We observed a correlation of the changes in the lung datasets with the late time points of TGF-β stimulation in the kidney cells to a similar extent as between the two lung datasets (Fig. EV4B,C). Interestingly, all early processes of the TGF-β response in kidney cells represented in the 5 min and 1 h time points appeared to be exclusive and absent in the lung data for both proteome and transcriptome (Fig. EV4B,C).

Taken together, our data provide a detailed coverage of the dynamic molecular and phenotypic changes occurring in PDGFRβ^+^ mesenchymal cells following TGF-β stimulation.

### Functional characterization of signaling and transcription processes in kidney fibrosis

**Figure EV5:**
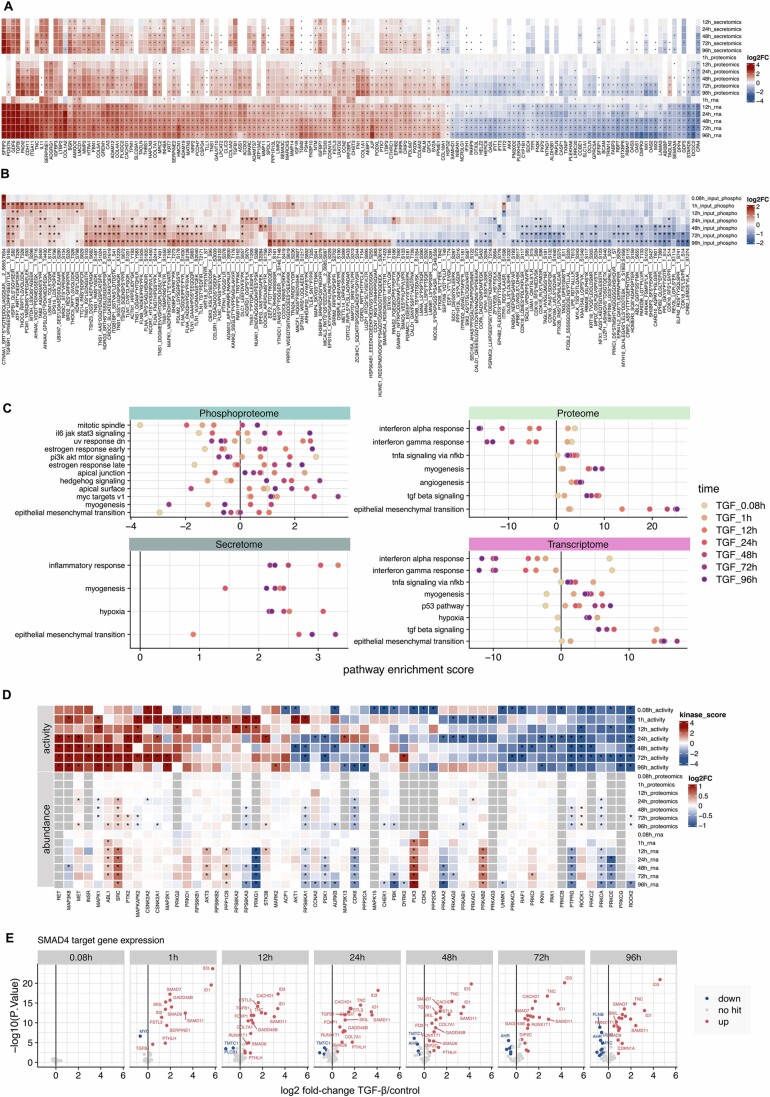
Temporal multi-omics analysis of TGF-β signaling dynamics. (**A**) Heatmap of differential abundance per time point of transcripts, proteins and secreted proteins across time points. Included are genes significantly affected in at least two modalities upon TGF-β stimulation (limma, *t*-test, adjusted *P* value < 0.05, absolute log2 fold change > log2(2) for transcripts, absolute log2 fold change > log2(1.5) for proteins). The color intensity indicates the magnitude and direction of change. Exact *P* values are listed in Dataset EV1. (**B**) Heatmap displaying differential abundance per time point of the top affected phosphopeptides upon TGF-β stimulation. The color indicates the direction of change. Exact *P* values are listed in Dataset EV1. (**C**) Dot plots of top significantly enriched pathways (GSEA using decoupleR with MSIGDB reactome database, *P* value < 0.05) per time point and omics modality. Color represents the time points. (**D**) Heatmap of differential protein and transcript abundance with matched activity scores of kinases/phosphatases per time point. Kinases/phosphatases were considered if they showed significantly altered activity upon TGF-β stimulation (enzyme activity enrichment analysis using decoupleR, normalized weighted mean test, *P* value < 0.05 and absolute enrichment score > 3). Exact *P* values are listed in Dataset EV1. (**E**) Exemplary target profile of the transcription factor SMAD4 over time. Each point represents a known SMAD4 target transcript, with color indicating up- or downregulation. The *y* axis shows statistical significance (*n* = 68, limma’s *t* test, BH-adjusted *P* value < 0.05 and absolute log2 fold change > log2(2)). The differential abundance signal of these known target transcripts is summarized to an activity score per time point.

The induced molecular phenotype and its dynamic nature become particularly evident when studying specific examples over time and across different omics layers (Fig. EV5A,B).

**Figure 2:**
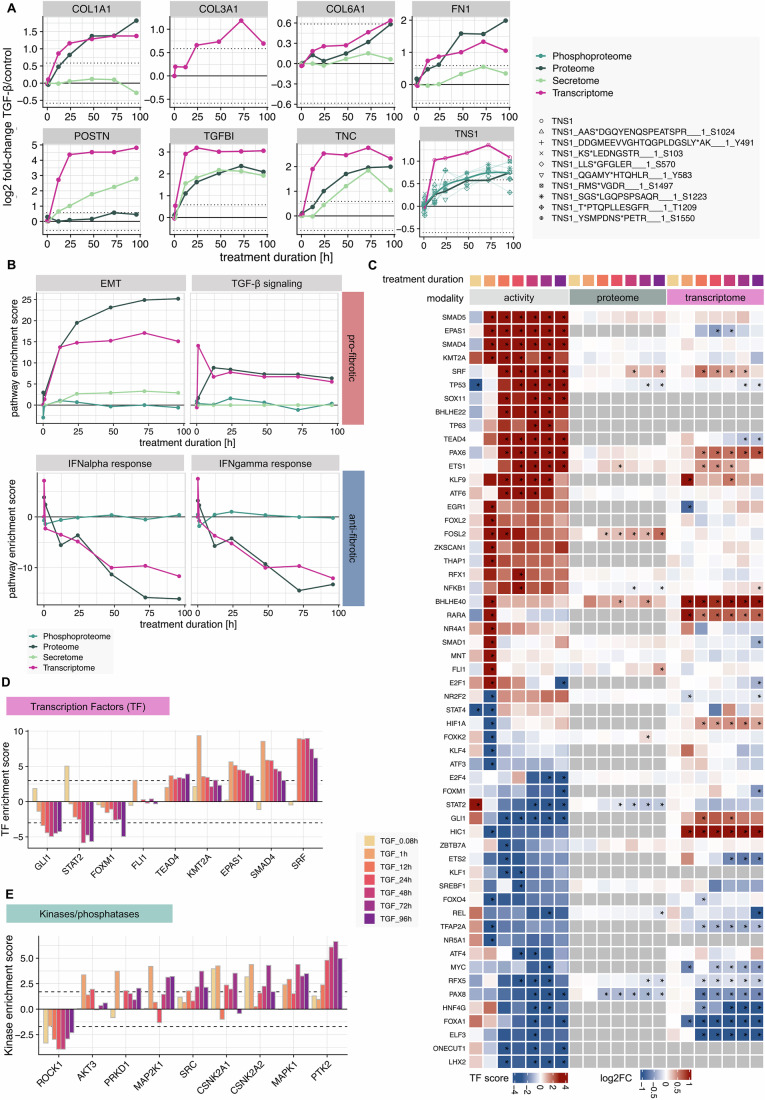
Functional characterization of signaling and transcription processes in kidney fibrosis. (**A**) Differential abundance of known fibrotic markers upon TGF-β stimulation per time point in the different modalities, coded by color. (**B**) Selected significantly enriched pathways (GSEA using decoupleR with MSIGDB reactome database, *P* value < 0.05) per time point and modality, coded by color. (**C**) Heatmap of differential protein and transcript abundance and matched activity scores of transcription factors per time point. Transcription factors were considered if they showed significantly altered activity upon TGF-β stimulation (enzyme activity enrichment analysis using decoupleR, *P* value < 0.05 and absolute enrichment score > 3). Significance on the proteome and transcriptome level was estimated using limma’s *t* test (BH-adjusted *P* value < 0.05 and absolute log2 fold change > log2(2) for transcripts and > log2(1.5) for proteins). Exact *P* values are listed in Dataset EV1 and EV3. (**D**) Transcription factor and (**E**) kinase/phosphatase enrichment scores per time point for selected significantly affected examples (enzyme activity enrichment analysis using decoupleR, *P* value < 0.05 and absolute enrichment score > 3 for transcription factors or absolute enrichment score > 2 for kinases/phosphatases).

Our data shows a strong upregulation of known fibrosis readouts, but with distinct temporal dynamics (Fig. 2A). One example is fibronectin (FN1), a glycoprotein involved in collagen assembly and proposed drug target for fibrotic diseases (Moita et al, 2022), that shows strong upregulation over time in both transcriptome and proteome. Periostin (POSTN), a protein involved in cross-linking of the ECM, differentiation, and kidney fibrosis (François and Chatziantoniou, 2018; Schnieder et al, 2020), is immediately secreted upon stimulation. In contrast, Tenascin (TNC) maintains consistently high levels. TNC, an extracellular matrix glycoprotein produced by fibroblasts, promotes their activation and proliferation in an autocrine fashion (Moita et al, 2022; Huang et al, 2023). Tensin-1 (TNS1), a protein involved in integrin signaling, myofibroblast differentiation, and ECM deposition, is affected in expression as well as heavily phosphorylated which has not been reported before as a regulation mechanism in fibrosis (Fig. 2A, (Huang and Lo, 2023; Bernau et al, 2017). These variations underscore the differential temporal regulation of fibrotic markers in our experimental cell system.

Similarly, pathways known to exert a profibrotic effect, such as EMT and TGF-β, are upregulated while antifibrotic pathways like interferon signaling are downregulated with distinct temporal dynamics (Figs. 2B and EV5C; Dataset EV2).

Illustrating these time-dependent changes, our data on COL1A1 mRNA expression support and extend the findings from the imaging assay (Fig. 1B,C and EV1A,B). We observed an increase in COL1A1 mRNA and protein levels upon TGF-β stimulation over time, contrasting with a decrease in its abundance in the secretomics data. This pattern aligns with the fact that COL1A1 is incorporated into the insoluble fraction of the ECM, as suggested by previous studies (Naba et al, 2016; Hynes and Naba, 2012). Furthermore, these results highlight the role of COL1 as an important regulator in the positive feedback mechanisms driving fibrosis such as influencing the matrix stiffness, demonstrating how individual components contribute to the complex, time-dependent nature of the fibrotic response observed at the pathway level (Kim et al, 2022; Agarwal et al, 2020; Devos et al, 2023; Zhou et al, 2020b).

We next inferred the activity of TFs and kinases from differential transcript and phosphoprotein abundance information, respectively, using decoupleR (Badia-I-Mompel et al, 2022). 128 transcription factors (Fig. 2C,D; Dataset EV3) and 70 kinases/phosphatases (Figs. 2E and EV5D; Dataset EV3) show significantly altered activity upon TGF-β stimulation with different temporal dynamics. The top hits include important drivers of TGF-β signaling such as members of the SMAD family (Fig. 2C,D). The underlying transcriptional changes of this activity prediction can be investigated by subsetting the differential expression information for known targets of a TF of interest, e.g., for SMAD4 (Fig. EV5E). Other interesting factors include KMT2A encoding MLL1 which has a reported role in renal fibrogenesis (Jin et al, 2022) as well as TEAD4 and SRF coactivators in YAP-TAZ signaling which leads to the production of ECM proteins in a mechanosensitive manner (Bernau et al, 2017; Hinz and Lagares, 2020; Kim et al, 2022). The consequences of JAK-STAT signaling, such as the activity of the transcription factors STAT2 and GLI1, can also be seen in this analysis, with a peak in activity after 15 min, followed by decreased activity but increased expression in the case of GLI1, indicating a complicated, time-dependent transcriptional regulation (Türei et al, 2021, 2016). While most of these transcription factors show a constitutive up/downregulation over time, there are examples such as FLI1 with a temporally regulated activity that only increases after one hour of stimulation (Fig. 2D). Note that this analysis allows assessment of TF activity, which can be difficult with direct measurement, as we for example were unable to detect activating SMAD2/3 phosphorylation in the phosphoproteome experiment, due to limited coverage of low-abundance proteins.

In addition, we performed a similar analysis for kinases and phosphatases based on the obtained phosphoproteomic data (Figs. 2E and EV5E). While the canonical response to TGF-β stimulation via SMADs is well reflected in the transcription factor activity estimation results, noncanonical fibrotic signaling pathways such as MAPK (MAPK1, MAP2K1, PTK2), RHO-ROCK (ROCK, PTK2), and PI3K-AKT (AKT3) become evident in this analysis (Figs. 2E and EV5E) (Park and Yoo, 2022). In addition, the analysis suggests that TGF-β stimulation activates less well-characterized pathways via casein kinase 2 (CSNK2A1, CSNK2A2), which could be an option for further investigation and characterization (Borgo et al, 2021).

To better understand the robustness and generalizability of the obtained results, an additional estimation of TF activity was performed using two previously introduced lung fibrosis datasets (Khan et al, 2024, 2021) (Fig. EV4). Similar to the results of the differential analysis (Fig. EV4B,C), the predicted TF activities after TGF-β stimulation in lung cells and tissue slices showed a correlation with late time points of kidney cell stimulation, while all early activation processes could not be detected with the later readout times (Fig. EV4D). Of note, several of the TFs correlating between kidney and lung cells are known canonical TGF-β mediators (for example, SMAD3/4, SRF, and GLI1; Fig. EV4E). The multi-omics analysis of TGF-β-induced kidney fibrosis revealed complex temporal dynamics in the expression and activity of fibrosis markers, signaling pathways, transcription factors, and kinases/phosphatases, demonstrating both canonical and noncanonical responses while uncovering potential novel regulatory mechanisms and therapeutic targets for further investigation.

### Developing a time-resolved multi-omic mechanistic model of kidney fibrosis signaling

We next integrated the findings obtained from the differential expression and kinase and TF activity analyzes in a network model, using a modified version of COSMOS, an optimization method that identifies putative causal paths explaining changes in enzymes with altered activity and multi-omics measurements based on a causal Prior Knowledge Network (PKN) (Dugourd et al, 2021).

**Figure 3:**
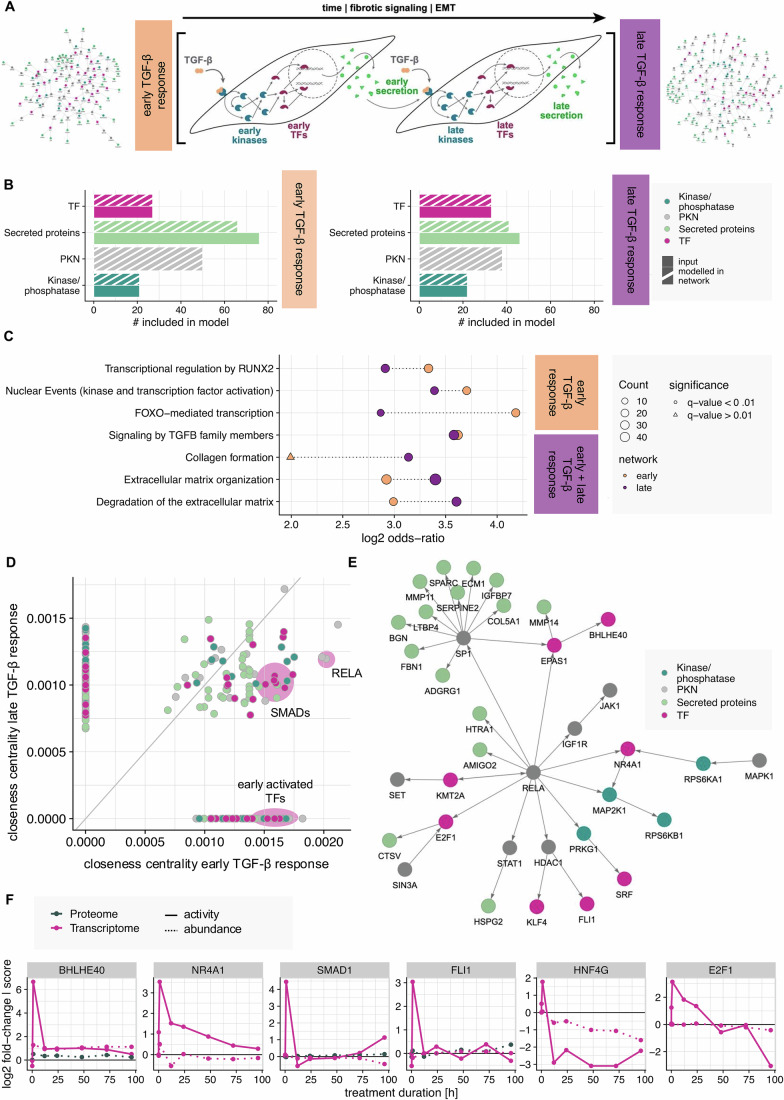
Developing a time-resolved multi-omic mechanistic model of kidney fibrosis signaling. (**A**) Schematic representation of the chosen computational integration strategy. Two network models were created to reflect the early (network on the left) and late (right network) response to TGF-β stimulation, integrating transcriptomics, phosphoproteomics, and secretomics data for different time points. By considering early secreted factors as upstream stimuli for the late response network, autocrine signaling can be modeled. To generate the network models, the COSMOS method was used with a PKN consisting of signed directed protein-protein interactions retrieved from Omnipath. (**B**) Properties of the resulting network models. For both models, all input enzymes (TFs and kinases/phosphatases) as well as the majority of considered secreted proteins could be integrated into the solution. (**C**) Selected significantly overrepresented pathways in the early and late network models (pathway overrepresentation analysis of network nodes using the Reactome pathway database, *P* value < 0.05). Point color indicates the network for which the enrichment has been calculated. Point size shows the number of nodes in the network mapped to the pathway and point shape the significance (*q* value). (**D**) Comparison of closeness centrality measures per node for the early and late TGF-β response network model. Points are colored by node type. (**E**) RELA-related signaling in the early TGF-β response network model. Node color indicates node type. (**F**) Temporal activity and abundance profiles for selected early activated TFs. The color indicates the underlying modality, the line type, and the data type.

To capture the temporal dimension of the observed fibrotic response, we created network models for both early and late stages, built from the corresponding subsets of the multi-omics data (detailed description in “Network modeling”). We focused on the obtained secretomics data to account for autocrine signaling over time by using early secreted factors as upstream input for the late network model. This provides a mechanistic molecular hypothesis for the observed ECM deposition at later time points, thus reflecting the dynamic nature of cellular communication (Fig. 3A). As underlying PKN, we used a directed and signed protein-protein interaction network retrieved from Omnipath (Türei et al, 2016, 2021). It should be noted that this modeling approach provides molecular associations that are not guided by commonly assumed pathway topologies and cannot be considered as a visualization of canonical pathways. This is in line with recent findings showing that the definition of so-called canonical pathways can be biased by the way biochemical research is done and is not necessarily useful to reflect signaling processes (Garrido-Rodriguez et al, 2024).

**Figure EV6:**
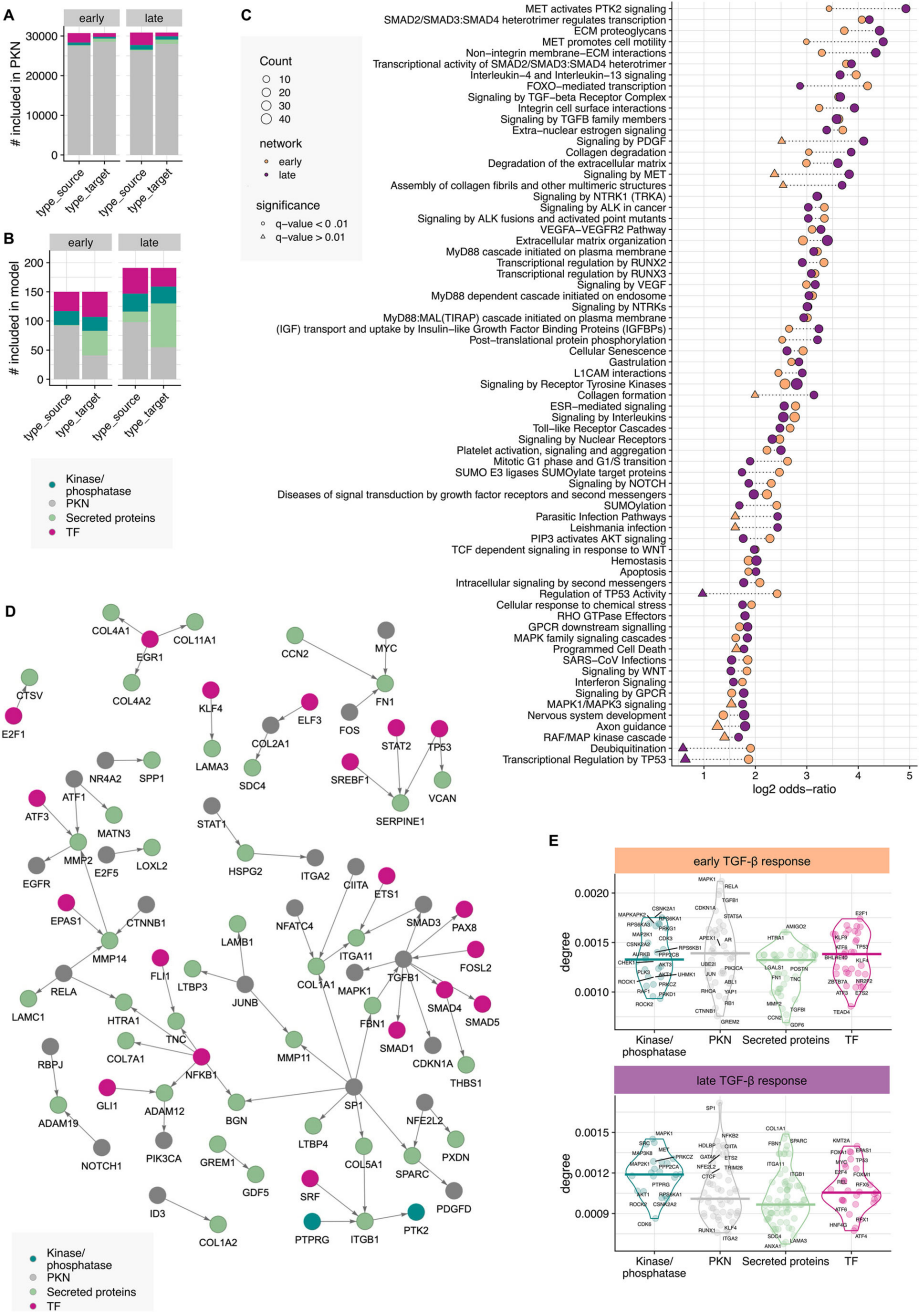
Network structure and analysis of early and late TGF-β response models. (**A**) Overview of node types in the prior knowledge network. All modalities are reflected as protein-protein interaction source and target for the early and late TGF-β response model. (**B**) Overview of node types in the obtained solution network models for early and late TGF-β response. The node type for protein-protein interaction source and target nodes reflect the chosen hierarchy. (**C**) Significant pathways in early and late TGF-β response network model (pathway overrepresentation analysis, unpaired two-sided Wilcoxon test, of network nodes using the Reactome pathway database, *P* value < 0.05). Point color indicates the network for which the enrichment has been calculated. Point size shows the number of nodes in the network mapped to the pathway. (**D**) Reactome TGF-β signaling pathway in network models shows links between different node types. Node color indicates node type. (**E**) Node centrality in the early and late network model per node type (*n* in the order of the plot, early: 21, 36, 40, 33; late: 21, 50, 66, 27).

Besides proteins with altered secretion upon TGF-β stimulation, we selected the observed early and late deregulated TFs and kinases/phosphatases as input nodes and confirmed that all moieties are represented in the PKN (Fig. EV6A). The integration process incorporated all input enzymes and measurements, as well as most secreted proteins used as model input, while maintaining a manageable solution size (Figs. 3B and EV6B; Dataset EV4; Table EV1).

The resulting networks reflect the expected TGF-β response, as shown by an overrepresentation analysis of the network nodes (Figs. 3C and EV6C). TGF-β signaling and ECM remodeling processes are among the most enriched pathways in both models, while a series of effects on transcriptional regulation seem to be strengthened in the early network. The signature of TGF-β signaling in the networks is extensive, linking several TFs, kinases, and secreted proteins, highlighting the value of integrating different modalities (Fig. EV6D).

Analyzing node connectivity based on their closeness centrality in the network revealed that transcription factors and kinases are more connected than secreted proteins (Figs. 3D and EV6E), with, for example, SMAD TFs remaining critical in both early and late networks. While this pattern may derive from prior knowledge, a number of network-exclusive nodes as well as nodes with different closeness centrality between the two networks illustrate shifts in the connectivity of the nodes and thus potentially biological relevance over time (Fig. 3D).

As mentioned before, network node enrichment analyses underscored the significance of early transcriptional events, while the analysis of node centrality underscores the importance of the TF RELA in the early network (Fig. 3C–E). RELA is being studied in the context of fibrotic disease (Moles et al, 2013; Chung et al, 2017) and is connected to many early-activated TFs in the network, some well-documented in fibrosis (Kendall and Feghali-Bostwick, 2014; Mikhailova et al, 2023; Kum et al, 2007; Goffin et al, 2010).

A group of TFs governed by RELA showed a similar activity pattern characterized by an early activation peak (Fig. 3E,F). We focused on a number of these early-acting TFs that have been poorly or not at all characterized in the context of fibrosis, but were predicted to have a regulating role on differential mRNA expression. Those include Transcription Factor E2F1, Friend leukemia integration 1 transcription factor (FLI1), Nuclear Receptor Subfamily 4 Group A Member 1 (NR4A1), and Class E basic helix-loop-helix protein 40 (BHLHE40) for validation, which are all predicted to be downstream of RELA. Similar profiles, with an upregulated activity at 1 h post TGF-β stimulation were shown by the TFs Mothers against decapentaplegic homolog 1 (SMAD1), and Hepatocyte nuclear factor 4-gamma (HNF4G).

To summarize, the integration of multi-omic data into time-resolved network models of early and late fibrotic in vitro processes revealed dynamic shifts in signaling pathways, transcription factor activities, and protein interactions, highlighting the temporal complexity of ECM production in kidney fibroblasts upon TGF-β stimulation and identifying both well-known and novel regulatory factors for further investigation.

### Experimental validation confirms the implication of selected TFs in regulating ECM deposition

**Figure 4:**
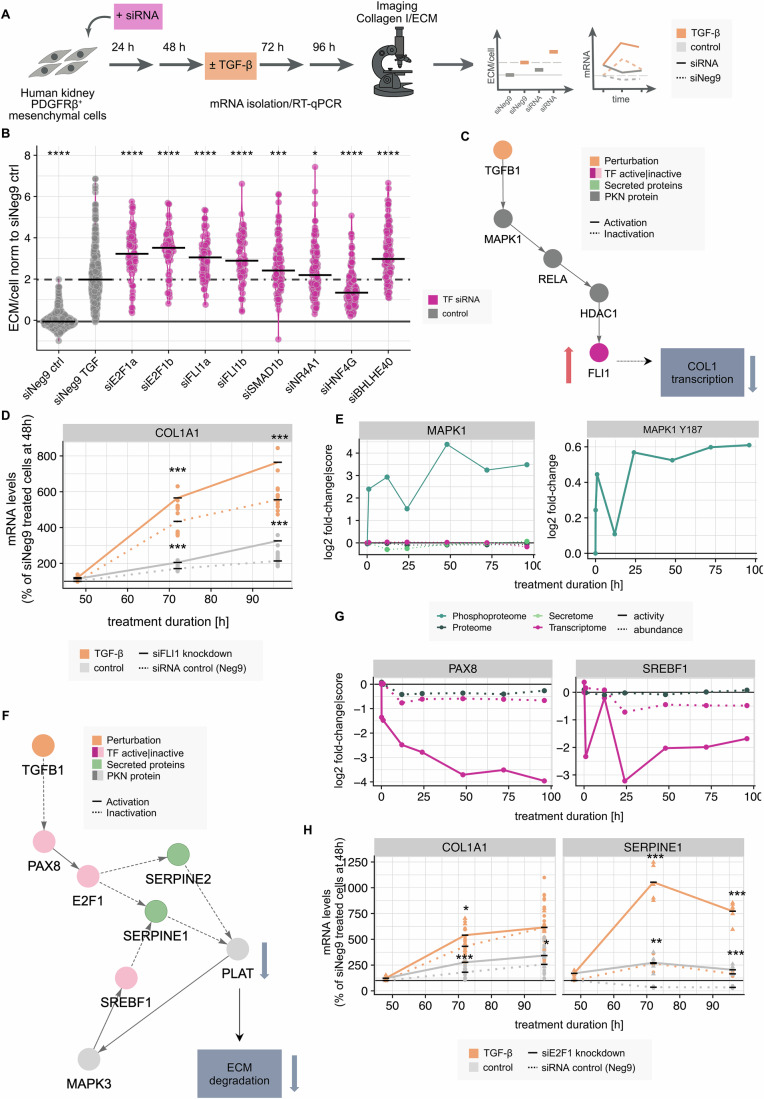
Validation of predicted fibrosis modulators and related molecular mechanisms. (**A**) Schematic representation of the experimental workflow for validation experiments. (**B**) Fluorescence intensity of ECM for selected TF knockdowns (purple, 48 h knockdown followed by additional 48 h of concurrent knockdown and TGF-β treatment) and the siNeg9 TGF-β treated/unstimulated controls (gray). Intensities of each knockdown were compared to the siNeg9 TGF-β treated condition (siNeg9 TGF) using a unpaired two-sided *t* test (*n* = 2–3 biological replicates, each with ~ 36 images, * corresponds to *P* value < 0.05, ** corresponds to *P* value < 0.01, *** corresponds to *P* value < 0.001, exact *P* values as they appear in the plot: 2.2e-16, 2e-10, 4.1e-11, 2.3e-10, 8.5e-9, 4.4e-3, 0.023, 1e-6, 1.7e-13). Black bars represent the median fluorescence intensity per distribution. (**C**) Subnetwork of early TGF-β response network model showing directed protein-protein interactions leading to FLI1 activation. Node color indicates node type, edge line type indicates activation/inhibition. (**D**) RT-qPCR data to confirm FLI1 knockdown effect on its potential downstream target COL1A1 + /-TGF-β stimulation at different time points. Color indicates TGF-β stimulation vs control, line type indicates siNeg9 control vs TF knockdown. Significance has been tested per time point and treatment condition using a two-sided unpaired *t* test (*n* = 3 biological replicates, * corresponds to *P* value < 0.05, ** corresponds to *P* value < 0.01, *** corresponds to *P* value < 0.001, exact *P* values as they appear in the plot: 0.0000822, 0.00000746, 0.00000106, 0.0000000246). (**E**) Temporal activity and abundance profiles for the MAPK1 kinase and the MAPK1 Y187 phosphorylation site. The color indicates the underlying modality, the line type shows the data type. (**F**) Subnetwork of late TGF-β response network model showing directed protein-protein interactions leading to E2F1 inactivation upstream of SERPINE1 activation. Node color indicates node type, and edge line type indicates activation/inhibition. (**G**) Temporal activity and abundance profiles for the PAX8 and SREBF1 transcription factors. The color indicates the underlying modality, and the line type shows the data type. (**H**) RT-qPCR data to confirm the effect of E2F1 knockdown on its potential downstream targets COL1A1 and SERPINE1 + /-TGF-β stimulation at different time points. Color indicates TGF-β stimulation vs control, line type indicates siNeg9 control vs TF knockdown. Significance has been tested per time point and treatment condition using a two-sided unpaired *t* test (*n* = 3 biological replicates, * corresponds to *P* value < 0.05, ** corresponds to *P* value < 0.01, *** corresponds to *P* value < 0.001, exact *P* values as they appear in the plot: 0.0110, 0.000662, 0.0173, 0.000191, 0.0000104, 0.00238, 0.0000758).

To further validate the role of these transcription factors in the observed ECM accumulation, we exploited the perturbability of the in vitro model system. We investigated their role in ECM remodeling and collagen deposition using siRNA knockdowns and subsequent imaging of the deposited ECM. By monitoring ECM production, we took advantage of a direct phenotypic readout of the knockdown performed, while RT-qPCR experiments allowed us to track the effects of TF knockdowns on the mRNA expression of their targets at the molecular level (Fig. 4A; Dataset EV5).

**Figure EV7:**
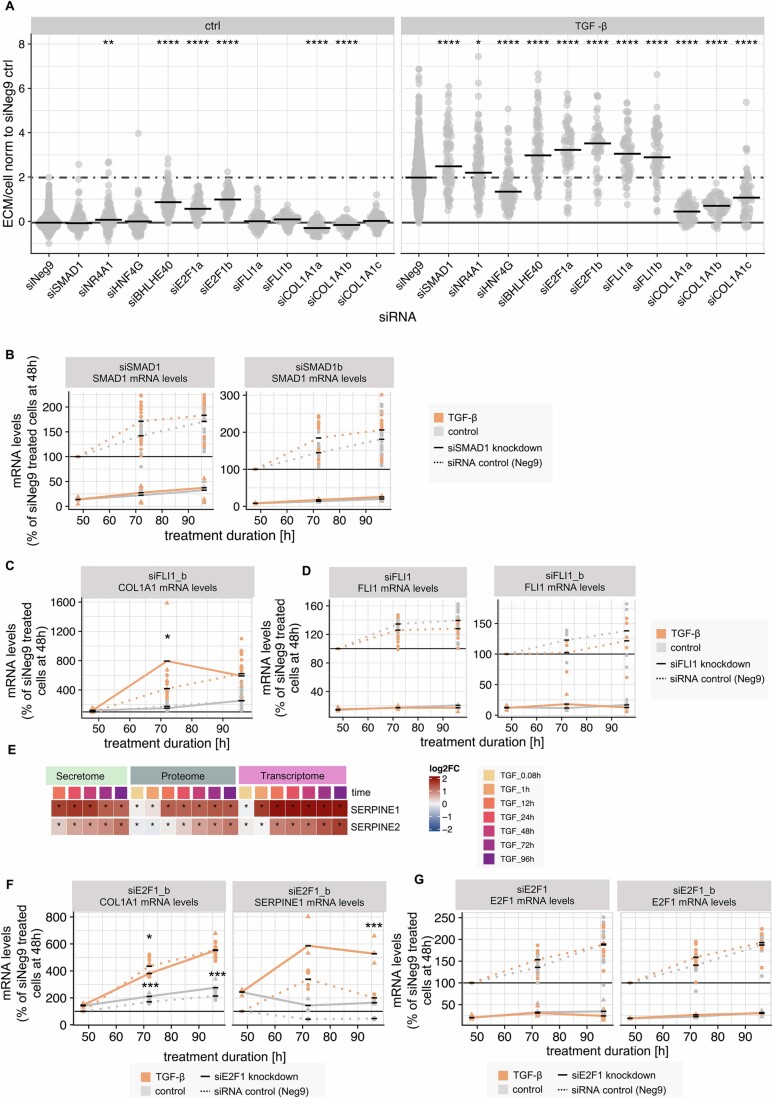
Functional validation of key transcription factors regulating ECM deposition and fibrotic gene expression. (**A**) Fluorescence intensity of ECM for both untreated (ctrl) and TGF-β stimulated conditions for all performed knockdowns (96 h knockdown followed by 48 h +/- TGF-β treatment). Intensities of each knockdown were compared to the siNeg9 control condition (+/- TGF-β treatment) using a two-sided unpaired *t* test (*n* = 2–3 biological replicates, each with ~ 36 images, * corresponds to *P* value < 0.05, ** corresponds to *P* value < 0.01, *** corresponds to *P* value < 0.001, exact *P* values as they appear in the plot: 0.46, 0.0073, 0.67, 2.2e-16, 2.2e-16, 2.2e1-6, 0.32, 0.05, 2.2e-6, 0.42, 8.7e-6, 0.023, 1e-6, 1.7e-13, 2e-10, 4.1e-11, 2.3e-10, 8.5e-9, 2.2e-16, 2.2e-16, 1.2e-9). For panels B-D and F, color indicates TGF-β stimulation (orange) vs control (gray). Line type distinguishes between siNeg9 control (solid) and target gene knockdown (dashed). (**B**) RT-qPCR data showing SMAD1 knockdown efficiency for various mRNAs across different time points (*n* = 3–5 biological replicates). (**C**) RT-qPCR results demonstrating the effect of FLI1 knockdown (using a second set of siRNA) on its potential downstream target COL1A1 at different time points. Significance has been tested per time point and treatment condition using a two-sided unpaired *t* test (*n* = 4 biological replicates, * corresponds to *P* value < 0.05, ** corresponds to *P* value < 0.01, *** corresponds to *P* value < 0.001, exact *P* values as they appear in the plot: 0.0128). (**D**) RT-qPCR data confirming FLI1 knockdown efficiency for multiple mRNAs at various time points (*n* = 3–4 biological replicates). (**E**) Differential expression analysis results for SERPINE1 and SERPINE2 in the secretomics, proteomics, and transcriptomics data per time point. Significance is indicated by one star (limma *t* test, adjusted *P* value < 0.05, absolute log2 fold change > log2(1.5) or absolute log2 fold change > log2(2) for transcriptomics data, Exact *P* values are listed in Dataset EV1). (**F**) RT-qPCR data to confirm E2F1 knockdown effect on its potential downstream targets COL1A1 and SERPINE1 + /-TGF-β stimulation at different time points using a second set of siRNA.Significance has been tested per time point and treatment condition using a two-sided unpaired *t* test (*n* = 4 biological replicates, * corresponds to *P* value < 0.05, ** corresponds to *P* value < 0.01, *** corresponds to *P* value < 0.001, exact *P* values as they appear in the plot: 0.03, 0.000239, 0.00000151, 0.000298, 0.00676, 0.000000510). (**G**) RT-qPCR data to confirm E2F1 knockdown efficiency for different mRNAs (columns) +/-TGF-β stimulation at different time points (*n* = 3–4 biological replicates).

Analysis of the fluorescence intensity of the deposited ECM after knockdown of the early activated TFs revealed a general trend towards increased deposition compared to a siNeg9 control (Figs. 4B and EV7A). This effect is considerably pronounced upon TGF-β stimulation, indicating the importance of these TFs as potential negative regulators of collagen secretion, given fibrotic signaling (Figs. 4B and EV7A, right panel). All six TF knockdowns had a significant impact on ECM deposition (unpaired two-sided *t* test, *P* value < 0.05), but for NR4A1 the effect magnitude was quite small, while the HNF4G knockdown was the only example that showed an opposite effect. Notably, the activity of HNF4G was strongly downregulated after 12 h of TGF-β stimulation, which is not the case for the other TFs we considered for validation (Fig. 3F). The collagen knockdowns, performed as a positive control, led to the expected reduction in collagen deposition (Fig. EV7A). Following knockdown of SMAD1, ECM deposition also increased (Fig. 4A), which was confirmed with a second siRNA (Fig. EV7A,B).

By combining insights into potential mechanisms of TF activity from our multi-omics models with information on the expression of target genes upon knockdown, we can investigate the role of specific TFs in detail. FLI1 is involved in early signaling around RELA, with an activity peak at 1 h and no deregulation of activity or abundance thereafter (Fig. 3E,F). Further investigation of the network reveals a possible regulation via MAPK1-RELA-HDAC, which is downstream of TGFB1 and leads to the activation of FLI1 (Fig. 4C). Consistent with the imaging data, COL1A1 mRNA production is also increased upon FLI1 knockdown at the mRNA level (Figs. 4D and EV7C,D; Dataset EV6). These observations align with existing literature describing the role of FLI1 as an inhibitor of collagen production controlled by HDAC1 (Fig. 4C) (Mikhailova et al, 2023; Wang et al, 2006). Regulation by MAPK1 is further supported by the predicted increased activity of the kinase at an early time point, as well as significantly increased phosphorylation in the activation loop of the kinase (Y187, limma, adjusted *P* value < 0.05, absolute log2 fold change > log2(1.5)), which has been reported to lead to its increased activity (Fig. 4E, Schmidlin et al, 2019). These findings highlight the benefits of the integrative approach, as they would not have been detected by a single omics modality.

A second very interesting case is the transcription factor E2F1, which shows increased activity at earlier time points and then appears to be deactivated over time (Fig. 3F). This is also in line with earlier studies using these cells that show that E2F1 activity is enhanced at 24 h but decreased at 72 h post TGF-β treatment (Bouwens et al, 2025). Upon E2F1 knockdown, increased deposition of ECM is observed in the imaging data, as with most other early-activated TFs (Fig. 4B).

In the early network model, E2F1 activation is predicted to occur downstream of RELA. (Fig. 3E). The temporal downregulation of E2F1 is included in the late network model downstream of TGFB1 and PAX8 (Fig. 4F), consistent with the estimated PAX8 activity, which shows a strong downregulation over time (Fig. 4G).

Moreover, E2F1 regulation is associated with secretory processes, as shown by the late network model, which suggests that SERPINE1 (also known as plasminogen activator inhibitor-1 PAI-1) and SERPINE2 secretion, key modulators of collagen deposition, are upregulated downstream of E2F1 inhibition (Figs. 4F and EV7E). SERPINE1 and SERPINE2 do not cause this effect by influencing COL1 expression, but by inhibiting collagen degradation and thus contributing to increased extracellular matrix deposition (Bergheim et al, 2006; Qi et al, 2008). This effect is confirmed at the SERPINE1 mRNA level, which increases considerably upon E2F1 knockdown (Fig. 4H and EV7F,G). Furthermore, the RT-qPCR data support the hypothesis that E2F1 has an indirect effect on collagen deposition, as the expression of COL1A1 mRNA is only moderately affected by E2F1 knockdown, in contrast to the observations for FLI1 (Figs. 4H and EV7F,G).

In summary, the integration of extensive time-resolved multi-omics data into mechanistic networks in combination with phenotypic and molecular validation experiments can serve as a basis to develop working models for molecular mechanisms underlying kidney fibrosis, as demonstrated in this study for FLI1 and E2F1.

## Discussion

In this study, we present an integrative approach to investigate the complex molecular mechanisms underlying kidney fibrosis. By combining a perturbable human PDGFRβ^+^ mesenchymal cell in vitro model system with time-resolved multi-omics profiling and advanced computational analyses, we have generated a comprehensive dataset that provides unprecedented insights into the dynamic nature of fibrotic processes driven by these cells.

Our in vitro model system enables detailed phenotypic and molecular characterization of fibrosis in PDGFRβ^+^ mesenchymal cells, addressing many limitations of existing approaches that either focused on a limited number of readouts or time points or exerted a much lower coverage of the omics modalities (Eddy et al, 2020; D’Souza et al, 2014; Arif et al, 2023; Lassé et al, 2023; Zhou et al, 2020a; Bouwens et al, 2025). The ability to observe and quantify phenotypic consequences of TGF-β stimulation, such as COL1/ECM deposition within a 96-h timeframe demonstrates the accelerated nature of our system and are in line with previous studies using macromolecular crowding (Rønnow et al, 2020; Chen et al, 2009; Coentro et al, 2021; Khan et al, 2024). This rapid induction of a fibrotic phenotype allows for more efficient studies of potential therapeutic interventions and enables us to link long-term patient data, spanning years, with in vitro data obtained over the course of hours. This is showcased by comparison with patient-derived scRNAseq data (Kuppe et al, 2021) that revealed that many of the deregulated factors identified in our study are myofibroblast-specific genes. This association between in vitro and in vivo data strengthens the potential of our model system for identifying clinically relevant therapeutic targets and bridges the gap between experimental and clinical observations (Kuppe et al, 2021).

The multi-omics approach, encompassing transcriptomics, proteomics, phosphoproteomics, and secretomics, has allowed us to quantify over 14,000 biomolecules across multiple time points, of which 2435 were significantly affected in at least one condition. The temporal resolution of our data has uncovered distinct dynamics in the expression and activity of known and potentially novel biomarkers and modulators of fibrosis, highlighting the importance of time-dependent analyses in understanding disease mechanisms to provide personalized approaches which is further supported by previous studies (Reznichenko et al, 2024; Rasmussen et al, 2019; Cisek et al, 2016).

A key strength of our approach is the ability to distinguish between the abundance and activity of molecular players. This distinction is particularly evident in our analysis of transcription factor and kinase activities (Dugourd and Saez-Rodriguez, 2019), which has revealed novel insights into the regulatory networks driving fibrosis.

Our multi-omics approach, combined with network modeling and experimental validation, revealed critical regulatory mechanisms that single-omics studies (Eddy et al, 2020; D’Souza et al, 2014; Arif et al, 2023; Lassé et al, 2023; Zhou et al, 2020a) might overlook.

Perturbation experiments of our in vitro model system through siRNA knockdown experiments allowed us to validate the computational predictions and explore the functional roles of specific factors in fibrosis. An example of this is the unexpected finding that knockdown of several early-activated transcription factors such as E2F1, FLI1, SMAD1, NR4A1, and BHLHE40 leads to increased collagen deposition. This further suggests that these factors may act as negative regulators of fibrosis, thereby opening new avenues for therapeutic intervention.

Further investigations into the molecular mechanism of E2F1-mediated regulation revealed a complex regulatory network involving E2F1 in both early and late TGF-β responses. Initially, E2F1 functions downstream of RELA, while in the later phase, its activity appears to be downregulated through a TGFB1-PAX8 signaling axis (Chaves-Moreira et al, 2022; Li et al, 2011). This is also in line with earlier studies using these cells that show that E2F1 activity fluctuates post TGF-β treatment (Bouwens et al, 2025).

Notably, E2F1 inhibition leads to increased expression of SERPINE1 (PAI-1), promoting collagen accumulation by preventing collagen degradation. This mechanism provides insight into how E2F1 regulation may influence extracellular matrix composition through post-translational control of collagen turnover.

Similarly, our network analysis uncovered the regulatory mechanism of another key transcription factor, FLI1, which operates through a MAPK1-RELA-HDAC signaling axis downstream of TGFB1, as suggested by our network model. This finding is particularly significant as it affirms previous studies identifying FLI1 as a collagen production inhibitor regulated by HDAC1 (Mikhailova et al, 2023). Together, these findings suggest that both E2F1 and FLI1 act as crucial negative regulators in fibrosis through distinct but potentially interconnected pathways.

While our study provides valuable insights, it also has limitations. Despite the power of our integrative approach, there are still aspects that we do not fully understand, such as the precise mechanisms causing the downregulation of the described transcription factors. Additionally, our network model provides valuable insights and potential downstream mechanisms, but these need to be thoroughly validated. In contrast, there could also be a variety of interesting potential mechanisms reflected in the data that are missing in the computational network model because it optimizes for a balance of size and signal, and therefore contains incomplete parts.

Furthermore, while our temporal profiling provides a detailed view of early fibrotic events, these are in vitro and need to be confirmed with patient data, such as that from such as the Kidney Precision Medicine Project (KPMP) (Lake et al, 2023) to fully understand the chronic nature of kidney fibrosis, bridging the gap between our in vitro findings and clinical observations.

As this study focuses on characterizing TGF-β-induced ECM production in vitro, it cannot fully recapitulate the complex multicellular interactions present in the kidney. While the used cell culture system is based on cells that co-express PDGFRα and PDGFRβ and can resemble the mesenchymal origin of myofibroblasts, their full in vivo heterogeneity cannot be reflected by a single cell line (Bouwens et al, 2025). Future studies could address this by incorporating co-culture systems, organoid models (Lassé et al, 2023; Piossek et al, 2022) or precision-cut kidney slices (Poosti et al, 2015; Bigaeva et al, 2019, 2020; Stribos et al, 2016) to better reflect the in vivo environment.

To contextualize our findings within the broader landscape of fibrotic disease mechanisms, we compared our kidney cell data with multi-omics datasets from human lung fibroblasts and ex vivo lung tissue slices treated with TGF-β (Khan et al, 2024, 2021). We detected a common response to TGF-β stimulation across cell culture and organ systems in terms of differential expression and TF activity levels for longer treatment durations (Fig. EV4). The early TF activation processes that we further investigated in the kidney cell culture system are not present in the lung datasets. This is likely due to the fact that these early time points were uniquely studied in our work here, and emphasizes the power of longitudinal approaches in multi-omic studies. The presence of both shared and distinct transcriptional regulators highlights fundamental fibrotic pathways that operate across organs and supports the generalizability of the presented data to study fibrosis-related processes. As expected, given the differences in the organ systems, the experimental setups used, and the technical variability of the methods applied in our study, the Spearman correlation values did not exceed a low to moderate range as reported in other studies (Blank et al, 2020; Morrow et al, 2019). It will be important though to validate and complement our study in the future in systems which are closer to the organ level in animal models or patient tissues.

In conclusion, our integrative, time-resolved multi-omics approach provides a comprehensive view of the molecular events driving kidney fibrosis. By combining advanced experimental and computational methods, we have generated a rich resource for the renal research community and demonstrated the power of systems biology approaches in unraveling complex disease mechanisms. The insights gained from this study not only advance our understanding of kidney fibrosis but also pave the way for the development of novel therapeutic strategies targeting this challenging condition. Future work leveraging the comprehensive data of this study has the potential to impact the clinical management of chronic kidney disease and other fibrotic disorders.

## Methods

**Table:** Reagents and tools table

Reagent/resource	Reference or source	Identifier or catalog number
**Cell culture reagents**		
DMEM (Dulbecco’s Modified Eagle Medium) 1 g/L d-glucose, l-Glutamine, 110 mg/L Sodium Pyruvate	Gibco	31885
DMEM (Dulbecco’s Modified Eagle Medium) 1 g/L d-glucose, no glutamine, no phenol red	Gibco	11880
FBS (fetal bovine serum)	Gibco	A5256701
L-Glutamine solution	Sigma Life Science	G7513
Ficoll® PM 770 kDa	Sigma-Aldrich	F2878
Ficoll® PM 400 kDa	Sigma-Aldrich	F4375
Recombinant Human TGF-beta 1 Protein	R&D systems	240-B-010
BSA (Bovine Serum Albumin)	Sigma-Aldrich	A2153
L-Ascorbic Acid 2-phosphate (magnesium salt hydrate)	Cayman Chemical Company	16457
Trypsin-EDTA 0.05%	Gibco	25300-054
AmbionTM Nuclease-Free Water, DEPC-Treated	Life Technologies Corp.	AM9906
DMSO (Dimethyl sulfoxide)	Sigma-Aldrich	D2438
ScreenFect® Dilution Buffer	ScreenFect	S-2001
ScreenFect® siRNA	ScreenFect	S-4001
**Antibodies and fluorescent dyes**		
Collagen Type I Antibody, rabbit polyclonal	Rockland	600-401-103-0.5
Anti-GFP, rabbit polyclonal	Origene	TP401
phospho-SMAD2 (Ser465/Ser467) (E8F3R), rabbit monoclonal	Cell Signaling	#18338
SMAD2, rabbit polyclonal	Proteintech	12570-1-AP
Tubulin-α AB-2, mouse monoclonal	Thermo Scientific	MS581
Phalloidin AlexaFluor 647	Invitrogen	A22287
CNA35-GFP	EMBL protein expression core facility	
Hoechst H33342	Sigma	B2261
Goat anti-mouse IgG secondary antibody, Oregon green 488	Thermo Fisher Scientific	O-11033
Goat anti-rabbit-IgG AlexaFluor 488	Molecular Probes	A11008
Goat anti-rabbit AlexaFluor 647	Invitrogen	A21245
Rabbit anti-mouse peroxidase	Sigma	A9044
Goat anti-rabbit peroxidase	Sigma	A0545
**Oligonucleotides and other sequence-based reagents**		
qPCR Primers	This study	Table EV1
siRNAs	This study	Table EV2
**Chemicals, enzymes, and other reagents**		
PierceTM RIPA Buffer	ThermoFisher Scientific	89900
NuPAGE MOPS SDS running buffer (20X)	ThermoFisher Scientific	NP0001
Total RNA Purification Kit	Norgen Biotek Corp.	17200
RNeasy® Mini Kit	Qiagen	74104
RNase-Free DNase Set	Qiagen	79256
DTT (DL-Dithiothreitol) solution	Sigma-Aldrich	3/12/3483
Ethanol	Merck	100983
Random Hexamer Primers	Invitrogen by ThermoFisher Scientific	N8080127
RNaseOUTTM Recombinant Ribonuclease Inhibitor	Invitrogen by ThermoFisher Scientific	10777019
dNTP mix, 10 mM each	Thermo Scientific	R0193
SuperScriptTM IV Reverse Transcriptase	Invitrogen by ThermoFisher Scientific	18090200
SuperScriptTM III	Invitrogen by ThermoFisher Scientific	18080093
SYBRTM Green PCR Master Mix	Applied Biosystems by ThermoFisher Scientific	4309155
RNaseZap™ RNase Decontamination Solution	ThermoFisher Scientific	AM9780
Bovine Serum Albumin (BSA)	Sigma-Aldrich	A2153
Dimethyl sulfoxide (DMSO)	Sigma-Aldrich	D2438
DL-Dithiothreitol (DTT)	Sigma-Aldrich	D0632
EDTA-Free Protease Inhibitor Cocktail (PIC)	Roche	11873580001
Ethanol	Merck	1.00983
Ethylenediaminetetraacetic acid (Na2EDTA)	Merck Millipore	324503
Glycerine	VWR	56-81-5
Glycine	Merck	1.04201
Methanol	Merck	322415
Paraformaldehyde (PFA)	ThermoFisher Scientific	50-980-491
Precision plus protein prestained standard marker	Bio-Rad	1610394
Sodium Chloride (NaCl)	Merck	1.06404
Sodium Dodecyl Sulfate (SDS) 20% Solution	Bio-Rad	1610418
Sodium Hydroxide (NaOH) Merck	Merck	1.06498
SYBRTM Gold Nucleic Acid Gel Stain	ThermoFisher Scientific	S11494
SYBRTM Safe DNA Gel Stain	ThermoFisher Scientific	S33102
Trichloroacetic acid (Tris-HCl)	Merck	1.0081
Triton X-100	Sigma-Aldrich	T8787
Trizma base	Sigma-Aldrich	T1503
Tween-20	Sigma-Aldrich	P7949
**Software**		
Adobe Acrobat	Adobe Systems Incorporated, San Jose, USA	
Adobe Illustrator	Adobe Systems Incorporated, San Jose, USA	
Cytoscape	Institute of Systems Biology, Seattle, USA	
Fiji ImageJ	Johannes Schindelin, Max Planck Institute of Molecular Cell Biology and Genetics, Dresden, Germany, and others	
Microsoft Office	Microsoft Corporation, Redmond, USA	
R 4.4.1	Comprehensive R Archive Network (CRAN)	
StepOne Software v2.3	ThermoFisher Scientific	
QuantStudio™ Real-Time PCR Software v1.3	ThermoFisher Scientific	
R Studio, Version 2024.04.2 + 764	Posit Software, PBC	
Python 3	Create Space, Scotts Valley, CA	
**Other**		
NuclonTM Delta Surface 96-well plate	Thermo Scientific	167008
NuclonTM Delta Surface 24-well plate	Thermo Scientific	142475
NuclonTM Delta Surface 6-well plate	Thermo Scientific	140675
NuclonTM Delta Surface 10 cm dish	Thermo Scientific	150350
NuclonTM Delta Surface 15 cm dish	Thermo Scientific	168381
Glass bottom imaging plate 24-well	Cellvis	P24-1.5H-N
Millipore® Stericup® Vacuum Filtration System	Millipore	S2GPU05RE
Cool Cell freezing container	Corning	CLS432004
Cryotubes	Thermo Scientific	363401
MicroAmpTM Fast Optical 96-Well Reaction Plate	Applied Biosystems by Life Technologies	4346906
MicroAmpTM Optical Adhesive Film	Thermo Fisher Scientific	4311971
Applied Biosystems™ MicroAmp™ Optical 384-Well Reaction Plate with Barcode	Applied Biosystems by Life Technologies	4343814
PCR strips of 8 tubes 0–2 ml	Ratiolab	8610040
StepOne Real-Time PCR system	Thermo Fisher Scientific	
QuantStudio 6 Flex	Applied Biosystems by Life Technologies	
T100 Thermal Cycler	Bio-Rad Laboratories	
Centrifuge 5417 R	Eppendorf	
Centrifuge 5408 R	Eppendorf	
Vortex-Genie 2	Scientific Industries	
Mini-Centrifuge ROTILABO®	Carl Roth	
Analytical balance TE124S-OCE	Sartorius	
Tilt/roller mixer RS-TR05	Phoenix Instruments	
ThermoMixer C	Eppendorf	
Scale	VWR	
Rotary shaker	neoLab	
Azure 280 Western blot imaging system	Azure Biosystems	
ChemiDoc TM Touch Imaging System	Bio-Rad	
Tissue culture incubator	Binder	
Thermo Scientific Heraeus® BBD6220 incubator	Thermo Scientific	
Water bath	GFLR	
Magnetic stirring hotplate MR3001 K	Heidolph	
Mini Trans-blot Cell Blotting system	Bio-Rad	
Mini Trans-blot Cell system	Bio-Rad	
Nanodrop 8000 Spectrophotometer	Thermo Scientific	
Protein Gel Electrophoresis XCell SureLock System	ThermoFisher Scientific	
Infinite M1000 pro plate reader	Tecan	
Automated widefield screening microscope Molecular Devices IXM	IXM	
Confocal high-throughput Microscope LSM 900	Zeiss	

No blinding was performed for the experiments. LLM (Claude 3.5 Sonnet and ChatGPT-4) were used for formulations of text and editing.

### Cell biology

#### Ethics

The local ethics committee of the University Hospital RWTH Aachen approved the generation of cells (EK-016/17). Kidney tissue was collected from the Urology Department of the Hospital Eschweiler from patients undergoing (partial) nephrectomy (Kuppe et al, 2021; Bouwens et al, 2025). All patients provided informed consent, and the study was performed in accordance with the Declaration of Helsinki.

#### Cell lines and reagents

Human kidney PDGFRβ^+^ mesenchymal cells, isolated from a 71-year-old male patient with normal eGFR, were received from the Kramann lab (for further information, please check (Kuppe et al, 2021; Bouwens et al, 2025)) and cultured in low glucose DMEM growth medium (Gibco 31885) supplemented with 5% FBS (Gibco A5256701). Cells were maintained at 37 °C in a humidified incubator with 5% CO2 and passaged approximately three times a week. All experiments were carried out between passages 33 and 36. Mycoplasma testing was routinely conducted, yielding negative results.

#### Experimental setup

For multi-omics and time point experiments, the medium in all conditions was changed 24 h post-plating. The longest time point treatment (e.g., 96 h) was initiated the day after seeding. Control samples were maintained in low glucose DMEM without phenol red (Gibco 11880), supplemented with 1% L-Glutamine (Sigma Life Science G7513), Ficoll 70 and 400 (Sigma-Aldrich F2878 and F4375), and 500 μM l-ascorbic acid 2-phosphate (Cayman Chemical Company 16457). TGF-β-treated samples received the same medium with an additional 10 ng/ml of recombinant human TGF-β1 (R&D Systems 240-B-010). Until the treatment started and for the 0 h treatments, the cells were kept in DMEM without phenol red, FBS, Ficoll, or ascorbic acid. Media changes were performed daily, with specific treatments applied as described to ensure all samples were ready for downstream processing at the same time.

#### siRNA transfections

All siRNAs used are described in Dataset EV7 and were acquired from Ambion/Thermo Fisher. Cells were plated one day before siRNA transfections, targeting 40-50% confluency on the day of treatment. The ScreenFect®siRNA protocol was employed. For a single 24-well plate reaction, 1 μl of ScreenFect®siRNA reagent (ScreenFect S-4001) was mixed with 39 μl of Dilution Buffer (ScreenFect S-2001). Separately, 5 pmol siRNA was diluted with 39 μl of Dilution Buffer. The mixtures were combined, incubated for 20 min at room temperature, and then 420 μl of fresh DMEM (without FBS) was added. The transfection mixture replaced the cell medium, which was changed to DMEM + 5% FBS after 5–6 h. For knockdown and TGF-β treatment experiments, cells were cultured for 48 h post-siRNA transfection in DMEM + 5% FBS before starting TGF-β treatments, with media changes every 24 h. Cells were fixed/harvested at specified time points.

#### Immunofluorescence assays

Cells were seeded on glass-bottom 24-well plates (Cellvis P24-1.5H-N) and treated as described above. After aspirating the medium, cells were washed with PBS and fixed with 4% PFA (Thermo Fisher Scientific 50-980-491) containing 1:1000 Hoechst 33342 (Thermo Fisher Scientific H21492) for 10–15 min at room temperature. Following three PBS washes, cells were used for immunofluorescence staining.

#### Extracellular immunofluorescence staining

For extracellular matrix (ECM) visualization, cells were incubated with anti-COL1 antibody (Rockland 600-401-103-0.5, 1:500 in PBS) for 1–1.5 h at room temperature, washed, and then incubated with fluorescently labeled secondary anti-rabbit IgG AlexaFluor 488 (Molecular Probes A11008, 1:400 in PBS) in PBS for 30–45 min. Washed cells were kept in PBS and imaged. Due to issues with new batches of the anti-COL1 antibody, GFP-labeled CNA35 dye (EMBL protein expression facility, 1:250 in PBS) was used for validation experiments (siRNA knockdowns of TFs). After fixation and washing, cells were incubated with CNA35 for 1–1.5 h, washed, and imaged. In cases of increased autofluorescence from siRNA transfection, cells were stained with an anti-GFP (Origene TP401) followed by Alexa 647-conjugated secondary anti-rabbit (Invitrogen A21245).

#### Intracellular immunofluorescence staining

Intracellular proteins, such as actin, were visualized by permeabilizing cells with 0.1% Triton X-100 (Sigma-Aldrich T8787) for 15 min post-fixation. Subsequent incubation with Phalloidin AlexaFluor 647 (Invitrogen A22287), washing, and confocal imaging were performed as described for extracellular staining.

### Microscopy

#### Wide-field microscopy

High-throughput imaging was conducted using the Molecular Devices IXM automated widefield screening microscope. A total of 36 fields of view were captured per well using a CFI P-Apo 20x Lambda/0.75 objective. Nuclear signals were acquired in the Hoechst channel (Ex: 377/50, Em: 477/60), with ECM signals in the GFP channel (Ex: 472/30, Em: 520/35) or other channels depending on the secondary antibody used (such as Cy5 with Ex:28/40-25 Em: 692/40-25).

#### Confocal microscopy

Confocal microscopy was performed using a Zeiss LSM 900 microscope. For visualization of cell cytoskeleton changes (actin filaments), Z-stacks were acquired covering the entire cell thickness, with parameters and number of Z-stacks kept constant across samples. The microscope was equipped with a Plan-Apochromat 20x/0.8 M27 air objective (FWD = 0.55 mm). Three lasers were utilized: 405 nm (5 mW), 488 nm (10 mW), and 640 nm (5 mW). For detection, a Gallium Arsenide Phosphide-PMT (GaAsP-PMT) was used for fluorescence. The filter configuration included excitation filters BP 385/30 for DAPI/Hoechst, BP 469/38 for FITC/AlexaFluor 488, and BP 631/33 for Cy5. A QBS 405 + 493 + 575 + 653 beam splitter was employed, along with an emission filter QBP 425/30 + 514/30 + 592/25 + 709/100.

### Biochemistry

#### Cell lysis and sample preparation

Cells were washed twice with ice-cold PBS and lysed in Pierce RIPA buffer (Thermo Fisher Scientific 89900) with EDTA-Free Protease Inhibitor Cocktail (Roche 1836170001) for 5 min on ice. Lysates were centrifuged at 14,000 × *g* for 15 min at 4 °C. Supernatants were used for analysis or stored at −80 °C. Protein samples were mixed with 2× sample buffer (200 mM Tris-HCl (Sigma-Aldrich T5941), 25% glycerol (v/v) (Sigma-Aldrich 15523-1L-R), 11.25% SDS (v/v) (Bio-Rad Laboratories 1610394), 325 mM DTT (Sigma-Aldrich D0632), 0.0125% (w/v) bromophenol blue (Sigma-Aldrich B5525), pH 6.8) and heated at 98 °C for 5 min.

#### SDS-PAGE and western blot

Protein samples were separated on NuPAGE 4-12% Bis-Tris gels (Thermo Fisher Scientific NP0321, NP0322) using NuPAGE MOPS SDS running buffer (Thermo Fisher Scientific NP0001) at 90–100 V for 200 min. Proteins were transferred to Immobilon PVDF membranes (pore size 0.45 μM, Merck Millipore IPVH00010) for 1 h at 100 V. Membranes were blocked with 5% BSA (Sigma-Aldrich A2153) in TBS-T (Thermo Scientific J60764.K2)), were incubated with primary antibodies (Tubulin-α, Thermo Scientific MS-581-P0; SMAD2, Proteintech 12570-1-AP; phospho-SMAD2, Cell Signaling 18338) 1 h at room temperature or overnight at 4 °C. After washing, the membranes were treated with HRP-coupled secondary antibodies (Anti-mouse IgG, Sigma-Aldrich A9044; Anti-rabbit IgG, Sigma-Aldrich A0545) for 45 min at room temperature. Proteins were visualized using Pierce^TM^ ECL Plus Western Blotting Substrate (Thermo Scientific 32132) and imaged with Azure 280 (Biozyme) or Bio-Rad imager. Bands were quantified using Fiji ImageJ and normalized to loading controls.

#### RNA Isolation and RT-qPCR

RNA was extracted using the RNeasy Mini kit (Qiagen 74104) according to the manufacturer’s instructions. RNA concentration and purity were measured with Nanodrop 8000 Spectrophotometer (Thermo Fisher Scientific). For RT-qPCR, 300–500 ng total RNA was reverse transcribed using SuperScript IV Reverse Transcriptase (Thermo Fisher Scientific 18090200). cDNA was diluted 1:10 and amplified using SYBR Green PCR Master Mix (Applied Biosystems by Thermo Fisher Scientific 4309155) on StepOne Real-Time PCR system (Thermo Fisher Scientific) or QuantStudio 6 Flex systems (Bio-Rad Laboratories).

Data were analyzed using the 2^-ΔΔCT^ method (Livak and Schmittgen, 2001), normalizing to GAPDH. Primer sequences are described in Table EV2.

### Multi-omics experiment

#### RNAseq

Cell lysis and RNA isolation were performed using the Total RNA Purification Kit (Norgen Biotek Corporation 17200) according to the manufacturer’s protocol. On-column DNA removal was conducted using Norgen’s RNase-Free DNase I Kit (Norgen Biotek Corporation 25710) following the manufacturer’s instructions.

The initial RNA was QCed using Agilent Bioanalyzer with the RNA Nano Assay kit as per the manufacturer’s protocol. The RNA sample set was then standardized to 300 ng total RNA in 50 µl using the concentration values given by the Bioanalyzer. The libraries were prepared on a Beckman Coulter Automated Workstation Biomek i7 Hybrid (MC +Span-8). For library preparation, an automated version of the NEBNext® Ultra^TM^ II Directional RNA Library Prep Kit was used, following section 1 - Protocol for use with NEBNext Poly(A) mRNA Magnetic Isolation Module. An adapter dilution of 1 to 20 was used, the samples were individually barcoded using unique dual indices during the PCR using 13 PCR cycles as per the manufacturer’s protocol. The individual libraries were quantified using the Qubit HS DNA assay as per the manufacturer’s protocol. For the measurement 1 µl of sample in 199 µl of Qubit working solution was used. The quality and molarity of the libraries were assessed using an Agilent Bioanalyzer with the DNA HS Assay kit as per the manufacturer’s protocol. The assessed molarity was used to equimolarly combine the individual libraries into one pool for sequencing. The pool was loaded and sequenced on an Illumina NextSeq 2000 platform (Illumina, San Diego, CA, USA) using a P3 50 cycle kit, a read-length of 72 bp single-end reads and 650 pM final loading concentration.

#### Proteomics experiments

Proteomics samples were lysed on-plate and processed by the Proteomics Core Facility.

**Sample preparation:** For all samples, protein concentration was determined using the Pierce BCA Protein Assay Kit (Thermo Fisher Scientific 23227). Working reagent and BSA standards were prepared, following the manufacturer’s protocol. 25 μl of standards and 2 μl of TCA-precipitated samples were added to NuclonTM Delta Surface 96-well plate (Thermo Fisher Scientific 167008) in triplicates. In total, 200 μl of working reagent was added to each well. Plates were incubated at 37 °C for 30 min after 1 min of shaking. Absorbance was measured at 562 nm using an Infinite M1000 pro plate reader (Tecan). Protein concentrations were calculated using a standard curve, and samples were diluted to achieve equal protein amounts across all samples.

For the secretomics experiments, cell culture supernatants were collected and centrifuged at 300 × *g* for 5 min at 4 °C to remove cells. Cleared supernatants were snap-frozen and stored at −80 °C until further processing. Proteins were enriched using TCA precipitation. Therefore, one part ice-cold TCA (Merck 1008071000) was added to four parts of the protein sample. Samples were subsequently vortexed and incubated on ice for 20–30 min. This was followed by centrifugation at 10,000 × *g* for 20 min at 4 °C. Pellets were washed with 500 μl ice-cold 10% TCA, vortexed, and centrifuged at max speed for 20 min at 4 °C. A final wash was performed with 1 ml ice-cold acetone (−20 °C) (Merck 100014), followed by centrifugation at max speed for 30 min at 4 °C. Dried pellets were dissolved in 25 μl 1% SDS buffer (1% SDS (Bio-Rad 1610418), 50 mM HEPES (Gibco 15630-080) in DEPC-treated water (Ambion AM9906)). Reduction of disulphide bridges in cysteine-containing proteins was performed with dithiothreitol (56 °C, 30 min, 10 mM in 50 mM HEPES, pH 8.5). Reduced cysteines were alkylated with 2-chloroacetamide (room temperature, in the dark, 30 min, 20 mM in 50 mM HEPES, pH 8.5). Samples were prepared using the SP3 protocol (Hughes et al, 2019, 2014) and trypsin (sequencing grade, Promega) was added in an enzyme-to-protein ratio 1:50 for overnight digestion at 37 °C. The next day, peptide recovery in HEPES buffer by collecting supernatant on the magnet and combining with second elution wash of beads with HEPES buffer. Peptides were labeled with TMT11plex (Werner et al, 2014) Isobaric Label Reagent (ThermoFisher) according to the manufacturer’s instructions. Samples were combined for the TMT11plex and for further sample clean up an OASIS® HLB µElution Plate (Waters) was used. Offline high-pH reverse phase fractionation was carried out on an Agilent 1200 Infinity high-performance liquid chromatography system, equipped with a Gemini C18 column (3 μm, 110 Å, 100 × 1.0 mm, Phenomenex) (Reichel et al, 2016).

For the full proteome and phosphoproteome experiments, phosphopeptide enrichment was essentially done as described in Potel et al, 2018 (Potel et al, 2018). Cells were lysed on the plate with 500 µl lysis buffer composed of 100 mM Tris-HCl pH 8.5, 7 M Urea, 1% Triton, 5 mM Tris(2-carboxyethyl)phosphin-hydrochlorid, 30 mM chloroacetamide, 10 U/ml DNase I (Sigma-Aldrich), 1 mM magnesium chloride, 1 mM sodium orthovanadate, phosphoSTOP phosphatase inhibitors (Sigma-Aldrich) and complete mini EDTA-free protease inhibitors (Roche). The samples were sonicated three times for 10 s (continuous pulse, 50% duty cycle) with intervals of cooling on ice for 60 s until the viscosity was reduced with an ultrasonic sonifier (Branson). Residual cell debris was removed by centrifugation at 17,000 × *g* at 8 °C for 10 min. To the supernatant, 1% benzonase (Merck Millipore) was added and incubated at RT for 1 h. Then methanol/chloroform precipitation was performed by adding to one volume of sample four volumes of methanol, one volume of chloroform and three volumes of ultrapure water. Centrifugation was performed for 15 min at 4000 × *g*. The upper layer was removed without disturbing the interface and three volumes of methanol were added before another centrifugation step. The liquid phase was removed, and the white protein precipitate was allowed to air dry. Proteins were resuspended in digestion buffer composed of 100 mM Tris-HCl pH 8.5, 1% sodium deoxycholate (Sigma-Aldrich), 5 mM Tris(2-carboxyethyl)phosphin-hydrochloride and 30 mM chloroacetamide. Trypsin was added to a 1:50 ratio (w/w), and protein digestion was performed overnight at RT. The next day, digestion was stopped by the addition of TFA to a final concentration of 1% in the sample. The sodium deoxycholate was precipitated for 15 min at RT and the samples were centrifuged for 10 min at 17,000 × *g* at RT. The supernatant was desalted by using Oasis HLB 96-well plates 30 µM (Waters). Thereby, buffer A was composed of MS-grade water (Chemsolute) with 0.1% formic acid and buffer B 80% acetonitrile (Chemsolute) in MS-grade water with 0.1% formic acid. Eluted peptides were dried in a vacuum centrifuge.

For phosphopeptide enrichment, peptides were taken up in IMAC loading solvent (70% acetonitrile, 0.07% TFA). Of each sample, a small aliquot was used for full proteome analysis. Phosphopeptide enrichment was performed on an UltiMate 3000 RSLC LC system (Dionex) using a ProPac IMAC-10 Column 4 × 50 mm, P/N 063276 (Thermo Fisher Scientific). To enable post-enrichment TMT labeling, the phosphopeptides were eluted with 0.4% dimethylamine (Sigma-Aldrich). Peptides were labeled with TMT16plex (Thompson et al, 2019) Isobaric Label Reagent (ThermoFisher) according to the manufacturer’s instructions. In short, 0.8 mg reagent was dissolved in 42 µl acetonitrile (100%) and 4 µl of stock was added and incubated for 1 h room temperature. Followed by quenching the reaction with 5% hydroxylamine for 15 min. RT. Samples were combined, and for further sample clean up an OASIS® HLB µElution Plate (Waters) was used. The TMT-labeled phosphoproteome and full proteome were fractionated by high-pH reversed-phase carried out on an Agilent 1200 Infinity high-performance liquid chromatography system, equipped with a Gemini C18 column (3 μm, 110 Å, 100 ×1.0 mm, Phenomenex). 48 fractions were collected along with the LC separation that were subsequently pooled into 12 fractions. Pooled fractions were dried under vacuum centrifugation and reconstituted in 10 μL 1% formic acid, 4% acetonitrile, and then stored at −80 °C until LC-MS analysis.

**Mass spectrometry measurements** For the secretomics experiments, an UltiMate 3000 RSLC nano LC system (Dionex) fitted with a trapping cartridge (µ-Precolumn C18 PepMap 100, 5 µm, 300 µm i.d. × 5 mm, 100 Å) and an analytical column (nanoEase™ M/Z HSS T3 column 75 µm × 250 mm C18, 1.8 µm, 100 Å, Waters). Trapping was carried out with a constant flow of 0.05% trifluoroacetic acid at 30 µL/min onto the trapping column for 6 min. Subsequently, peptides were eluted via the analytical column with a constant flow of solvent A (0.1% formic acid, 3% DMSO in water) at 0.3 µL/min with increasing percentage of solvent B (0.1% formic acid, 3% DMSO in acetonitrile). The outlet of the analytical column was coupled directly to a Fusion Lumos (Thermo) mass spectrometer using the Nanospray Flex™ ion source in positive ion mode. The peptides were introduced into the Fusion Lumos via a Pico-Tip Emitter 360 µm OD × 20 µm ID; 10 µm tip (CoAnn Technologies) and an applied spray voltage of 2.4 kV. The capillary temperature was set at 275 °C. A full mass scan was acquired with a mass range 375–1500 *m/z* in profile mode in the Orbitrap with resolution of 120,000. The filling time was set at a maximum of 50 ms with a limitation of 4 ×10^5^ ions. Data-dependent acquisition (DDA) was performed using quadrupole isolation at 0.7 *m/z*, the resolution of the Orbitrap set to 30,000 with a fill time of 94 ms and a limitation of 1 × 10^5^ions. A normalized collision energy of 36 was applied. MS2 data was acquired in profile mode.

For the phosphoproteomics experiments, an UltiMate 3000 RSLC nano LC system (Dionex) fitted with a trapping cartridge (µ-Precolumn C18 PepMap 100, 5 µm, 300 µm i.d. × 5 mm, 100 Å) and an analytical column (nanoEase™ M/Z HSS T3 column 75 µm × 250 mm C18, 1.8 µm, 100 Å, Waters) was coupled to a Orbitrap Fusion™ Lumos™ Tribrid™ Mass Spectrometer (Thermo). Peptides were concentrated on the trapping column with a constant flow of 0.05% trifluoroacetic acid at 30 µL/min for 6 min. Subsequently, peptides were eluted via the analytical column using a binary solution system at a constant flow rate of 0.3 µL/min. Solvent A consists of 0.1% formic acid in water with 3% DMSO and solvent B of 0.1% formic acid in acetonitrile with 3% DMSO. The percentage of solvent B was increased as follows: from 2 to 4% in 4 min, to 8% in 2 min, to 25% in 64 min, to 40% in 12 min, to 80% in 4 min, followed by re-equilibration back to 2% B in 4 min (for the phosphoproteome). For the full proteome analysis, the steps were as follows: from 2 to 8% in 4 min, to 28% in 104 min, to 40% in 4 min, to 80% in 4 min, followed by re-equilibration back to 2% B in 4 min. The peptides were introduced into the Fusion Lumos via a Pico-Tip Emitter 360 µm OD × 20 µm ID; 10 µm tip (New Objective) and an applied spray voltage of 2.4 kV. The capillary temperature was set at 275 °C. Full mass scan was acquired with mass range 375–1400 *m/z* for the phosphoproteome (375–1500 *m/z* for the full proteome), in profile mode in the Orbitrap with resolution of 120,000. The filling time was set at a maximum of 50 ms for the full proteome with a limitation of 4 × 10^5^ ions. Data-dependent acquisition (DDA) was performed with the resolution of the Orbitrap set to 30,000, with a fill time of 110 ms for the phosphoproteome (94 ms for the full proteome) and a limitation of 1 × 10^5^ ions. A normalized collision energy of 34 was applied. MS2 data was acquired in profile mode. Fixed first mass was set to 110 *m/z*.

### Data analysis

If not stated otherwise, statistical analysis was performed using the R programming language (version 4.4.1) in the RStudio environment (version 2024.04.2 + 764).

#### Image analysis of wide-field microscopy images

Image inspection and analysis, including nuclei segmentation and fluorescence quantification, were performed using Fiji ImageJ (Schindelin et al, 2012) and CellProfiler (Stirling et al, 2021). Details of the CellProfiler pipeline and parameters are provided in the GitHub repository (CellProfiler Pipeline). Key steps included image loading, channel assignment, nuclei segmentation, and fluorescence measurement. Data was exported to CSV files for downstream analysis.

CellProfiler output was imported into R (R Foundation for Statistical Computing, 2021) for further analysis. Treatments were assigned based on well and position information extracted from the filenames. Fluorescence intensity values were rescaled and background subtracted. Autofluorescence and non-fibrillar ECM staining corrections were applied. Intensity values were then normalized to the number of nuclei in each image, calculating a per-nucleus intensity value. Images with low nuclei counts were excluded from the analysis.

#### COL1 deposition analysis

A linear mixed model with random intercept was applied to analyze COL1 deposition over time, accounting for treatment (TGF-β or control), time (0, 12, 24, 48, 72, and 96 h), and their interaction as fixed effects, with plate (biological replicate) set as random effect. The model formula in R notation is:

$${sqrt\_mean\_intensity} \sim {condition}* {time}+(1{|plate}).$$

The 0 h time point values were duplicated to account for the absence of a 0 h TGF-β treatment. The model includes:

- An intercept (control condition at 0 h, varying per plate)
- A conditionTGF parameter (TGF-β treatment effect)
- Time parameters for each time point (time 12 h, time 24 h, etc.)
- Interaction terms between time and condition (conditionTGF: time12 h, conditionTGF: time24 h, etc.)

The treatment effect at any given time point is the sum of the relevant parameters (e.g., for TGF-β at 12 h: intercept + condition TGF + time 12 h + conditionTGF:time 12 h).

Average COL1 intensity per cell was calculated for each condition, technical, and biological replicate. A square root transformation was applied to stabilize the variance and improve residual distribution (Piepho, 2009). Analysis of variance using type III sum of squares, followed by a post hoc test, was performed to identify significant differences between factor levels. For visualization, sqrt_mean_int data was normalized per plate by subtracting the 0 h time point values. The resulting data points and model-predicted values were plotted (Fig. 1C). The underlying data can be found in Dataset EV8.

#### Analysis of ECM Deposition of siRNA validation experiments

For siRNA treatments, ECM staining per cell was normalized to the siNeg9 (non-targeting siRNA) control:

$${ratio}=\frac{{ECM}/{{cell}}_{{siRNA}}-{ECM}/{{cell}}_{{siNeg}9}}{{ECM}/{{cell}}_{{siNeg}9}}$$

This normalization was performed separately for control and TGF-β-treated samples, per plate (biological replicate). For TGF-β-treated samples, an additional ratio was calculated by dividing siRNA + TGF-β-treated samples by siNeg9 + TGF-β samples. Statistical significance was determined using unpaired two-sided *t* tests. The underlying data can be found in Dataset EV5.

#### RT-qPCR data statistical testing

For RT-qPCR analysis in the validation experiments (Results section “Experimental validation confirms the implication of selected TFs in regulating ECM deposition”), data preprocessing involved removing technical replicates with standard deviation >0.5 if clearly outliers. The 2^-ΔΔCT^ method (Livak and Schmittgen, 2001) was applied for analysis. CT values were normalized to GAPDH as the housekeeping gene, and ΔΔCT values were calculated relative to the siNeg9 control sample at 48 h post siRNA transfection. mRNA expression was presented as percentages, with the control set to 100%. Statistical analysis included unpaired two-sided *t* tests comparing 72 h and 96 h samples (siRNA control vs. siNeg9 control, and siRNA TGF-β vs. siNeg9 TGF-β). It should be noted that more technical replicates of siNeg9 samples were included as reference samples across different RT-qPCR plates, resulting in a higher number of data points for these controls in the analysis. The underlying data can be found in Dataset EV6.

#### RNAseq alignment and preprocessing

Sequencing reads were aligned using STAR (version 2.7.9a) with default parameters on GRCh38. The gene count tables were produced during the alignment (--quantMode GeneCounts) using the annotation GRCh38.93. One outlier sample (ctrl, 24 h, replicate A) was removed, and one sample swap was corrected (24 h and 48 h TGF-β treated, replicate B) because of experimental errors. Lowly expressed genes were removed using the filterByExpr() function of the edgeR R package. Normalization factor calculation and normalization were performed using the calcNormFactors() and cpm() functions of the edgeR R package.

#### MS database search

For the secretomics data, all raw files were converted to mzML format using MSConvert from Proteowizard (version 3.0.22129), using peak picking from the vendor algorithm. Files were then searched using MSFragger v3.7 (Kong et al, 2017) in Fragpipe v19.1 against the Swissprot Homo sapiens database (20,594 entries) containing common contaminants and reversed sequences. The standard settings of the Fragpipe TMT11 workflow were used. The following modifications were included into the search parameters: Carbamidomethyl (C) and TMT11 (K) (fixed modification), Acetyl (Protein N-term), Oxidation (M) and TMT11 (N-term) (variable modifications). For the full scan (MS1) a mass error tolerance of 10 ppm and for MS/MS (MS2) spectra of 0.02 Da was set. Further parameters were set: Trypsin as protease with an allowance of maximum two missed cleavages and a minimum peptide length of seven amino acids was required. The false discovery rate on peptide and protein level was set to 0.01.

For the phosphoproteomics data, MSFragger v3.8 (Kong et al, 2017) was used to process the acquired data, which was searched against the homo sapiens Uniprot proteome database (UP000005640, ID9606, 20594 entries, release October 2022) with common contaminants and reversed sequences included. The following modifications were considered as fixed modification: Carbamidomethyl (C) and TMT16 (K). As variable modifications: Acetyl (Protein N-term), Oxidation (M) and TMT16 (N-term), for the phosphoproteome specifically phosphorylation on STY. For the MS1 and MS2 scans a mass error tolerance of 20 ppm was set. Further parameters were: Trypsin as protease with an allowance of maximum two missed cleavages; Minimum peptide length of seven amino acids; at least two unique peptides were required for a protein identification. The false discovery rate on the peptide and protein level was set to 0.01.

#### Proteomics preprocessing

For the secretomics data, the raw output files of MSFragger (Kong et al, 2017) (protein.tsv – files) were processed using the R programming language (R Foundation for Statistical Computing, 2021). Contaminants including albumin were filtered out and only proteins that were quantified with at least two unique peptides were considered for the analysis. Moreover, only proteins which were identified in two out of three mass spec runs were kept. 2188 proteins passed the quality control filters. For the phosphoproteomics data, the raw output files of MSFragger (Kong et al, 2017) (psm.tsv for phospho data and protein.tsv files for input data) were processed using the R programming language. Only peptide spectral matches (PSMs) with a phosphorylation probability greater 0.75 and proteins with at least 2 unique peptides were considered for the analysis. Phosphorylated amino acids were marked with a * in the amino acid sequences behind the phosphorylated amino acid, labeled with a 1, 2, or 3 for the number of phosphorylation sites in the peptide, and concatenated with the protein ID in order to create a unique ID for each phosphopeptide. Raw TMT reporter ion intensities were summed for all PSMs with the same phosphopeptide ID. For the input data, the reporter ion intensities were used as given in the protein.tsv output files. Phospho signals were also normalized by input abundance. For this, the reporter ion intensity for each unique phospho ID, condition, and replicate was normalized according to the following formula:

$${Norm}.{{Intensity}}_{{phospho},{gene},{condition}}=\frac{\frac{{{Intensity}}_{{phospho},{gene},{condition}}}{{{Intensity}}_{{input},{gene},{condition}}}}{\frac{{median}\left({{Intensity}}_{{phospho},{gene}}\right)}{{median}\left({{Intensity}}_{{input},{gene}}\right)}}{median}\left({{Intensity}}_{{phospho},{gene}}\right)$$

For all proteomics data modalities, transformed summed TMT reporter ion intensities or log2 transformed raw TMT reporter ion intensities were first cleaned for batch effects using the “removeBatchEffects” function of the limma package (Ritchie et al, 2015) and further normalized using the vsn package (variance stabilization normalization (Huber et al, 2002)). For observations with less than 30% missing values in the phosphoproteomics data, missing values were imputed with the “knn” method using the Msnbase package (Gatto and Lilley, 2012).

#### Differential abundance analysis

Proteins and transcripts were tested for differential expression using the limma package. For the proteomics data modalities, phospho, normalized phosphor, and input data were tested separately. For the secretomics data, the replicate information was added as a factor in the design matrix given as an argument to the ‘lmFit’ function of limma. Resulting *P* values were adjusted for multiple testing. A protein was considered statistically significantly different with an adjusted *P* value below 0.05 and log2 fold change above log2(1.5) or below log2(1/1.5). A transcript was considered statistically significantly different with an adjusted *P* value below 0.05 and log2 fold change above log2(2) or below log2(2). For the secretomics data, the proteins were filtered for secreted proteins using the gene sets “M5889” and “M5885” from the MSIGDB (Bhuva et al, 2024).

#### Correlation analyses

Technical (Pearson) correlation between all samples and replicates was computed using the counts and reporter intensities before differential expression analysis. To correlate significantly affected molecules between datasets and conditions, the t-values of proteins that were affected in at least one condition were correlated using Pearson correlation, with the exception of the phosphoproteomics data, for which this has been done separately.

#### Pathway enrichment analysis

For the path enrichment analysis, MSIGDB (Bhuva et al, 2024) Hallmark pathways were used with the decoupleR package (Badia-I-Mompel et al, 2022) (normalized weighted average method) to calculate pathway enrichment scores from log2 fold change values. This algorithm estimates an enrichment score that corresponds to the mean signal or an alternative summary statistic of all annotated pathway members.

#### Enzyme activity analysis

In order to estimate the activity of different input nodes (transcription factors (TFs), kinases, phosphatases) the normalized weighted mean and mixed linear model (mlm) methods of the decoupleR package were used. For transcription factors, the DOROTHEA database was used to obtain TF-target interactions with a confidence level of A, B, or C (Garcia-Alonso et al, 2019). The interactions were downloaded using the OmnipathR package (Türei et al, 2016). Phosphosite–enzyme collections were likewise downloaded with OmnipathR. For the decoupleR tool, log2 fold-change values of transcripts and phosphosites after limma analysis were used as input per condition. A TF, kinase or phosphatase was considered to be significantly affected for *P* value < 0.03 and absolute normalized enrichment score >3.

#### Network modeling

The primary goal of the network modeling was to extract a subnetwork that could consistently connect nodes from different data modalities in both sign and direction, using a template PKN as a starting point using COSMOS (Dugourd et al, 2021). The PKN contains the signed and directed protein-protein interactions (node A activates/inactivates node B) which are used to represent signaling interactions between root nodes (input nodes) and downstream nodes (measurement nodes) The subnetwork is selected through an integer linear programming (ILP) formulation that solves a particular instance of the Prize-Collecting steiner tree problem with multiple root nodes and additional custom constraints (see GitHub Repo).

In this study, we used a prior knowledge network which is provided as part of the OmnipathR package using the import_all_interactions() function. This initial PKN was filtered for trusted resources and interactions with a valid consensus signal. In addition, the core of the TGFb signaling module was fixed to guide the optimization (enforce TGFb to SMAD1-5, support TGFb to MAPK1, MAPK14, AKT1, and PI3K) following Park and Yoo (Park and Yoo, 2022). The final PKN (42k interactions) was obtained by filtering for expression, downstream neighbors, and consistent TF-target interactions as reported before (Dugourd et al, 2021).

As input we used significant transcription factors and kinases/phosphatase from the activity estimation analysis which we divided into early and late groups (0.08 h, 1 h, 12 h and 24 h, 48 h, 72 h, 96 h) as well as secretomics hit filtered for proteins which are known to be secreted (0.08 h, 1 h, 12 h, 24 h for early network, 48 h, 72 h, 96 h for late network). This list was manually extended with COL1A1, COL5A1, SERPINE2, SPARC, ITGB1, VIM, JUP, ACTA1, HSPG2, LOXL2, TNC, TGFBI, IGFBP3, IGFBP7, LTBP2, TAGLN, CCN2, LRATD2, MRC2, FN1, FBN1, BGN, ADGRG1, MMP2, ITGA11, AMIGO2, ADAM12, CPA4, DCDC2, PLEKHG4 and ITGB1BP1 which were significantly deregulated in the secretomics experiment and reported to be secreted before. The number of input nodes has been chosen to find an acceptable compromise between computational cost and information content for the network modeling step.

In total, four subnetworks were inferred based on different time points and input modalities to account for the time-resolved nature of the obtained data. To start the optimization, we used the stimulating agent TGFb as the upstream input node of early affected enzymes. This network was combined with a second network connecting the set of early affected enzymes and early secretomics hits. This combination represents early signaling events stimulated by TGFb treatment which then lead to altered protein secretion up to 24 h (early network). To model the assumed autocrine function of secreted factors, we modeled later signaling processes by inferring a first network between secreted proteins included in the early network and enzymes affected at a later time point (between 24 h and 96 h). In a last step, this network was combined with a fourth subnetwork connecting later affected enzymes with late secretomics hits (late network).

#### Network-level analyses

Node enrichment analysis for the early and late network was performed using ReactomePA (Gillespie et al, 2022). The enrichment results were compared by calculating the log2 odds ratio for each pathway. Closeness centrality was computed per subnetwork using the closeness centrality method implemented in the igraph R-package (http://igraph.org).

## Supplementary information

Table EV1

Table EV2

Peer Review File

Dataset EV1

Dataset EV2

Dataset EV3

Dataset EV4

Dataset EV5

Dataset EV6

Dataset EV7

Dataset EV8

Source Data Fig EV2

Expanded View Figures

## Data Availability

The datasets and computer code produced in this study are available in the following databases: The mass spectrometry proteomics data have been deposited to the ProteomeXchange Consortium via the PRIDE partner repository with the dataset identifier PXD056096. The transcriptomics sequencing data have been deposited to the ArrayExpress repository with the ArrayExpress accession E-MTAB-14521. Microscopy data are available in the BioStudies database (https://www.ebi.ac.uk/biostudies/) under accession number S-BIAD1415 (10.6019/S-BIAD1415). All code used to perform the computational analyses described and to reproduce the figures is available at GitHub (https://github.com/saezlab/kidneyfibrosis_multiomicsmodel_paper). All figures and analyses can be reproduced using the provided supplementary tables (contain processed data and analysis results) and the source data provided on GitHub. The source data of this paper are collected in the following database record: biostudies:S-SCDT-10_1038-S44320-025-00116-2.
