# Supplementary material for: Dynamic multi-omics and mechanistic modeling approach uncovers novel mechanisms of kidney fibrosis progression
Source: Mol Syst Biol. 2025 Jun 5;21(8):1030–65. doi: 10.1038/s44320-025-00116-2 (PMC12322177; doi:10.1038/s44320-025-00116-2)
Supplement: Supplementary file 1 — Table EV1 [file 44320_2025_116_MOESM1_ESM.pdf]

molecular systems biology

Dynamic multi-omics and mechanistic modeling approach uncovers novel mechanisms of kidney fibrosis progression

Nadine Tuechler, Mira Burtscher, Martin Garrido-Rodriguez, Muzamil Khan, Dénes Türei, Christian Tischer, Sarah Kaspar, Jennifer Schwarz, Frank Stein, Mandy Rettel, Rafael Kramann, Mikhail Savitski, Julio Saez-Rodriguez, and Rainer Pepperkok

Corresponding author(s): Rainer Pepperkok (pepperko@embl.de) , Julio Saez-Rodriguez (saezrodriguez@ebi.ac.uk)

Review Timeline:

Transfer from Review Commons:	3rd Feb 25
Editorial Decision:	5th Feb 25
Revision Received:	6th Mar 25
Editorial Decision:	4th Apr 25
Revision Received:	18th Apr 25
Accepted:	25th Apr 25

Review COMMONS

Editor: Poonam Bheda

Transaction Report: This manuscript was transferred to Molecular Systems Biology following peer review at Review Commons.

Review #1

1. Evidence, reproducibility and clarity:

Evidence, reproducibility and clarity (Required)

Summary

This study showed measurements and integration of time-series multiple omics data of the human kidney PDGFR beta+ cells responding to TGF-beta stimuli. The authors also presented key pathways that were inferred based on estimating activities of TFs and kinases, and confirmed by knockdown experiments whose phenotypes can be observed by means of imaging.

Major concerns

1. The content of Discussion is too thin. Particularly, it is uncommon to see a discussion section with no citations like this manuscript. Cite related studies and compare with the own results so that the authors can argue originality and novelty of this work. I also see some citations in Results. Usually it is opposite: little citations in Results section and many citations in Discussions.
2. Put more emphasis on presenting biological relevances in order for readers to easily recognize them. I guess that Figs. 4C and 4F are examples of such biological findings.
3. Draw the whole picture(s) of the integrated networks, not only subnetworks. If too much complicated, the complexity itself will be important information for readers.
4. On SMAD2:

4a) The responses of p-SMAD2 in Fig. S2 are remarkably different in the two batches. The authors should discuss the reason of these outcomes. Which of the two batches exhibited similar responses to the phosphoproteome data?

4b) What possible reasons do authors think about that SMAD2/3 are not included in the transcriptional regulatory networks presented in Figs. 3 and 4 in spite of their importance in the TGFbeta signaling? Should be argued.

4c) What molecular mechanism can cause the increase in SERPINE1 expression dependent on TGFbeta? The mechanism may involve SMAD2/3 but neither presented nor argued. Should be clarified.

4d) It seems inconsistent that knockdown of the early-activated TFs cause extensive ECM accumulation in the knockdown experiment presented in Fig. 4B. Did the authors see suppression of ECM accumulation by knockdown of SMAD2/3? Should be presented.

Minor concerns

1. Fig. 1D: Numbers in the Venn diagram of 'proteomics technologies' do not match with the numbers in another Venn diagram on the right hand side. Should be corrected or explained.
2. Fig. 2B: 'INFalpha' should be IFNalpha, so is 'INFgamma'.
3. Fig. 2B, Fig. S4C: What does the sign of 'Pathway enrichment score' mean? How is it calculated? Should be explained.
4. Do not fit curves to data that should be drawn in line graphs (e.g. Figs. 3F, 4E, 4G etc.).
5. How did the authors plot the regression curves presented in Fig. 4D? Should be clarified.
6. What is 'PKN'? Maybe 'Prior Knowledge Network', but clearly spelled out when it first appears.
7. Did the PNK-nodes in the networks exhibit quantitative changes in any of the omics data?
8. What do the axes of the heatmaps mean in Fig. S3A? Why are there more categories than total sample numbers? Should be clarified.

2. Significance:

Significance (Required)

The omics data were well measured under appropriate quality controls. Hence, this study will attract interests from specialists of kidney fibrosis and systems biologists. But there still remains concerns regarding arguments and data presentation of the manuscript.

3. How much time do you estimate the authors will need to complete the suggested revisions:

Estimated time to Complete Revisions (Required)

(Decision Recommendation)

Between 1 and 3 months

4. *Review Commons* values the work of reviewers and encourages them to get credit for their work. Select 'Yes' below to register your reviewing activity at Web of Science Reviewer Recognition Service (formerly Publons); note that the content of your review will not be visible on Web of Science.

Web of Science Reviewer Recognition

Yes

Review #2

1. Evidence, reproducibility and clarity:

Evidence, reproducibility and clarity (Required)

Summary:

The authors presented a comprehensive, time-resolved multi-omics analysis of kidney fibrosis using an in vitro model system based on human kidney PDGFRβ+ mesenchymal cells aimed at unraveling disease mechanisms. This research advanced our understanding of the pathogenesis of kidney fibrosis. However, this reviewer has several concerns.

Major comments:

1.Why does the 0.08h group not exist in Fig S1? What's more, the detection of ECM appears to be insufficient as it only reveals COL1 expression.
2.Fig S2A shows that p-smad2 has 11 bands, whereas Smad2 has 12 bands. Moreover, the repeatability of the two repeated trials is not very excellent. Additionally, why not look at the phosphoproteomics data to see how p-smad2 changes?
3.The early-activated transcription factors screened by the author, including FLI1 and E2F1, act as negative regulators of collagen deposition, needs further verification.

Minor comments:

1.The graphical abstract and the abstract don't agree on how many time points there are-is it seven or eight?
2.For every group in the multi-omics, what is the n value?

2. Significance:

Significance (Required)

The insights gained from this study not only advance our understanding of kidney fibrosis but also pave the way for the development of novel therapeutic strategies targeting this

challenging condition. There is still much to be done, though. For instance, the author's screening of early-activated transcription factors, such as FLI1 and E2F1, which function as negative regulators of collagen deposition, requires additional confirmation.

3. How much time do you estimate the authors will need to complete the suggested revisions:

Estimated time to Complete Revisions (Required)

(Decision Recommendation)

Between 1 and 3 months

4. *Review Commons* values the work of reviewers and encourages them to get credit for their work. Select 'Yes' below to register your reviewing activity at Web of Science Reviewer Recognition Service (formerly Publons); note that the content of your review will not be visible on Web of Science.

Web of Science Reviewer Recognition

Yes

Review #3

1. Evidence, reproducibility and clarity:

Evidence, reproducibility and clarity (Required)

In this study the authors sought to identify novel mechanisms underlying the progression of kidney fibrosis, by activating myofibroblast formation of a human kidney fibroblast cell line with TGF-beta, and collecting a time-series data set of transcriptome, proteome, phosphoproteome and secretome. They then performed a number of computational analyses to identify the key pathways and regulators that were driving the TGF-beta mediated responses in the early and late time points. They further validated several candidates experimentally with siRNA knockdowns, confirming FLI1 and E2F1 as two primary suppressors for myofibroblast activation.

Major comments: while all the experiments and data collections appeared to be carried out carefully, all data essentially came from one human PDGFRβ+ cell line derived from a previous study. Can this cell line fully represent the fibroblast populations in human

kidneys? I could not find much information such as donor age, sex, or clinical conditions of the donor. It is unclear how much the cell line has been passaged, what is the level of clonality or the level of replication-induced senescence. How can we ensure that the mechanisms identified from one single cell line are robust and generalizable, truly representative of common kidney fibroblast cells or fibroblasts in general? The amount of multi-omics data collection was quite impressive, and I don't think it is realistic to repeat all those data generation experiments across multiple cell lines. Nonetheless, I feel that it is important to selectively validate some of the key findings on additional cell lines. On a related note, myofibroblast activation can be different between male and female in vivo and in vitro (https://www.biorxiv.org/content/10.1101/2024.10.02.615251v1.abstract). Is any of the findings in this study sex specific?

Minor comments:

Results section 2.1. Authors state "Specifically, we observed the activation of myofibroblast-specific gene expression as the fibrotic process progresses linking long-term patient data with in vitro data obtained over the course of hours". However, the transcriptomic data (Figure 1F) shows very low # of hits for these myofibroblast specific genes. Does this indicate that these cells are already in the myofibroblast state and that this is a model for TGFB stimulation of myofibroblasts? More clarification on this and what is being modeled (including starting and ending state of these cells) is needed.
The authors tend to overstate how this in vitro model reflects complex disease phenotypes. The main issue is what is being modeled, which appears to be mostly TGF-B induced ECM production and possibly enhanced myofibroblast state signatures? On page 23: "To summarize, the integration of multi-omic data into time-resolved network models of early and late fibrotic responses revealed dynamic shifts in signaling pathways, transcription factor activities, and protein interactions, highlighting the temporal complexity of kidney fibrosis progression and identifying both well-known and novel regulatory factors for further investigation." Here it is not clear that the timeline used in this paper is recapitulating "late fibrotic processes" seen in vivo nor how it truly relates to kidney fibrosis progression. Also section 2.4: "To further validate the role of these transcription factors in the development of fibrotic diseases…". This is not something that this in vitro model can achieve.
In section 2.4, the paragraph discussing E2F1 is poorly written, over uses the word "activity", and is not clear.
Figure 3E: it is a bit of surprise to see HDAC1 being a node there connecting RELA to KLF4/FLI1. HDAC1 deacetylates histones and many transcription factors, hence the effects are likely to be very broad. Can the authors explain why it has such a high specificity in this context?

2. Significance:

Significance (Required)

Overall, this is a nice study with several strengths. The time-series multi-omics data along the course of myofibroblast activation generated in this study is very impressive. While transcriptomic data collection is quite routine, the proteomics, phosphoproteomics, and secretomics data really lifted the significance of this study to another level. As demonstrated in their study, these data allowed the authors to carry out much more sophisticated computational analyses (which is another major strengths of this study), examining the responses in terms of gene regulation, protein production, modification, secretion at the early and late stages of fibrotic activation, formulating a mechanistic model. This study managed to get much closer to determining causal and direct regulation, compared with many other previous studies staying at the level of correlation and enrichments. Finally, some of the key regulators identified in their analyses were validated experimentally by siRNA knockdowns.

3. How much time do you estimate the authors will need to complete the suggested revisions:

Estimated time to Complete Revisions (Required)

(Decision Recommendation)

Between 1 and 3 months

4. *Review Commons* values the work of reviewers and encourages them to get credit for their work. Select 'Yes' below to register your reviewing activity at [Web of Science Reviewer Recognition Service] (formerly Publons); note that the content of your review will not be visible on Web of Science.

Web of Science Reviewer Recognition

Yes

Revision Plan

Manuscript number: RC-2024-02741
Corresponding author(s): Rainer Pepperkok, Julio Saez-Rodriguez

[The "revision plan" should delineate the revisions that authors intend to carry out in response to the points raised by the referees. It also provides the authors with the opportunity to explain their view of the paper and of the referee reports.

The document is important for the editors of affiliate journals when they make a first decision on the transferred manuscript. It will also be useful to readers of the reprint and help them to obtain a balanced view of the paper.

*If you wish to submit a full revision, please use our "Full Revision" template. **It is important to use the appropriate template to clearly inform the editors of your intentions.**]*

1. General Statements [optional]

This section is optional. Insert here any general statements you wish to make about the goal of the study or about the reviews.

Thank you for providing the opportunity to submit a revised version of our manuscript. We have substantially revised our manuscript to comprehensively address the valuable feedback from the reviewers.

Specifically, we have:

- improved the clarity of presentation by rewriting and removing or adapting vague and imprecise statements
- largely expanded our discussion
- improved the logic flow of the manuscript by adding information to support data analysis choices
- adapted figures as well as figures legends to enhance their interpretability

We believe that these modifications convey our message more clearly. We hope that our manuscript is now suitable for publication in one of your journals.

2. Description of the planned revisions

Insert here a point-by-point reply that explains what revisions, additional experimentations and analyses are planned to address the points raised by the referees.

Revision Plan

Most of the points made by the three reviewers have been addressed in our revised version of the manuscript.
However, we plan to expand on the generalisability of our work by comparing it to an existing lung fibrosis multi-omics dataset which has been recently published (Khan et al. 2024). This should allow us to further confirm some of our findings in an additional biological system and address comments of reviewers 2 and 3.

3. Description of the revisions that have already been incorporated in the transferred manuscript

Please insert a point-by-point reply describing the revisions that were already carried out and included in the transferred manuscript. If no revisions have been carried out yet, please leave this section empty.

Please find a point-by-point response (in *blue*, manuscript text in *italics*, major changes in red) to individual reviewer comments **at the end of this revision plan** (Answers 1.x for reviewer 1, Answers 2.x for reviewer 2, Answers 3.x for reviewer 3). Sections in the manuscript with major changes are marked in color red. Additionally, line numbers are provided for all text changes linking the response document to the revised manuscript.

4. Description of analyses that authors prefer not to carry out

Please include a point-by-point response explaining why some of the requested data or additional analyses might not be necessary or cannot be provided within the scope of a revision. This can be due to time or resource limitations or in case of disagreement about the necessity of such additional data given the scope of the study. Please leave empty if not applicable.

We addressed a point raised by reviewer 3 regarding validation in an additional cell line in depth in the attached response. In short, we believe finding a suitable model system to repeat this experiment is unfeasible given the uniqueness of the system used in this study.

Further, we believe that additional experiments validating the suggested molecular mechanisms for specific proteins suggested by reviewer 2 is interesting for future studies but out of scope in this case. We aimed to use system-level data to suggest a variety of testable mechanistic hypotheses rather than study single examples in depth.

Please see our suggestion for further analysis plans relating to this in “planned revisions”.

Revision Plan

Review COMMONS

Point-by-point response

Please note that we included an additional affiliation for one of the authors.
MMK: Translational Lung Research Center Heidelberg (TLRC), German Center for Lung Research (DZL), 69120 Heidelberg, Germany
We replaced Khan et al. 2023 with Khan et al. 2024.

Reviewer #1

Summary

This study showed measurements and integration of time-series multiple omics data of the human kidney PDGFR beta+ cells responding to TGF-beta stimuli. The authors also presented key pathways that were inferred based on estimating activities of TFs and kinases, and confirmed by knockdown experiments whose phenotypes can be observed by means of imaging.
The omics data were well measured under appropriate quality controls. Hence, this study will attract interests from specialists of kidney fibrosis and systems biologists. But there still remains concerns regarding arguments and data presentation of the manuscript.

Major concerns

1. The content of Discussion is too thin. Particularly, it is uncommon to see a discussion section with no citations like this manuscript. Cite related studies and compare with the own results so that the authors can argue originality and novelty of this work. I also see some citations in Results. Usually it is opposite: little citations in Results section and many citations in Discussions.

Answer 1.1
We agree with the reviewer's observation that the style of citation in the manuscript can be improved and has to be adapted. We made several adjustment in the results and discussion sections:

Revision Plan

Review COMMONS

Starting at **line 563** in the revised manuscript:

In this study, we present an integrative approach to investigate the complex molecular mechanisms underlying kidney fibrosis. By combining a perturbable human PDGFRβ+ mesenchymal cell in vitro model system with time-resolved multi-omics profiling and advanced computational analyses, we have generated a comprehensive dataset that provides unprecedented insights into the dynamic nature of fibrotic processes driven by these cells.

Our in vitro model system enables detailed phenotypic and molecular characterization of fibrosis in PDGFRβ+ mesenchymal cells, addressing many limitations of existing approaches that either focused on a limited number of readouts or time points or exerted a much lower coverage of the omics modalities (Arif et al. 2023; Bouwens et al. 2025; D'Souza et al. 2014; Eddy et al. 2020; Lassé et al. 2023; Zhou et al. 2020). The ability to observe and quantify phenotypic consequences of TGF-β stimulation, such as COL1/ECM deposition within a 96-hour timeframe demonstrates the accelerated nature of our system and are in line with previous studies using macromolecular crowding (Chen et al. 2009; Coentro et al. 2021; Khan et al. 2024; Rønnow et al. 2020). This rapid induction of a fibrotic phenotype allows for more efficient studies of potential therapeutic interventions and enables us to link long-term patient data, spanning years, with in vitro data obtained over the course of hours. This is showcased by comparison with patient-derived scRNAseq data (Kuppe et al. 2021) that revealed that many of the deregulated factors identified in our study are myofibroblast-specific genes. This association between in vitro and in vivo data strengthens the potential of our model system for identifying clinically relevant therapeutic targets and bridges the gap between experimental and clinical observations (Kuppe et al. 2021).

The multi-omics approach, encompassing transcriptomics, proteomics, phosphoproteomics, and secretomics, has allowed us to quantify over 14,000 biomolecules across multiple time points, of which 2,435 were significantly affected in at least one condition. The temporal resolution of our data has uncovered distinct dynamics in the expression and activity of known and potentially novel biomarkers and modulators of fibrosis, highlighting the importance of time-dependent analyses in understanding disease mechanisms to provide personalized approaches which is further supported by

previous studies (Cisek et al. 2016; Rasmussen et al. 2019; Reznichenko et al. 2021).

A key strength of our approach is the ability to distinguish between abundance and activity of molecular players. This distinction is particularly evident in our analysis of transcription factor and kinase activities (Dugourd & Saez-Rodriguez 2019), which has revealed novel insights into the regulatory networks driving fibrosis.

Our multi-omics approach, combined with network modeling and experimental validation, revealed critical regulatory mechanisms that single-omics studies (Arif et al. 2023; D'Souza et al. 2014; Eddy et al. 2020; Lassé et al. 2023; Zhou et al. 2020) might overlook, as exemplified in the following paragraphs.

Perturbation experiments of our in vitro model system through siRNA knockdown experiments allowed us to validate the computational predictions and explore the functional roles of specific factors in fibrosis. An example of this is the unexpected finding that knockdown of several early-activated transcription factors such as E2F1, FLI1, SMAD1, NR4A1, and BHLHE40 leads to increased collagen deposition. This further suggests that these factors may act as negative regulators of fibrosis, thereby opening new avenues for therapeutic intervention.

Further investigations into the molecular mechanism of E2F1-mediated regulation revealed a complex regulatory network involving E2F1 in both early and late TGF-β responses. Initially, E2F1 functions downstream of RELA, while in the later phase, its activity appears to be downregulated through a TGFB1-PAX8 signaling axis (Chaves-Moreira et al. 2022; Li et al. 2011). This is also in line with earlier studies using these cells that show that E2F1 activity fluctuates post TGF-β treatment (Bouwens et al. 2025).

Notably, E2F1 inhibition leads to increased expression of SERPINE1 (PAI-1), promoting collagen accumulation by preventing collagen degradation. This mechanism provides insight into how E2F1 regulation may influence extracellular matrix composition through post-translational control of collagen turnover.

Similarly, our network analysis uncovered the regulatory mechanism of another key transcription factor, FLI1, which operates through a MAPK1-RELA-HDAC signaling axis downstream of TGFB1, as suggested by our network model. This finding is particularly significant as it affirms previous studies identifying FLI1 as a collagen production inhibitor regulated by HDAC1 (Mikhailova et al. 2023). Together, these

findings suggest that both E2F1 and FLI1 act as crucial negative regulators in fibrosis through distinct but potentially interconnected pathways.
While our study provides valuable insights, it also has limitations. Despite the power of our integrative approach, there are still aspects that we do not fully understand, such as the precise mechanisms causing the downregulation of the described transcription factors. Additionally, our network model provides valuable insights and potential downstream mechanisms, but these need to be thoroughly validated. In contrast, there could also be a variety of interesting potential mechanisms reflected in the data that are missing in the computational network model because it optimises for a balance of size and signal and therefore contains incomplete parts.

Furthermore, while our temporal profiling provides a detailed view of early fibrotic events, these are in vitro and need to be confirmed with patient data, such as that from such as the Kidney Precision Medicine Project (KPMP) (Lake et al. 2023) to fully understand the chronic nature of kidney fibrosis, bridging the gap between our in vitro findings and clinical observations.
As this study focuses on characterising TGF-β induced ECM production in vitro, it cannot fully recapitulate the complex multicellular interactions present in the kidney. While the used cell culture system is based on cells that co-express PDGFRα and PDGFRβ and can resemble the mesenchymal origin of myofibroblasts, their full in vivo heterogeneity cannot be reflected by a single cell line (Bouwens et al. 2025). Future studies could address this by incorporating co-culture systems, organoid models (Lassé et al. 2023; Piossek et al. 2022) or precision-cut kidney slices (Bigaeva et al. 2019, 2020; Poosti et al. 2015; Stribos et al. 2016) to better reflect the in vivo environment.
In conclusion, our integrative, time-resolved multi-omics approach provides a comprehensive view of the molecular events driving kidney fibrosis. By combining advanced experimental and computational methods, we have generated a rich resource for the renal research community and demonstrated the power of systems biology approaches in unraveling complex disease mechanisms. The insights gained from this study not only advance our understanding of kidney fibrosis but also pave the way for the development of novel therapeutic strategies targeting this challenging condition. Future work leveraging the comprehensive data of this study has the potential to impact the

*clinical management of chronic kidney disease and other fibrotic disorder*s.

2. Put more emphasis on presenting biological relevances in order for readers to easily recognize them. I guess that Figs. 4C and 4F are examples of such biological findings.

Answer 1.2

We agree with the reviewer that we can emphasize the biological findings more and highlighted them further in the discussion as described in answer 1.1.. At the same time, we want to be careful with proposed biological mechanisms, to avoid overinterpretation of the obtained results. We considered this in our adapted version of the discussion.

3. Draw the whole picture(s) of the integrated networks, not only subnetworks. If too much complicated, the complexity itself will be important information for readers.

Answer 1.3

We thank the reviewer for this observation. The networks are included in Figure 3A and provided as Supplementary Table which can be explored in Cytoscape. We made adjustments in the Figure legend and the text to highlight this better.

Starting at **line 399** in the revised manuscript:

(A) Schematic representation of the chosen computational integration strategy. Two network models were created to reflect the early (network on the left) and late (right network) response to TGF-β stimulation integrating transcriptomics, phosphoproteomics and secretomics data for different time points.

4. On SMAD2:

4a) The responses of p-SMAD2 in Fig. S2 are remarkably different in the two batches. The authors should discuss the reason of these outcomes. Which of the two batches exhibited similar responses to the phosphoproteome data?

Answer 1.4a

We agree with the reviewer that the quantification of the two western blots is quite different. However, this experiment has only been performed as an initial qualitative quality control to confirm that canonical TGF-β signaling has been induced through the stimulation and to facilitate defining important time points for the multi-omics experiment. Unfortunately, the SMAD2/3 transcription factors are expressed at a rather low level, wherefore they were not detected in the phosphoproteomics experiments. To do so, a much deeper coverage would be required. We added this information to the results section as shown below.

Starting at **line 277** in the revised manuscript:

While most of these transcription factors show a constitutive up/down regulation over time, there are examples such as FLI1 with a temporally regulated activity that only increases after one hour of stimulation (Figure 2D). Note that this analysis allows assessment of TF activity, which can be difficult with direct measurement, as we for example were unable to detect activating SMAD2/3 phosphorylation in the phosphoproteome experiment, due to limited coverage of low-abundant proteins.

4b) What possible reasons do authors think about that SMAD2/3 are not included in the transcriptional regulatory networks presented in Figs. 3 and 4 in spite of their importance in the TGFbeta signaling? Should be argued.

Answer 1.4b

We agree with the reviewer that SMAD2/3 are central modulators in TGF-β signaling and an important part of the canonical pathway. The used network inference algorithm however optimises the combination of measured nodes with a PKN that is agnostic to pathway level information. This is the reason why topologies are often found that do not correspond to our textbook understanding of the underlying process. We tried to address this by fixing the SMAD molecules downstream of TGF-β as described in the Method section 4.5 Data analysis, Network modeling. As a result, SMAD2, SMAD1 and SMAD4 are

modeled downstream of TGF-β as shown in Figure S5. Exactly which successive edges are modeled then depends on a variety of reasons, e.g. the effect size of the observed signal, the prior knowledge coverage of the modeled nodes and the combination with all other input nodes. Therefore, the resulting networks must not be interpreted as a pathway visualisation but rather as an analysis result which can be used to explore and validate potential connections between different data modalities. We added a clarification in the result section of the manuscript.

Starting at **line 344** in the revised manuscript:
This provides a mechanistic molecular hypothesis for the observed ECM deposition at later time points, thus reflecting the dynamic nature of cellular communication (Figure 3A). As underlying PKN we used a directed and signed protein-protein interaction network retrieved from Omnipath (Türei et al. 2016, 2021). It should be noted that this modeling approach provides molecular associations that are not guided by commonly assumed pathway topologies and cannot be considered as a visualization of canonical pathways. This is in line with recent findings showing that the definition of so-called canonical pathways can be biased by the way biochemical research is done and is not necessarily useful to reflect signaling processes (Garrido-Rodriguez et al. 2024).

4c) What molecular mechanism can cause the increase in SERPINE1 expression dependent on TGFbeta? The mechanism may involve SMAD2/3 but neither presented nor argued. Should be clarified.

Answer 1.4c
We thank the reviewer for this question. Previous studies have linked the expression changes of SERPINE1 to MAPK as well as SMAD signaling (Ghosh & Vaughan 2012; Samarakoon et al. 2008). As described in Figure 4, our data as well as the multi-omics integration analysis suggest that the increase in SERPINE1 expression is related to the inactivation of the E2F1 transcription factor. We could show that siRNA mediated E2F1 knockdown induced SERPINE1 mRNA expression. The model suggests further regulation via the

transcription factor PAX8 which can be activated by TGF-β. However, this has not been validated further as it is out of scope for this study. We clarified this finding in the Discussion section of the manuscript, see Answer 1.1.

4d) It seems inconsistent that knockdown of the early-activated TFs cause extensive ECM accumulation in the knockdown experiment presented in Fig. 4B. Did the authors see suppression of ECM accumulation by knockdown of SMAD2/3? Should be presented.

Answer 1.4d

We thank the reviewer for this question. We did not focus on knockdown of SMAD2/3 as the canonical pathway of TGF-β is well-studied (D'Souza et al. 2014; Friedman et al. 2013; Huang et al. 2020; Meng et al. 2010, 2016; Zhao et al. 2022; Zi et al. 2012). As shown by others, knockdown of SMAD2 actually increases COL1 expression while knockdown of SMAD3 decreases it (e.g. Meng et. al 2010). This confirms the central role of SMAD2/3 in TGFB signaling, but also highlights the complexity of transcriptional reprogramming occurring during fibrogenesis. We directed our validation efforts on potential novel regulators that could play a role in kidney fibrogenesis besides the characterised canonical pathway.

We believe our assay allows us to make the presented conclusion, as (i) all knockdowns have been confirmed by qPCR, (ii) the knockdown of COL1A1 does reduce ECM accumulation as expected and (iii) for FLI1 and E2F1 we confirmed our results with a second siRNA. Further, one of the tested TFs (HNF4G) does reduce collagen deposition.

Minor concerns

1. Fig. 1D: Numbers in the Venn diagram of 'proteomics technologies' do not match with the numbers in another Venn diagram on the right hand side. Should be corrected or explained.

Answer 1.5

The reason for this is that a protein can be detected by multiple of the used proteomics technologies, but we only used the unique set of measured proteins to compare to the transcriptomics dataset.

2. Fig. 2B: 'INFalpha' should be IFNalpha, so is 'INFgamma'.

Answer 1.6

We thank the reviewer for this observation and have adapted it.

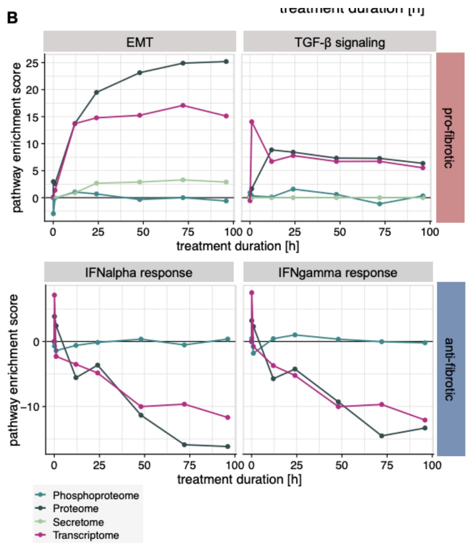

3. Fig. 2B, Fig. S4C: What does the sign of 'Pathway enrichment score' mean? How is it calculated? Should be explained.

Answer 1.7

We thank the reviewer for this question. The pathway enrichment analysis has been performed as described in the method section “pathway enrichment analysis” with the DecoupleR package (Badia-I-Mompel et al. 2022). This algorithm estimates an enrichment score which corresponds to the mean signal or an alternative summary statistic of all annotated pathway members. We extended the description in the Method section accordingly.

Starting at **line 1049** in the revised manuscript:

For the path enrichment analysis, MSIGDB (Bhuva et al. 2024) Hallmark pathways were used with the decoupleR package (Badia-I-Mompel et al. 2022) (normalized weighted average method) to calculate pathway enrichment scores from log2 fold change values. This algorithm estimates an enrichment score which corresponds to the mean signal or an alternative summary statistic of all annotated pathway members.

4. Do not fit curves to data that should be drawn in line graphs (e.g. Figs. 3F, 4E, 4G etc.).

Answer 1.8

We thank the reviewer for this question, but we do think a fit is appropriate to visualise time course data representing fold-changes or activity scores as it facilitates interpretation of the curve.

We extended our description in the figure legends, for example in **line 521** of the revised manuscript:

RT-qPCR data to confirm FLI1 knockdown effect on its potential downstream target COL1A1 +/-TGF-β stimulation at different time points. A temporal profile has been fitted using a loess fit.

5. How did the authors plot the regression curves presented in Fig. 4D? Should be clarified.

Answer 1.9

We thank the reviewer for this observation and have adapted our text accordingly for all plots with fits.

Starting at **line 518** in the revised manuscript:

RT-qPCR data to confirm FLI1 knockdown effect on its potential downstream target COL1A1 +/-TGF-β stimulation at different time points. A temporal profile has been fitted using a loess fit.

6. What is 'PKN'? Maybe 'Prior Knowledge Network', but clearly spelled out when it first appears.

Answer 1.10

We thank the reviewer for this observation and have adapted our text accordingly.

Starting at **line 335** in the revised manuscript:

We next integrated the findings obtained from the differential expression and kinase and TF activity analyzes in a network model, using a modified version of COSMOS, an optimisation method that identifies putative causal paths explaining changes in enzymes with altered activity and multi-omics measurements based on a causal Prior Knowledge Network (PKN) (Dugourd et al. 2021).

7. Did the PNK-nodes in the networks exhibit quantitative changes in any of the omics data?

Answer 1.11

We thank the reviewer for this comment. In total we have 8 PKN proteins which exhibit significant changes in abundance in at least one time point. One example is JUNB, a PKN node modelled to be active in the late network which also goes up in the RNA-seq data. It is not in the TFs considered significant, which could be due to many reasons we cannot assess e.g. data coverage or an activation in a temporal dynamic we did not cover with the chosen experimental design. All data related to this is accessible in the supplementary files S1 and S5.

Of note, we found many more changes at the activity level, illustrating the value of measuring different omics and deriving from them the activity; indeed activity of a protein is not necessarily reflected by its abundance (Szalai & Saez-Rodriguez 2020). This is the reason the network-model integration has been performed on activity not abundance level.

8 What do the axes of the heatmaps mean in Fig. S3A? Why are there more categories than total sample numbers? Should be clarified.

Answer 1.12

The reviewer raises an important point about Figure S3A. Each axis represents individual biological replicates by experimental condition (treatment and time

point), and shown per omics modality. We have adjusted the figure legend to provide better clarity.

Staring at **line 211** in the revised manuscript:

Heatmap showing Pearson correlation coefficients between individual biological replicates, calculated using TMT reporter intensities (proteomics/phosphoproteomics) or gene counts (transcriptomics). Each row/column represents a single biological replicate.

Review COMMONS

Reviewer #2

Summary

The authors presented a comprehensive, time-resolved multi-omics analysis of kidney fibrosis using an in vitro model system based on human kidney PDGFRβ+ mesenchymal cells aimed at unraveling disease mechanisms. This research advanced our understanding of the pathogenesis of kidney fibrosis. However, this reviewer has several concerns.

Major comments

1.Why does the 0.08h group not exist in Fig S1? What's more, the detection of ECM appears to be insufficient as it only reveals COL1 expression.

Answer 2.1

We thank the reviewer for this comment. We did not include the 0.08 h as well as the 1 h group in the imaging based experiments. The reason for this was that we did not expect significant changes in ECM deposition at these time points. COL1 synthesis and deposition takes time as it undergoes hydroxylation, glycosylation and cleavage by peptidases before being deposited (Karsdal et al. 2017; Khan et al. 2024). In fact, our data show that there was a significant increase of COL1 deposition after 24 h of TGF-β treatment compared to the corresponding control which was not observed at 12 h, the earliest time point tested (Figure 1).

Imaging of deposited COL1, as a major component of the fibrotic extracellular matrix (Karsdal et al. 2017; Lichtman et al. 2016), has been used in previous studies, such as (Khan et al. 2024), to successfully screen for anti-fibrotic drugs. Hence we used this assay as a phenotypic assay supporting our data generated using multi-omics and to identify potential regulators of kidney fibrosis.

To clarify, we stated in the Materials and Methods section, that our initial experiments were performed with an anti-COL1 antibody, while we used a

Review COMMONS

fluorescent dye binding to fibrotic ECM as new batches of the anti-COL1 did not work:

Starting at **line 699** in the revised manuscript:

For extracellular matrix (ECM) visualization, cells were incubated with anti-COL1 antibody (Rockland 600-401-103-0.5, 1:500 in PBS) for 1-1.5 hours at room temperature, washed, and then incubated with fluorescently labeled secondary anti-rabbit IgG AlexaFluor 488 (Molecular Probes A11008, 1:400 in PBS) in PBS for 30-45 minutes. Washed cells were kept in PBS and imaged. Due to issues with new batches of the anti-COL1 antibody, GFP-labeled CNA35 dye (EMBL protein expression facility, 1:250 in PBS) was used for validation experiments (siRNA knockdowns of TFs). After fixation and washing, cells were incubated with CNA35 for 1-1.5 hours, washed, and imaged. In cases of increased autofluorescence from siRNA transfection, cells were stained with an anti-GFP (Origene TP401) followed by Alexa 647-conjugated secondary anti-rabbit (Invitrogen A21245).

2.Fig S2A shows that p-smad2 has 11 bands, whereas Smad2 has 12 bands. Moreover, the repeatability of the two repeated trials is not very excellent. Additionally, why not look at the phosphoproteomics data to see how p-smad2 changes?

<u>Answer 2.2</u>

We appreciate the reviewer's feedback. The additional band in the SMAD2 blot is due to well spillover, while the 12th band represents a 72-hour siSMAD2 control sample used to validate antibody specificity. We adjusted the supplementary figure accordingly (see below).

While we acknowledge the quantitative differences between the replicate Western blots, these experiments served as qualitative quality controls to confirm canonical TGF-β pathway activation and guide time point selection for our multi-omics analysis. SMAD2/3 were not detected in our phosphoproteomics data due to their low abundance, which would require

substantially deeper phosphoproteome coverage for reliable detection. Please also refer to Answer 1.4a

Starting at **line 195** in the revised manuscript:

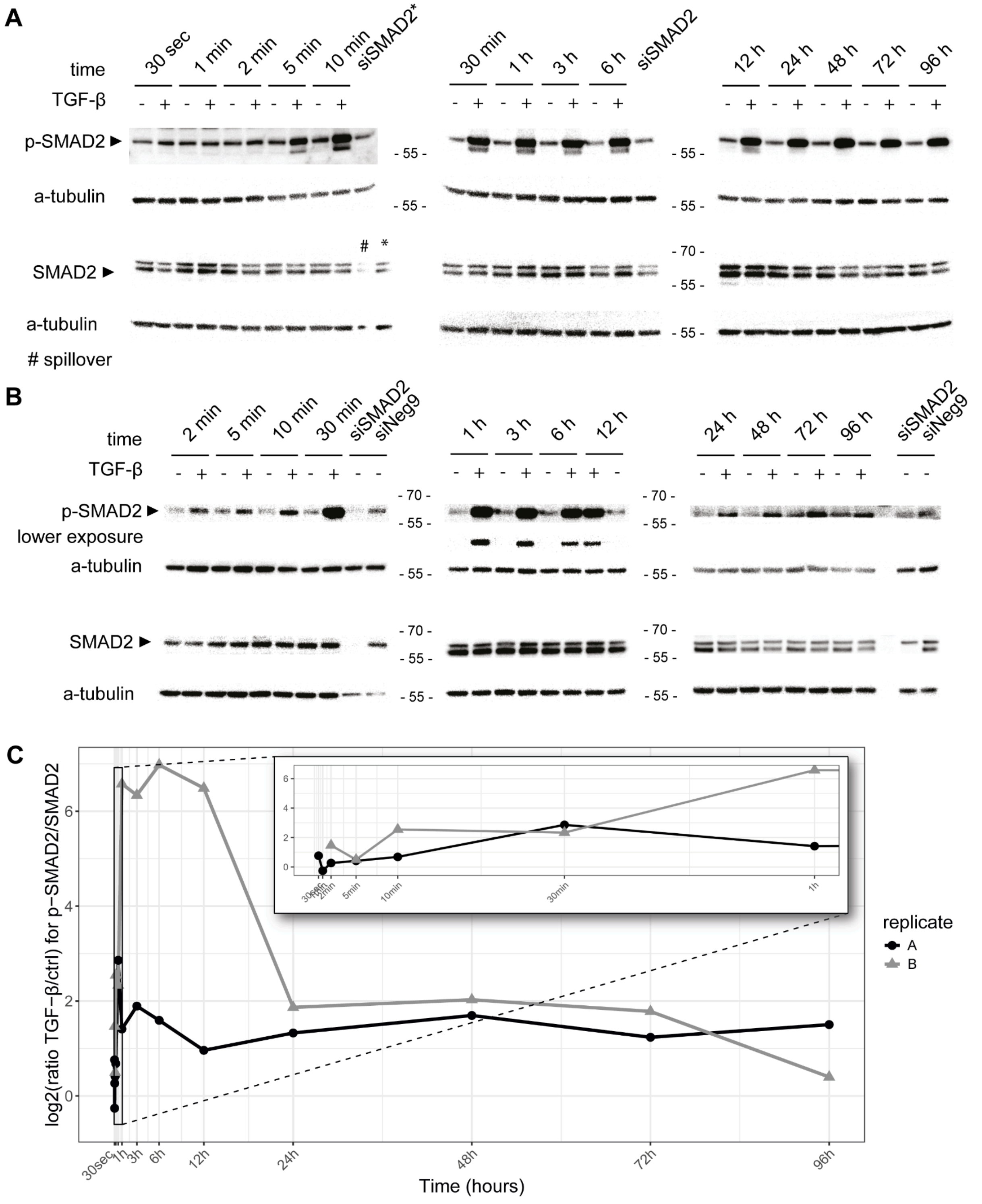

Supplementary Figure 2

(A) Western blot analysis of phosphorylated SMAD2 (p-SMAD2), total SMAD2, and α-tubulin (loading control) in response to TGF-β treatment over time. Time points range from 30 seconds to 96 hours. siSMAD2 condition is included as a control. A spill-over has been labelled with '#', the correct corresponding band and position has been labeled with ''.*

3. The early-activated transcription factors screened by the author, including FLI1 and E2F1, act as negative regulators of collagen deposition, needs further verification.

Answer 2.3
We agree with the reviewer that further verification of FLI1 and E2F1 is needed to prove their role as negative regulators in the context of kidney fibrosis. We plan to compare our data to a recently published lung fibrosis dataset to further support our findings. However, an in-depth verification is out of the scope of this study in which we wanted to suggest potential new regulators of fibrosis using an integrated analysis approach.

We also made this point in the discussion section, **line 629**:

While our study provides valuable insights, it also has limitations. Despite the power of our integrative approach, there are still aspects that we do not fully understand, such as the precise mechanisms causing the downregulation of the described transcription factors. Additionally, our network model provides valuable insights and potential downstream mechanisms, but these need to be thoroughly validated.

Minor comments

1.The graphical abstract and the abstract don't agree on how many time points there are-is it seven or eight?

Answer 2.4
We thank the reviewer for the observation. The time points included in this study are 0, 0.08, 1, 12, 24, 48, 72, and 96 hours (8 time points). In Fig 1, there are

only 7 lines but 8 time points mentioned, which is due to the fact that 0 and 0.08 h are so close together that we showed it as one line but aimed to illustrate all time points by mentioning them in the graph.

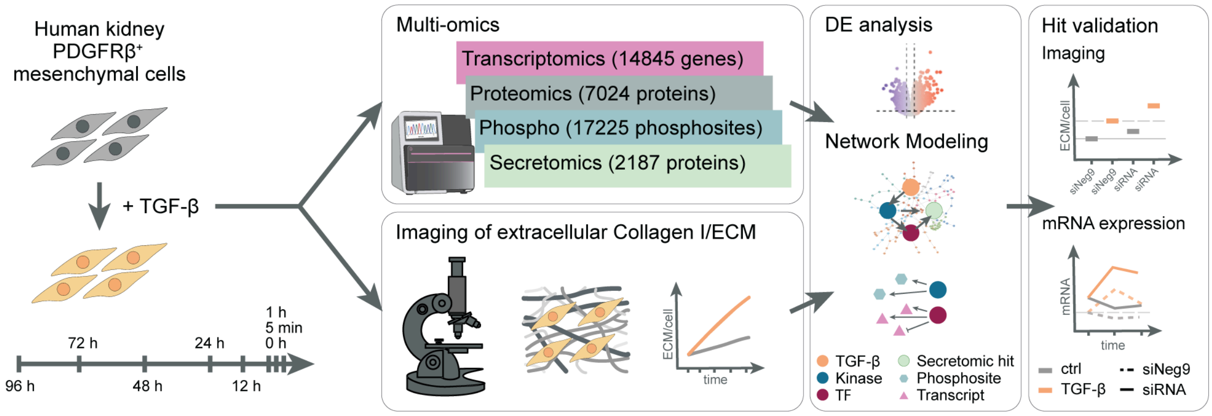

2.For every group in the multi-omics, what is the n value?

Answer 2.5

We thank the reviewer for this question. Transcriptomics and secretomics samples were measured in triplicates. For (phospho)proteomics four samples were measured. We added this information to the figure 1 legend, starting at **line 165** in the revised manuscript:

PCA scatter plots of PC1 and PC2 for the different omics modalities. For phosphoproteomics, PC2 and PC3 are depicted. The gray color scale shows control samples over time, the yellow to violet gradient resolves the time for the TGF-β-treated samples. The shape of the points shows the control samples compared to the samples treated with TGF-β. Transcriptomics and secretomics samples were measured in triplicates, while for (phospho)proteomics four samples were measured.

Revision Plan

Significance

The insights gained from this study not only advance our understanding of kidney fibrosis but also pave the way for the development of novel therapeutic strategies targeting this challenging condition. There is still much to be done, though. For instance, the author's screening of early-activated transcription factors, such as FLI1 and E2F1, which function as negative regulators of collagen deposition, requires additional confirmation.

Reviewer #3

Evidence, reproducibility and clarity

In this study the authors sought to identify novel mechanisms underlying the progression of kidney fibrosis, by activating myofibroblast formation of a human kidney fibroblast cell line with TGF-beta, and collecting a time-series data set of transcriptome, proteome, phosphoproteome and secretome. They then performed a number of computational analyses to identify the key pathways and regulators that were driving the TGF-beta mediated responses in the early and late time points. They further validated several candidates experimentally with siRNA knockdowns, confirming FLI1 and E2F1 as two primary suppressors for myofibroblast activation.

Major comments

While all the experiments and data collections appeared to be carried out carefully, all data essentially came from one human PDGFRβ+ cell line derived from a previous study. Can this cell line fully represent the fibroblast populations in human kidneys? I could not find much information such as donor age, sex, or clinical conditions of the donor. It is unclear how much the cell line has been passaged, what is the level of clonality or the level of replication-induced senescence. How can we ensure that the mechanisms identified from one single cell line are robust and generalizable, truly representative of common kidney fibroblast cells or fibroblasts in general? The amount of multi-omics data collection was quite impressive, and I don't think it is realistic to repeat all those data generation experiments across multiple cell lines. Nonetheless, I feel that it is important to selectively validate some of the key findings on additional cell lines. On a related note, myofibroblast activation can be different between male and female in vivo and in vitro (https://www.biorxiv.org/content/10.1101/2024.10.02.615251v1.abstract). Is any of the findings in this study sex specific?

Answer 3.1

Revision Plan

We thank the reviewer for acknowledging our comprehensive approach and valuable feedback. Recent studies by Kuppe et al. have demonstrated that all kidney myofibroblasts are derived from PDGFRβ^{+} cells (Kuppe et al. 2021). There is a small contribution of around 20% of PDGFRβ^{+}/α^{-} pericytes to the myofibroblast pool while 80% of myofibroblasts are being derived from PDGFRβ^{+}/α^{+} mesenchymal cells.

The detailed characterization of donor cells was recently published (Bouwens et al. 2025). We therefore extended our materials and method section to add this information as well as passage information. In short, the authors have isolated non activated PDGFRβ+ cells from human kidneys without fibrosis and demonstrated that they can be activated *in vitro* to become matrix producing myofibroblasts (Bouwens et al. 2025; Kuppe et al. 2021). These cells co-express PDGFRα and PDGFRβ and thus we believe that they resemble the mesenchymal origin of myofibroblasts. While the whole population of fibroblasts, pericytes and myofibroblasts is quite heterogeneous with various intermediate cell states we believe that the cell-line we are using as a non activated mesenchymal population still largely reflects the fibroblasts source that contributes to 80% of myofibroblasts in human kidneys.

However, we agree with the reviewer that one single cell line never represents the entire heterogeneity of human kidney mesenchymal cells. This is also not what we are intending to claim. We are using this line to characterize the *in vitro* response of mesenchymal cells to TGF-β with subsequent fibrosis since this is important for *ex vivo* models, compound testing and drug development. To the best of our knowledge currently no good human kidney mesenchymal cell lines exist and researchers have widely used mouse primary cells that often where contaminated or cell-lines such as T1/2 or 3T3 that are far away from human biology. Therefore we believe that the used cell-line represents a very unique and scalable tool to study kidney fibrosis *in vitro*. Interestingly, our finding related to affected E2F1 activity could be confirmed in a recent independent study even if the same cell line was used (Bouwens et al. 2025). Furthermore, we suggested a comparative computational analysis to a recently published lung-fibrosis study to address this point as a planned revision.

Regarding the sex-specific nature of our data, the data is primarily hypothesis-generating but allows reproducible results that may lead to novel targets or drugs for potential repurposing, which require further validation. So any identified targets or drugs will always have to be tested in well balanced mouse models with both genders or more advanced tissue culture models (please see our discussion section for more information). While the intriguing question of gender-specific differences merits investigation, it extends beyond the scope of the current study.

Adjustment in methods, starting at **line 660** in the revised manuscript:

Cell Lines and Reagents

Human kidney PDGFRβ^{+} mesenchymal cells, isolated from a 71 year old male patient with normal eGFR, were received from the Kramann lab (for further information please check (Bouwens et al. 2025; Kuppe et al. 2021)) and cultured in low glucose DMEM growth medium (Gibco 31885) supplemented with 5% FBS (Gibco A5256701). Cells were maintained at 37°C in a humidified incubator with 5% CO_2 and passaged approximately three times a week. All experiments were carried out between passage 33 and 36. Mycoplasma testing was routinely conducted, yielding negative results.

Adjusted Results section, starting at **line 96** of the revised manuscript:

In this study, we present a comprehensive investigation of kidney fibrosis using an in vitro model system that enables detailed phenotypic and molecular disease characterization and has previously been used in the context of kidney fibrosis research (Bouwens et al. 2025; Kuppe et al. 2021).

Further adjustments are detailed in answer 3.6

Adjusted discussion section, starting at **line 639** in the revised manuscript:

On the cell line and reflected cell types we wrote:

As this study focuses on characterising TGF-β induced ECM production in vitro, it cannot fully recapitulate the complex multicellular interactions present in the kidney. While the used cell culture system is based on cells that co-express PDGFRα and PDGFRβ and can resemble the mesenchymal origin of myofibroblasts, their full in vivo

heterogeneity cannot be reflected by a single cell line (Bouwens et al. 2025). Future studies could address this by incorporating co-culture systems, organoid models (Lassé et al. 2023; Piossek et al. 2022) or precision-cut kidney slices (Bigaeva et al. 2019, 2020; Poosti et al. 2015; Stribos et al. 2016) to better reflect the in vivo environment.

On the additional evidence on E2F1 importance we wrote, starting at **line 608**: *Further investigations into the molecular mechanism of E2F1-mediated regulation revealed a complex regulatory network involving E2F1 in both early and late responses. Initially, E2F1 functions downstream of RELA, while in the later phase, its activity appears to be downregulated through a TGFB1-PAX8 signaling axis (Chaves-Moreira et al. 2022; Li et al. 2011). This is also in line with earlier studies using these cells that show that E2F1 activity fluctuates post TGF-β treatment (Bouwens et al. 2025).*

Minor comments

Results section 2.1. Authors state "Specifically, we observed the activation of myofibroblast-specific gene expression as the fibrotic process progresses linking long-term patient data with in vitro data obtained over the course of hours". However, the transcriptomic data (Figure 1F) shows very low # of hits for these myofibroblast specific genes. Does this indicate that these cells are already in the myofibroblast state and that this is a model for TGFB stimulation of myofibroblasts? More clarification on this and what is being modeled (including starting and ending state of these cells) is needed.

Answer 3.2

We acknowledge that our experimental setup using PDGFRβ$^+$ cells cannot definitively assess transitions between distinct cellular states, as these cells share common gene expression patterns. We further added clarification that we observe a change towards high-ECM-producing myofibroblasts (Kuppe et al. 2021) that are the main source of ECM in CKD.

Starting at **line 139** of the revised manuscript:

Specifically, we observed the increase of myofibroblast-specific protein expression as the

fibrotic process progresses linking long-term patient data with in vitro data obtained over the course of hours. Together with the observed increased ECM accumulation this indicates the activation of high-ECM-producing myofibroblasts (Kuppe et al. 2021) that are the main source of ECM in CKD.

The authors tend to overstate how this in vitro model reflects complex disease phenotypes. The main issue is what is being modeled, which appears to be mostly TGF-B induced ECM production and possibly enhanced myofibroblast state signatures?

Answer 3.3

We agree with the reviewer and corrected our writing in the results sections and further elaborated on this in the discussion.

Starting at **line 639** in the revised manuscript:

As this study focuses on characterising TGF-β induced ECM production in vitro, it cannot fully recapitulate the complex multicellular interactions present in the kidney. While the used cell culture system is based on cells that co-express PDGFRα and PDGFRβ and can resemble the mesenchymal origin of myofibroblasts, their full in vivo heterogeneity cannot be reflected by a single cell line (Bouwens et al. 2025). Future studies could address this by incorporating co-culture systems, organoid models (Lassé et al. 2023; Piossek et al. 2022) or precision-cut kidney slices (Bigaeva et al. 2019, 2020; Poosti et al. 2015; Stribos et al. 2016) to better reflect the in vivo environment.

On page 23: "To summarize, the integration of multi-omic data into time-resolved network models of early and late fibrotic responses revealed dynamic shifts in signaling pathways, transcription factor activities, and protein interactions, highlighting the temporal complexity of kidney fibrosis progression and identifying both well-known and novel regulatory factors for further investigation." Here it is not clear that the timeline used in this paper is recapitulating "late fibrotic processes" seen in vivo nor how it truly relates to kidney fibrosis progression.

Answer 3.4

We agree with the reviewer and adapted this part to, starting at **line 390**:

To summarize, the integration of multi-omic data into time-resolved network models of early and late in vitro processes revealed dynamic shifts in signaling pathways, transcription factor activities, and protein interactions, highlighting the temporal complexity of ECM production in kidney fibroblasts upon TGF-β stimulation and identifying both well-known and novel regulatory factors for further investigation.

Also section 2.4: "To further validate the role of these transcription factors in the development of fibrotic diseases...". This is not something that this in vitro model can achieve.

Answer 3.5

We appreciate this important point raised by the reviewer and therefore adjusted following part of the manuscript starting at **line 435**:

To further validate the role of these transcription factors in the observed ECM accumulation, we exploited the perturbability of the used in vitro model system.

In section 2.4, the paragraph discussing E2F1 is poorly written, over uses the word "activity", and is not clear.

Answer 3.6

We agree with the reviewer and adapted following part of the revised manuscript starting at **line 482**:

A second very interesting case is the transcription factor E2F1, which shows increased activity at earlier time points and then appears to be deactivated over time (Figure 3F). This is also in line with earlier studies using these cells that show that E2F1 activity is enhanced at 24 h but decreased at 72 h post TGF-β treatment (Bouwens et al. 2025). Upon E2F1 knockdown, increased deposition of ECM is observed in imaging data, as with most other early activated TFs (Figure 4B).
In the early network model, E2F1 activation is predicted to occur downstream of RELA. (Figure 3E). The temporal downregulation of E2F1 is included in the late network model

downstream of TGFB1 and PAX8 (Figure 4F), consistent with the estimated PAX8 activity, which shows a strong downregulation over time (Figure 4G).
Moreover, E2F1 regulation is associated with secretory processes, as shown by the late network model, which suggests that SERPINE1 (also known as plasminogen activator inhibitor-1 PAI-1) and SERPINE2 secretion, key modulators of collagen deposition, are upregulated downstream of E2F1 inhibition (Figure 4F, Figure S6E). SERPINE1 and SERPINE2 do not cause this effect by influencing COL1 expression, but by inhibiting collagen degradation and thus contributing to increased extracellular matrix deposition (Bergheim et al. 2006; Qi et al. 2008). This effect is confirmed at the SERPINE1 mRNA level, which increases considerably upon E2F1 knockdown (Figure 4H, Figure S6F, G). Furthermore, the RT-qPCR data supports the hypothesis that E2F1 has an indirect effect on collagen deposition, as the expression of COL1A1 mRNA is only moderately affected by E2F1 knockdown, in contrast to the observations for FLI1 (Figure 4H, Figure S6F, G).

Figure 3E: it is a bit of surprise to see HDAC1 being a node there connecting RELA to KLF4/FLI1. HDAC1 deacetylates histones and many transcription factors, hence the effects are likely to be very broad. Can the authors explain why it has such a high specificity in this context?

Answer 3.7

We agree with the reviewer that HDAC1 is an enzyme involved in the regulation of many proteins and probably does not affect only two proteins specifically. This observation can be attributed to the way in which the computational network model is derived. We optimize to include as many proteins as possible with a signal (e.g. altered activity in TFs) while keeping the network size as small as possible. This means that there could be many more HDAC-regulated proteins in this dataset that were either not considered as input for the model or that are downstream of another protein (not all potential edges are returned by the algorithm used).

We included this information in the discussion section starting at **line 629**:

While our study provides valuable insights, it also has limitations. Despite the power of

our integrative approach, there are still aspects that we do not fully understand, such as the precise mechanisms causing the downregulation of the described transcription factors. Additionally, our network model provides valuable insights and potential downstream mechanisms, but these need to be thoroughly validated. In contrast, there could also be a variety of interesting potential mechanisms reflected in the data that are missing in the computational network model because it optimises for a balance of size and signal and therefore contains incomplete parts.

Significance

Overall, this is a nice study with several strengths. The time-series multi-omics data along the course of myofibroblast activation generated in this study is very impressive. While transcriptomic data collection is quite routine, the proteomics, phosphoproteomics, and secretomics data really lifted the significance of this study to another level. As demonstrated in their study, these data allowed the authors to carry out much more sophisticated computational analyses (which is another major strengths of this study), examining the responses in terms of gene regulation, protein production, modification, secretion at the early and late stages of fibrotic activation, formulating a mechanistic model. This study managed to get much closer to determining causal and direct regulation, compared with many other previous studies staying at the level of correlation and enrichments. Finally, some of the key regulators identified in their analyses were validated experimentally by siRNA knockdowns.

Bibliography

Arif M, Basu A, Wolf KM, Park JK, Pommerolle L, et al. 2023. An integrative multiomics framework for identification of therapeutic targets in pulmonary fibrosis. *Adv Sci (Weinh)*. e2207454

Badia-I-Mompel P, Vélez Santiago J, Braunger J, Geiss C, Dimitrov D, et al. 2022. decoupleR: ensemble of computational methods to infer biological activities from omics data. *Bioinformatics Advances*. 2(1):vbac016

Bergheim I, Guo L, Davis MA, Duveau I, Arteel GE. 2006. Critical role of plasminogen activator inhibitor-1 in cholestatic liver injury and fibrosis. *J. Pharmacol. Exp. Ther.* 316(2):592–600

Bhuva D, Smyth G, Garnham A. 2024. *Msigdb: An ExperimentHub Package for the Molecular Signatures Database (MSigDB)*. BIoconductor

Bigaeva E, Gore E, Simon E, Zwick M, Oldenburger A, et al. 2019. Transcriptomic characterization of culture-associated changes in murine and human precision-cut tissue slices. *Arch. Toxicol.* 93(12):3549–83

Bigaeva E, Stribos EGD, Mutsaers HAM, Piersma B, Leliveld AM, et al. 2020. Inhibition of tyrosine kinase receptor signaling attenuates fibrogenesis in an ex vivo model of human renal fibrosis. *Am. J. Physiol. Renal Physiol.* 318(1):F117–34

Bouwens D, Kabgani N, Bergerbit C, Kim H, Ziegler S, et al. 2025. A bioprinted and scalable model of human tubulo-interstitial kidney fibrosis. *Biomaterials*. 316:123009

Chaves-Moreira D, Mitchell MA, Arruza C, Rawat P, Sidoli S, et al. 2022. The transcription factor PAX8 promotes angiogenesis in ovarian cancer through interaction with SOX17. *Sci. Signal.* 15(728):eabm2496

Chen CZC, Peng YX, Wang ZB, Fish PV, Kaar JL, et al. 2009. The Scar-in-a-Jar: studying potential antifibrotic compounds from the epigenetic to extracellular level in a single well. *Br. J. Pharmacol.* 158(5):1196–1209

Cisek K, Krochmal M, Klein J, Mischak H. 2016. The application of multi-omics and systems biology to identify therapeutic targets in chronic kidney disease. *Nephrol. Dial. Transplant.* 31(12):2003–11

Coentro JQ, May U, Prince S, Zwaagstra J, Ritvos O, et al. 2021. Adapting the Scar-in-a-Jar to Skin Fibrosis and Screening Traditional and Contemporary Anti-Fibrotic Therapies. *Front. Bioeng. Biotechnol.* 9:756399

D'Souza RCJ, Knittle AM, Nagaraj N, van Dinther M, Choudhary C, et al. 2014. Time-resolved dissection of early phosphoproteome and ensuing proteome changes in response to TGF-β. *Sci. Signal.* 7(335):rs5

Dugourd A, Kuppe C, Sciacovelli M, Gjerga E, Gabor A, et al. 2021. Causal integration of multi-omics data with prior knowledge to generate mechanistic hypotheses. *Mol. Syst. Biol.* 17(1):e9730

Dugourd A, Saez-Rodriguez J. 2019. Footprint-based functional analysis of multiomic data. *Current Opinion in Systems Biology*. 15:82–90

Eddy S, Mariani LH, Kretzler M. 2020. Integrated multi-omics approaches to improve classification of chronic kidney disease. *Nat. Rev. Nephrol.* 16(11):657–68

Friedman SL, Sheppard D, Duffield JS, Violette S. 2013. Therapy for fibrotic diseases: nearing the starting line. *Sci. Transl. Med.* 5(167):167sr1

Garrido-Rodriguez M, Potel C, Burtscher ML, Becher I, Rodriguez-Mier P, et al. 2024. Evaluating signaling pathway inference from kinase-substrate interactions and phosphoproteomics data. *BioRxiv*

Ghosh AK, Vaughan DE. 2012. PAI-1 in tissue fibrosis. *J. Cell. Physiol.* 227(2):493–507

Huang S, Chen B, Humeres C, Alex L, Hanna A, Frangogiannis NG. 2020. The role of Smad2 and Smad3 in regulating homeostatic functions of fibroblasts in vitro and in adult mice. *Biochim. Biophys. Acta Mol. Cell Res.* 1867(7):118703

Karsdal MA, Nielsen SH, Leeming DJ, Langholm LL, Nielsen MJ, et al. 2017. The good and the bad collagens of fibrosis - Their role in signaling and organ function. *Adv. Drug Deliv. Rev.* 121:43–56

Khan MM, Galea G, Jung J, Zukowska J, Lauer D, et al. 2024. Dextromethorphan inhibits collagen and collagen-like cargo secretion to ameliorate lung fibrosis. *Sci. Transl. Med.* 16(778):eadj3087

Kuppe C, Ibrahim MM, Kranz J, Zhang X, Ziegler S, et al. 2021. Decoding myofibroblast origins in human kidney fibrosis. *Nature*. 589(7841):281–86

Lake BB, Menon R, Winfree S, Hu Q, Melo Ferreira R, et al. 2023. An atlas of healthy and injured cell states and niches in the human kidney. Nature. 619(7970):585–94

Lassé M, El Saghir J, Berthier CC, Eddy S, Fischer M, et al. 2023. An integrated organoid omics map extends modeling potential of kidney disease. *Nat. Commun.* 14(1):4903

Lichtman MK, Otero-Vinas M, Falanga V. 2016. Transforming growth factor beta (TGF-β) isoforms in wound healing and fibrosis. *Wound Repair Regen.* 24(2):215–22

Li CG, Nyman JE, Braithwaite AW, Eccles MR. 2011. PAX8 promotes tumor cell growth by transcriptionally regulating E2F1 and stabilizing RB protein. *Oncogene*. 30(48):4824–34

Meng X-M, Nikolic-Paterson DJ, Lan HY. 2016. TGF-β: the master regulator of fibrosis. *Nat. Rev. Nephrol.* 12(6):325–38

Meng XM, Huang XR, Chung ACK, Qin W, Shao X, et al. 2010. Smad2 protects against TGF-beta/Smad3-mediated renal fibrosis. *J. Am. Soc. Nephrol.* 21(9):1477–87

Mikhailova EV, Romanova IV, Bagrov AY, Agalakova NI. 2023. Fli1 and tissue fibrosis in various diseases. *Int. J. Mol. Sci.* 24(3):

Piossek F, Beneke S, Schlichenmaier N, Mucic G, Drewitz S, Dietrich DR. 2022. Physiological oxygen and co-culture with human fibroblasts facilitate in vivo-like properties in human renal proximal tubular epithelial cells. *Chem. Biol. Interact.* 361:109959

Poosti F, Pham BT, Oosterhuis D, Poelstra K, van Goor H, et al. 2015. Precision-cut kidney slices (PCKS) to study development of renal fibrosis and efficacy of drug targeting ex vivo. *Dis. Model. Mech.* 8(10):1227–36

Qi L, Higgins SP, Lu Q, Samarakoon R, Wilkins-Port CE, et al. 2008. SERPINE1 (PAI-1) is a prominent member of the early G0 --> G1 transition "wound repair" transcriptome in p53 mutant human keratinocytes. *J. Invest. Dermatol.* 128(3):749–53

Rasmussen DGK, Boesby L, Nielsen SH, Tepel M, Birot S, et al. 2019. Collagen turnover profiles in chronic kidney disease. *Sci. Rep.* 9(1):16062

Reznichenko A, Nair V, Eddy S, Tomilo M, Slidel T, et al. 2021. Molecular stratification of chronic kidney disease. *medRxiv*

Rønnow SR, Dabbagh RQ, Genovese F, Nanthakumar CB, Barrett VJ, et al. 2020. Prolonged Scar-in-a-Jar: an in vitro screening tool for anti-fibrotic therapies using biomarkers of extracellular matrix synthesis. *Respir. Res.* 21(1):108

Samarakoon R, Higgins SP, Higgins CE, Higgins PJ. 2008. TGF-beta1-induced plasminogen activator inhibitor-1 expression in vascular smooth muscle cells requires pp60(c-src)/EGFR(Y845) and Rho/ROCK signaling. *J. Mol. Cell. Cardiol.* 44(3):527–38

Stribos EGD, Luangmonkong T, Leliveld AM, de Jong IJ, van Son WJ, et al. 2016. Precision-cut human kidney slices as a model to elucidate the process of renal fibrosis. *Transl. Res.* 170:8-16.e1

Szalai B, Saez-Rodriguez J. 2020. Why do pathway methods work better than they should? *FEBS Lett.* 594(24):4189–4200

Türei D, Korcsmáros T, Saez-Rodriguez J. 2016. OmniPath: guidelines and gateway for literature-curated signaling pathway resources. *Nat. Methods.* 13(12):966–67

Türei D, Valdeolivas A, Gul L, Palacio‐Escat N, Klein M, et al. 2021. Integrated intra‐ and intercellular signaling knowledge for multicellular omics analysis. *Mol. Syst. Biol.* 17(3):

Zhao M, Wang L, Wang M, Zhou S, Lu Y, et al. 2022. Targeting fibrosis, mechanisms and cilinical trials. *Signal Transduct. Target. Ther.* 7(1):206

Zhou S, Yin X, Mayr M, Noor M, Hylands PJ, Xu Q. 2020. Proteomic landscape of TGF-β1-induced fibrogenesis in renal fibroblasts. *Sci. Rep.* 10(1):19054

Zi Z, Chapnick DA, Liu X. 2012. Dynamics of TGF-β/Smad signaling. *FEBS Lett.* 586(14):1921–28

5th Feb 2025

Manuscript Number: MSB-2025-12898-T
Title: Dynamic multi-omics and mechanistic modeling approach uncovers novel mechanisms of kidney fibrosis progression

Dear Dr. Pepperkok,

Thank you again for submitting your work to Molecular Systems Biology. We have now discussed your manuscript, the reviews from Review Commons, and your revisions. According to your revision plan to address the remaining reviewer comments with the additional validation of your findings to ensure that the results are not limited to a single cell line, we would send your paper back to the original reviewers who reviewed your paper at Review Commons for re-review at Molecular Systems Biology (given resubmission in a reasonable timeframe). Please let me know in case you would like to discuss in further detail, I would be happy to schedule a call.

When submitting your revised manuscript, please carefully review the instructions that follow below. We perform an initial quality control of all revised manuscripts before re-review; failure to include requested items will delay the evaluation of your revision.

We require:
1) A .docx formatted version of the manuscript text (including legends for main figures, EV figures and tables). Please make sure that the changes are highlighted to be clearly visible. Alternatively you may choose to submit your manuscript as a LaTeX file.

2) Individual production quality figure files as .eps, .tif, .jpg (one file per figure). For guidance, download the 'Figure Guide PDF' (https://www.embopress.org/page/journal/17574684/authorguide#figureformat).

3) At EMBO Press we ask authors to provide source data for the main figures. Our source data coordinator will contact you to discuss which figure panels we would need source data for and will also provide you with helpful tips on how to upload and organize the files.

4) A .docx formatted letter INCLUDING the reviewers' reports and your detailed point-by-point responses to their comments. As part of the EMBO Press transparent editorial process, the point-by-point response is part of the Peer Review File (PRF), which will be published alongside your paper.

5) A complete author checklist, which you can download from our author guidelines (https://www.embopress.org/page/journal/17574684/authorguide#submissionofrevisions). Please insert information in the checklist that is also reflected in the manuscript. The completed author checklist will also be part of the PRF.

6) Please note that all corresponding authors are required to supply an ORCID ID for their name upon submission of a revised manuscript.

7) It is mandatory to include a 'Data Availability' section after the Materials and Methods. Before submitting your revision, primary datasets produced in this study need to be deposited in an appropriate public database, and the accession numbers and database listed under 'Data Availability'. Please remember to provide a reviewer password if the datasets are not yet public (see https://www.embopress.org/page/journal/17574684/authorguide#dataavailability).

In case you have no data that requires deposition in a public database, please state so in this section as follows: "This study includes no data deposited in external repositories". Note that the Data Availability Section is restricted to new primary data that are part of this study.

8) All Materials and Methods need to be described in the main text using our 'Structured Methods' format, which is required for all research articles. According to this format, the Methods section includes a Reagents and Tools Table (listing key reagents, experimental models, software and relevant equipment and including their sources and relevant identifiers) followed by a Methods and Protocols section describing the methods using a step-by-step protocol format. The aim is to facilitate adoption of the methodologies across labs. Please upload the Reagents and Tools table as a separate document when submitting your revised manuscript. More information on how to adhere to this format as well as a downloadable template (.docx) for the Reagents and Tools Table can be found in our author guidelines:
https://www.embopress.org/page/journal/17444292/authorguide#structuredmethods

An example of a Method paper with Structured Methods can be found here:
https://www.embopress.org/doi/10.15252/msb.20178071.

9) For data quantification: please specify the name of the statistical test used to generate error bars and p-values, the number

(n) of independent experiments (specify technical or biological replicates) underlying each data point and the test used to calculate p-values in each figure legend. The figure legends should contain a basic description of n, p-values and the test applied. Graphs must include a description of the bars and the error bars (s.d., s.e.m.). Please provide exact p-values (in either the figure or figure legend).

10) Our journal encourages inclusion of *data citations in the reference list* to directly cite datasets that were re-used and obtained from public databases. Data citations in the article text are distinct from normal bibliographical citations and should directly link to the database records from which the data can be accessed. In the main text, data citations are formatted as follows: "Data ref: Smith et al, 2001" or "Data ref: NCBI Sequence Read Archive PRJNA342805, 2017". In the Reference list, data citations must be labeled with "[DATASET]". A data reference must provide the database name, accession number/identifiers and a resolvable link to the landing page from which the data can be accessed at the end of the reference. Further instructions are available at .

11) We replaced Supplementary Information with Expanded View (EV) Figures and Tables that are collapsible/expandable online. EV Figures should be cited as 'Figure EV1, Figure EV2" etc... in the text and their respective legends should be included in the main text after the legends of regular figures.
- For the figures that you do NOT wish to display as Expanded View figures, they should be bundled together with their legends in a single PDF file called *Appendix*, which should start with a short Table of Content. Appendix figures should be referred to in the main text as: "Appendix Figure S1, Appendix Figure S2" etc.
- Additional Tables/Datasets should be labeled and referred to as Table EV1, Dataset EV1, etc. Legends should be provided in a separate tab in case of .xls files. Alternatively, the legend can be supplied as a separate text file (README) and zipped together with the Table/Dataset file.
See detailed instructions here:
https://www.embopress.org/page/journal/17574684/authorguide#expandedview

12) Author contributions: CRediT has replaced the traditional author contributions section because it offers a systematic machine-readable author contributions format that allows for more effective research assessment. Please remove the Authors Contributions from the manuscript and use the free text boxes beneath each contributing author's name in our system to add specific details on the author's contribution. More information is available in our guide to authors.

13) Disclosure statement and competing interests: We updated our journal's competing interests policy in January 2022 and request authors to consider both actual and perceived competing interests. Please review the policy https://www.embopress.org/competing-interests and update your competing interests if necessary.

14) Every published paper now includes a 'Synopsis' to further enhance discoverability. Synopses are displayed on the journal webpage and are freely accessible to all readers. They include a short stand first (maximum of 300 characters, including space) as well as 2-5 one-sentences bullet points that summarizes the paper. Please write the bullet points to summarize the key NEW findings. They should be designed to be complementary to the abstract - i.e. not repeat the same text. We encourage inclusion of key acronyms and quantitative information (maximum of 30 words / bullet point). Please use the passive voice. Please attach these in a separate file or send them by email, we will incorporate them accordingly.

Please also suggest a striking image or visual abstract to illustrate your article as a PNG file 550 px wide x 300-600 px high.

Please note that these would be the final versions and changes during proofing are usually not allowed.

15) As part of the EMBO Publications transparent editorial process initiative (see our policy here: https://www.embopress.org/transparent-process#Review_Process), Molecular Systems Biology will publish online a Peer Review File (PRF) to accompany accepted manuscripts.
In the event of acceptance, this file will be published in conjunction with your paper and will include the anonymous referee reports, your point-by-point response and all pertinent correspondence relating to the manuscript. Let us know whether you agree with the publication of the PRF and as here, if you want to remove or not any figures from it prior to publication.
Please note that the Author checklist will be published at the end of the PRF.

Molecular Systems Biology has a "scooping protection" policy, whereby similar findings that are published by others during review or revision are not a criterion for rejection. Should you decide to submit a revised version, I do ask that you get in touch after three months if you have not completed it, to update us on the status.

I look forward to receiving your revised manuscript.

Yours sincerely,

Poonam Bheda

Poonam Bheda, PhD
Scientific Editor
Molecular Systems Biology

***
Rev_Com_number: RC-2024-02741
New_manu_number: MSB-2025-12898-T
Corr_author: Pepperkok
Title: Dynamic multi-omics and mechanistic modeling approach uncovers novel mechanisms of kidney fibrosis progression

Point-by-point response

Please note that we included an additional affiliation for one of the authors.

MMK: Translational Lung Research Center Heidelberg (TLRC), German Center for Lung Research (DZL), 69120 Heidelberg, Germany

We replaced Khan et al. 2023 with Khan et al. 2024.

Reviewer #1

Summary

This study showed measurements and integration of time-series multiple omics data of the human kidney PDGFR beta+ cells responding to TGF-beta stimuli. The authors also presented key pathways that were inferred based on estimating activities of TFs and kinases, and confirmed by knockdown experiments whose phenotypes can be observed by means of imaging.

The omics data were well measured under appropriate quality controls. Hence, this study will attract interests from specialists of kidney fibrosis and systems biologists. But there still remains concerns regarding arguments and data presentation of the manuscript.

Major concerns

1. The content of Discussion is too thin. Particularly, it is uncommon to see a discussion section with no citations like this manuscript. Cite related studies and compare with the own results so that the authors can argue originality and novelty of this work. I also see some citations in Results. Usually it is opposite: little citations in Results section and many citations in Discussions.

Answer 1.1

We agree with the reviewer's observation that the style of citation in the manuscript can be improved and has to be adapted. We made several adjustment in the results and discussion sections:

Here we refer to the discussion section of the revised manuscript, starting at **line 563.**

2. Put more emphasis on presenting biological relevances in order for readers to easily recognize them. I guess that Figs. 4C and 4F are examples of such biological findings.

Answer 1.2

We agree with the reviewer that we can emphasize the biological findings more and highlighted them further in the discussion as described in answer 1.1.. At the same time, we want to be careful with proposed biological mechanisms, to avoid overinterpretation of the obtained results. We considered this in our adapted version of the discussion.

3. Draw the whole picture(s) of the integrated networks, not only subnetworks. If too much complicated, the complexity itself will be important information for readers.

Answer 1.3

We thank the reviewer for this observation. The networks are included in Figure 3A and provided as Supplementary Table which can be explored in Cytoscape. We made adjustments in the Figure legend and the text to highlight this better.

Starting at **line 1483** in the revised manuscript**:**

(A) Schematic representation of the chosen computational integration strategy. Two network models were created to reflect the early (network on the left) and late (right network) response to TGF-β stimulation integrating transcriptomics, phosphoproteomics and secretomics data for different time points.

4. On SMAD2:

4a) The responses of p-SMAD2 in Fig. S2 are remarkably different in the two batches. The authors should discuss the reason of these outcomes. Which of the two batches exhibited similar responses to the phosphoproteome data?

Answer 1.4a

We agree with the reviewer that the quantification of the two western blots is quite different. However, this experiment has been performed as qualitative quality control to confirm that canonical TGF-β signaling has been induced through the stimulation and to facilitate defining important time points for the multi-omics experiment. Unfortunately, the SMAD2/3 transcription factors are expressed at a rather low level, wherefore they were not detected in the phosphoproteomics experiments. To do so, a much deeper coverage would be required. We added this information to the results section as shown below.

Starting at **line 226** in the revised manuscript:

While most of these transcription factors show a constitutive up/down regulation over time, there are examples such as FLI1 with a temporally regulated activity that only increases after

one hour of stimulation (Figure 2D). Note that this analysis allows assessment of TF activity, which can be difficult with direct measurement, as we for example were unable to detect activating SMAD2/3 phosphorylation in the phosphoproteome experiment, due to limited coverage of low-abundant proteins.

4b) What possible reasons do authors think about that SMAD2/3 are not included in the transcriptional regulatory networks presented in Figs. 3 and 4 in spite of their importance in the TGFbeta signaling? Should be argued.

Answer 1.4b

SMAD2/3 are established central modulators in TGF-β signaling and an important part of the canonical pathway. The used network inference algorithm however optimises the combination of measured nodes with a PKN that is agnostic to pathway level information. This is the reason why topologies are often found that do not correspond to our textbook understanding of the underlying process. We tried to address this by fixing the SMAD molecules downstream of TGF-β as described in the Method section 4.5 Data analysis, Network modeling. As a result, SMAD2, SMAD1 and SMAD4 are modeled downstream of TGF-β as shown in Figure S5. Exactly which successive edges are modeled then depends on a variety of reasons, e.g. the effect size of the observed signal, the prior knowledge coverage of the modeled nodes and the combination with all other input nodes. Therefore, the resulting networks must not be interpreted as a pathway visualisation but rather as an analysis result which can be used to explore and validate potential connections between different data modalities. We added a clarification in the result section of the manuscript.

Starting at **line 270** in the revised manuscript:

This provides a mechanistic molecular hypothesis for the observed ECM deposition at later time points, thus reflecting the dynamic nature of cellular communication (Figure 3A). As underlying PKN we used a directed and signed protein-protein interaction network retrieved from Omnipath (Türei et al. 2016, 2021). It should be noted that this modeling approach provides molecular associations that are not guided by commonly assumed pathway topologies and cannot be considered as a visualization of canonical pathways. This is in line with recent findings showing that the definition of so-called canonical pathways can be biased by the way biochemical research is done and is not necessarily useful to reflect signaling processes (Garrido-Rodriguez et al. 2024).

4c) What molecular mechanism can cause the increase in SERPINE1 expression dependent on TGFbeta? The mechanism may involve SMAD2/3 but neither presented nor argued. Should be clarified.

Answer 1.4c

We thank the reviewer for this question. Previous studies have linked the expression changes of SERPINE1 to MAPK as well as SMAD signaling (Ghosh & Vaughan 2012; Samarakoon et al. 2008). As described in Figure 4, our data as well as the multi-omics integration analysis suggest that the increase in SERPINE1 expression is related to the inactivation of the E2F1 transcription factor. We could show that siRNA mediated E2F1 knockdown induced SERPINE1 mRNA expression. The model suggests further regulation via the transcription factor PAX8 which can be activated by TGF-β. However, this has not been validated further as it is out of scope for this study. We clarified this finding in the revised Discussion section of the manuscript, see Answer 1.1.

4d) It seems inconsistent that knockdown of the early-activated TFs cause extensive ECM accumulation in the knockdown experiment presented in Fig. 4B. Did the authors see suppression of ECM accumulation by knockdown of SMAD2/3? Should be presented.

Answer 1.4d

We thank the reviewer for this question. We did not focus on knockdown of SMAD2/3 as the canonical pathway of TGF-β is well-studied (D'Souza et al. 2014; Friedman et al. 2013; Huang et al. 2020; Meng et al. 2010, 2016; Zhao et al. 2022; Zi et al. 2012). As shown by others, knockdown of SMAD2 actually increases COL1 expression while knockdown of SMAD3 decreases it (e.g. Meng et. al 2010). This confirms the central role of SMAD2/3 in TGFB signaling, but also highlights the complexity of transcriptional reprogramming occurring during fibrogenesis. We directed our validation efforts on potential novel regulators that could play a role in kidney fibrogenesis besides the characterised canonical pathway.
We believe our assay allows us to make the presented conclusions, as (i) all knockdowns have been confirmed by qPCR, (ii) the knockdown of COL1A1 does reduce ECM accumulation as expected and (iii) for FLI1 and E2F1 we confirmed our results with a second siRNA. Further, one of the tested TFs (HNF4G) does reduce collagen deposition.

Minor concerns

1. Fig. 1D: Numbers in the Venn diagram of 'proteomics technologies' do not match with the numbers in another Venn diagram on the right hand side. Should be corrected or explained.

Answer 1.5

The reason for this is that a protein can be detected by multiple of the used proteomics technologies, but we only used the unique set of measured proteins to compare to the transcriptomics dataset.

2. Fig. 2B: 'INFalpha' should be IFNalpha, so is 'INFgamma'.

Answer 1.6

We thank the reviewer for this observation and have adapted it.

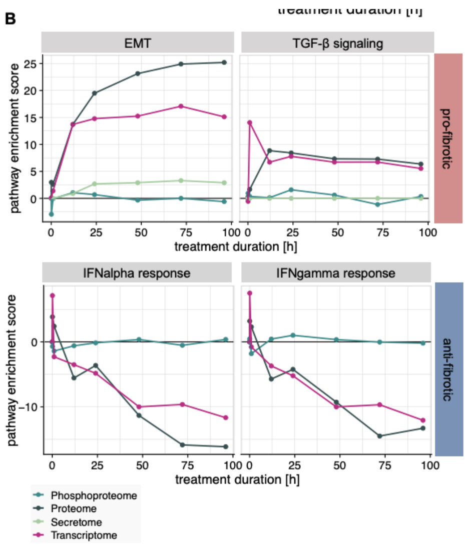

3. Fig. 2B, Fig. S4C: What does the sign of 'Pathway enrichment score' mean? How is it calculated? Should be explained.

Answer 1.7

We thank the reviewer for this question. The pathway enrichment analysis has been performed as described in the method section “pathway enrichment analysis” with the DecoupleR package (Badia-I-Mompel et al. 2022). This algorithm estimates an enrichment score which corresponds to the mean signal or an alternative summary statistic of all annotated pathway members. We extended the description in the Method section accordingly.

Starting at **line 952** in the revised manuscript:

For the path enrichment analysis, MSIGDB (Bhuva et al. 2024) Hallmark pathways were used with the decoupleR package (Badia-I-Mompel et al. 2022) (normalized weighted

average method) to calculate pathway enrichment scores from log2 fold change values. This algorithm estimates an enrichment score which corresponds to the mean signal or an alternative summary statistic of all annotated pathway members.

4. Do not fit curves to data that should be drawn in line graphs (e.g. Figs. 3F, 4E, 4G etc.).

Answer 1.8

We thank the reviewer for this question, but we do think a fit is appropriate to visualise time course data representing fold-changes or activity scores as it facilitates interpretation of the curve.

We extended our description in the figure legends, for example in **line 521** of the revised manuscript:

RT-qPCR data to confirm FLI1 knockdown effect on its potential downstream target COL1A1 +/-TGF-β stimulation at different time points. A temporal profile has been fitted using a loess fit.

5. How did the authors plot the regression curves presented in Fig. 4D? Should be clarified.

Answer 1.9

We thank the reviewer for this observation and have adapted our text accordingly for all plots with fits.

Starting at **line 1533** in the revised manuscript:
RT-qPCR data to confirm FLI1 knockdown effect on its potential downstream target COL1A1 +/-TGF-β stimulation at different time points. A temporal profile has been fitted using a loess fit.

6. What is 'PKN'? Maybe 'Prior Knowledge Network', but clearly spelled out when it first appears.

Answer 1.10

We thank the reviewer for this observation and have adapted our text accordingly.

Starting at **line 260** in the revised manuscript:

We next integrated the findings obtained from the differential expression and kinase and TF activity analyzes in a network model, using a modified version of COSMOS, an optimisation method that identifies putative causal paths explaining changes in enzymes with altered

activity and multi-omics measurements based on a causal Prior Knowledge Network (PKN) (Dugourd et al. 2021).

7. Did the PNK-nodes in the networks exhibit quantitative changes in any of the omics data?

Answer 1.11

We thank the reviewer for this comment. In total we have 8 PKN proteins which exhibit significant changes in abundance in at least one time point. One example is JUNB, a PKN node modelled to be active in the late network which also goes up in the RNA-seq data. It is not in the TFs considered significant, which could be due to many reasons we cannot assess e.g. data coverage or an activation in a temporal dynamic we did not cover with the chosen experimental design. All data related to this is accessible in the Tables EV1 and EV5. Of note, we found many more changes at the activity level, illustrating the value of measuring different omics and deriving from them the activity; indeed activity of a protein is not necessarily reflected by its abundance (Szalai & Saez-Rodriguez 2020). This is the reason the network-model integration has been performed on activity not abundance level.

8 What do the axes of the heatmaps mean in Fig. S3A? Why are there more categories than total sample numbers? Should be clarified.

Answer 1.12

The reviewer raises an important point about Figure S3A. Each axis represents individual biological replicates by experimental condition (treatment and time point), and shown per omics modality. We have adjusted the figure legend to provide better clarity.

Staring at **line 1405** in the revised manuscript:

Heatmap showing Pearson correlation coefficients between individual biological replicates, calculated using TMT reporter intensities (proteomics/phosphoproteomics) or gene counts (transcriptomics). Each row/column represents a single biological replicate.

Reviewer #2

Summary

The authors presented a comprehensive, time-resolved multi-omics analysis of kidney fibrosis using an in vitro model system based on human kidney PDGFRβ+ mesenchymal cells aimed at unraveling disease mechanisms. This research advanced our understanding of the pathogenesis of kidney fibrosis. However, this reviewer has several concerns.

Major comments

1.Why does the 0.08h group not exist in Fig S1? What's more, the detection of ECM appears to be insufficient as it only reveals COL1 expression.

Answer 2.1

We thank the reviewer for this comment. We did not include the 0.08 h as well as the 1 h group in the imaging based experiments. The reason for this was that we did not expect significant changes in ECM deposition at these time points. COL1 synthesis and deposition takes time as it undergoes hydroxylation, glycosylation and cleavage by peptidases before being deposited (Karsdal et al. 2017; Khan et al. 2024). In fact, our data show that there was a significant increase of COL1 deposition after 24 h of TGF-β treatment compared to the corresponding control which was not observed at 12 h, the earliest time point tested (Figure 1).

Imaging of deposited COL1, as a major component of the fibrotic extracellular matrix (Karsdal et al. 2017; Lichtman et al. 2016), has been used in previous studies, such as (Khan et al. 2024), to successfully screen for anti-fibrotic drugs. Hence we used this assay as a phenotypic assay supporting our data generated using multi-omics and to identify potential regulators of kidney fibrosis.

To clarify, we stated in the Materials and Methods section, that our initial experiments were performed with an anti-COL1 antibody, while we used a fluorescent dye binding to fibrotic ECM as new batches of the anti-COL1 did not work:

Starting at **line 564** in the revised manuscript:

For extracellular matrix (ECM) visualization, cells were incubated with anti-COL1 antibody (Rockland 600-401-103-0.5, 1:500 in PBS) for 1-1.5 hours at room temperature, washed, and then incubated with fluorescently labeled secondary anti-rabbit IgG AlexaFluor 488 (Molecular Probes A11008, 1:400 in PBS) in PBS for 30-45 minutes. Washed cells were

kept in PBS and imaged. Due to issues with new batches of the anti-COL1 antibody, GFP-labeled CNA35 dye (EMBL protein expression facility, 1:250 in PBS) was used for validation experiments (siRNA knockdowns of TFs). After fixation and washing, cells were incubated with CNA35 for 1-1.5 hours, washed, and imaged. In cases of increased autofluorescence from siRNA transfection, cells were stained with an anti-GFP (Origene TP401) followed by Alexa 647-conjugated secondary anti-rabbit (Invitrogen A21245).

2.Fig S2A shows that p-smad2 has 11 bands, whereas Smad2 has 12 bands. Moreover, the repeatability of the two repeated trials is not very excellent. Additionally, why not look at the phosphoproteomics data to see how p-smad2 changes?

Answer 2.2

We appreciate the reviewer's feedback. The additional band in the SMAD2 blot is due to well spillover, while the 12th band represents a 72-hour siSMAD2 control sample used to validate antibody specificity. We adjusted the Figure EV2 accordingly (see below).

While we acknowledge the quantitative differences between the replicate Western blots, these experiments served as qualitative quality controls to confirm canonical TGF-β pathway activation and guide time point selection for our multi-omics analysis. SMAD2/3 were not detected in our phosphoproteomics data due to their low abundance, which would require substantially deeper phosphoproteome coverage for reliable detection. Please also refer to Answer 1.4a

Starting at **line 1392** in the revised manuscript:

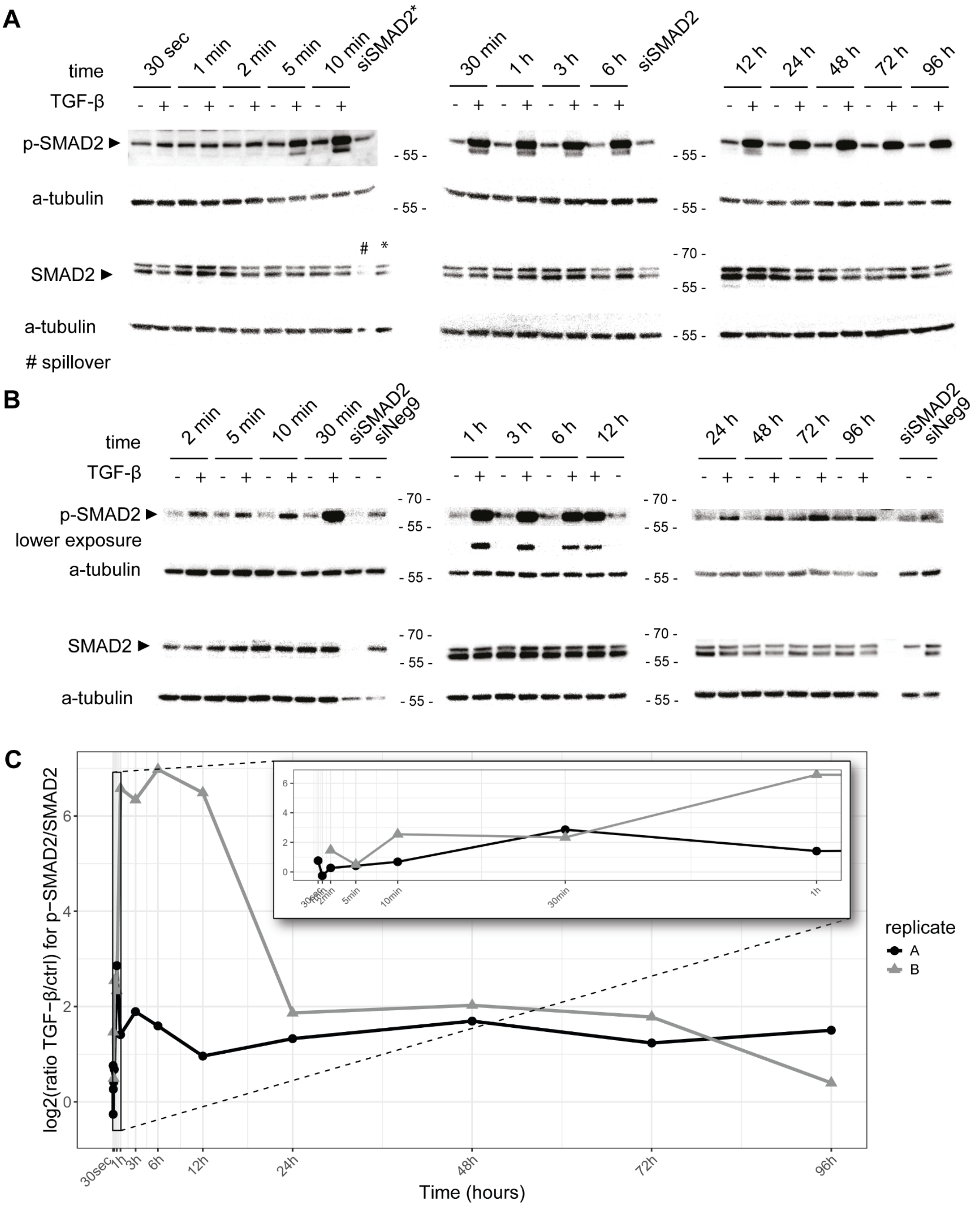

Figure EV2

(A) Western blot analysis of phosphorylated SMAD2 (p-SMAD2), total SMAD2, and α-tubulin (loading control) in response to TGF-β treatment over time. Time points range from 30 seconds to 96 hours. siSMAD2 condition is included as a control. A spill-over has been labelled with '#', the correct corresponding band and position has been labeled with ''.*

3. The early-activated transcription factors screened by the author, including FLI1 and E2F1, act as negative regulators of collagen deposition, needs further verification.

Answer 2.3

We agree with the reviewer that further verification of FLI1 and E2F1 is needed to prove their role as negative regulators in the context of kidney fibrosis. However, an in-depth verification is out of the scope of this study in which we wanted to suggest potential new regulators of fibrosis at a system-wide scale using an integrated analysis approach.

In terms of the robustness and generalisability of our complete dataset, we performed an additional comparative analysis to lung fibrosis datasets and included an additional Figure (Figure EV4) and text. Please see Answer 3.1 for the complete description of this.

We also reinforced the fact that specific suggested mechanisms require additional validation in the discussion section.

Line 461:

While our study provides valuable insights, it also has limitations. Despite the power of our integrative approach, there are still aspects that we do not fully understand, such as the precise mechanisms causing the downregulation of the described transcription factors. Additionally, our network model provides valuable insights and potential downstream mechanisms, but these need to be thoroughly validated.

Line 492:

The presence of both shared and distinct transcriptional regulators highlights fundamental fibrotic pathways that operate across organs and supports the generalizability of the presented data to study fibrosis related processes. As expected, given the differences in the organ systems, the experimental setups used and the technical variability of the methods applied in our study, the spearman correlation values did not exceed a low to moderate range as reported in other studies (Blank et al. 2020; Morrow et al. 2019). It will be important though to validate and complement our study in the future in systems which are closer to the organ level in animal models or patient tissues. .

Minor comments

1.The graphical abstract and the abstract don't agree on how many time points there are-is it seven or eight?

Answer 2.4

We thank the reviewer for the observation. The time points included in this study are 0, 0.08, 1, 12, 24, 48, 72, and 96 hours (8 time points). In Fig 1, there are only 7 lines but 8 time points mentioned, which is due to the fact that 0 and 0.08 h are so close together that we showed it as one line but aimed to illustrate all time points by mentioning them in the graph.

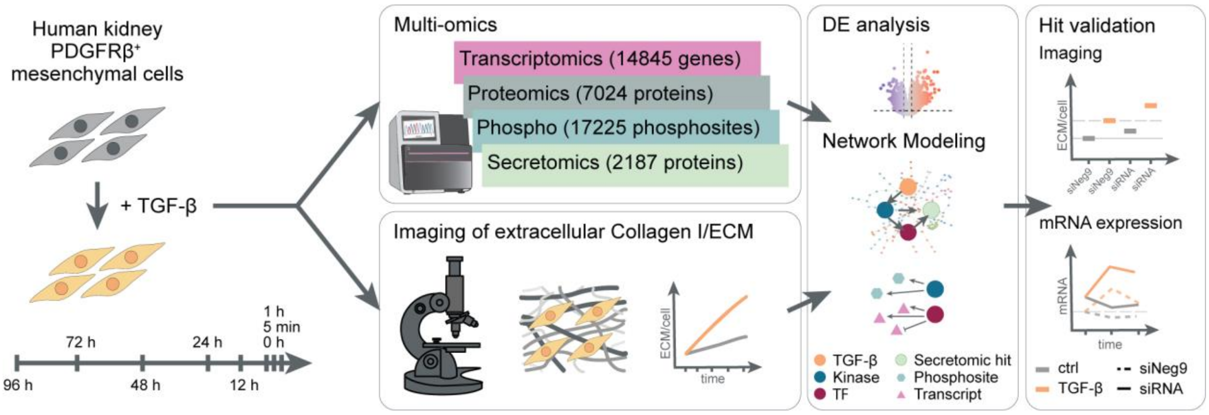

2.For every group in the multi-omics, what is the n value?

<u>Answer 2.5</u>

We thank the reviewer for this question. Transcriptomics and secretomics samples were measured in triplicates. For (phospho)proteomics four samples were measured. We added this information to the figure 1 legend, starting at **line 1362** in the revised manuscript:

PCA scatter plots of PC1 and PC2 for the different omics modalities. For phosphoproteomics, PC2 and PC3 are depicted. The gray color scale shows control samples over time, the yellow to violet gradient resolves the time for the TGF-β-treated samples. The shape of the points shows the control samples compared to the samples treated with TGF-β. Transcriptomics and secretomics samples were measured in triplicates, while for (phospho)proteomics four samples were measured.

Significance

The insights gained from this study not only advance our understanding of kidney fibrosis but also pave the way for the development of novel therapeutic strategies targeting this challenging condition. There is still much to be done, though. For instance, the author's screening of early-activated transcription factors, such as FLI1 and E2F1, which function as negative regulators of collagen deposition, requires additional confirmation.

Reviewer #3

Evidence, reproducibility and clarity

In this study the authors sought to identify novel mechanisms underlying the progression of kidney fibrosis, by activating myofibroblast formation of a human kidney fibroblast cell line with TGF-beta, and collecting a time-series data set of transcriptome, proteome, phosphoproteome and secretome. They then performed a number of computational analyses to identify the key pathways and regulators that were driving the TGF-beta mediated responses in the early and late time points. They further validated several candidates experimentally with siRNA knockdowns, confirming FLI1 and E2F1 as two primary suppressors for myofibroblast activation.

Major comments

While all the experiments and data collections appeared to be carried out carefully, all data essentially came from one human PDGFRβ+ cell line derived from a previous study. Can this cell line fully represent the fibroblast populations in human kidneys? I could not find much information such as donor age, sex, or clinical conditions of the donor. It is unclear how much the cell line has been passaged, what is the level of clonality or the level of replication-induced senescence. How can we ensure that the mechanisms identified from one single cell line are robust and generalizable, truly representative of common kidney fibroblast cells or fibroblasts in general? The amount of multi-omics data collection was quite impressive, and I don't think it is realistic to repeat all those data generation experiments across multiple cell lines. Nonetheless, I feel that it is important to selectively validate some of the key findings on additional cell lines. On a related note, myofibroblast activation can be different between male and female in vivo and in vitro (https://www.biorxiv.org/content/10.1101/2024.10.02.615251v1.abstract). Is any of the findings in this study sex specific?

Answer 3.1

We thank the reviewer for acknowledging our comprehensive approach and valuable feedback. Recent studies by Kuppe et al. have demonstrated that all kidney myofibroblasts are derived from PDGFRβ^+ cells (Kuppe et al. 2021). There is a small contribution of around 20% of PDGFRβ^+/α^- pericytes to the myofibroblast pool while 80% of myofibroblasts are being derived from PDGFRβ^+/α^+ mesenchymal cells.

The detailed characterization of donor cells was recently published (Bouwens et al. 2025). We therefore extended our materials and method section to add this information as well as

passage information. In short, the authors have isolated non activated PDGFRβ+ cells from human kidneys without fibrosis and demonstrated that they can be activated *in vitro* to become matrix producing myofibroblasts (Bouwens et al. 2025; Kuppe et al. 2021). These cells co-express PDGFRα and PDGFRβ and thus we believe that they resemble the mesenchymal origin of myofibroblasts. While the whole population of fibroblasts, pericytes and myofibroblasts is quite heterogeneous with various intermediate cell states we believe that the cell-line we are using as a non activated mesenchymal population still largely reflects the fibroblasts source that contributes to 80% of myofibroblasts in human kidneys. However, we agree with the reviewer that one single cell line never represents the entire heterogeneity of human kidney mesenchymal cells. This is also not what we are intending to claim. We are using this cell line to characterize the *in vitro* response of mesenchymal cells to TGF-β with subsequent fibrosis since this is important for *ex vivo* models, compound testing and drug development. To the best of our knowledge currently no good human kidney mesenchymal cell lines exist and researchers have widely used mouse primary cells that often were contaminated or cell-lines such as T1/2 or 3T3 that are far away from human biology. Therefore, we believe that the used cell-line represents a very unique and scalable tool to study kidney fibrosis *in vitro.* To address the reviewer's comment, we performed a comparative computational analysis to two recently published lung-fibrosis studies (Khan et al. 2024; Khan et al. 2021) and expanded the manuscript with an additional figure(Figure EV4) and text. While we are fully aware that this does not replace a comprehensive experimental validation we do hope that this strengthens our dataset to address the reviewers concerns within the scope of this study.

Regarding the sex-specific nature of our data, the data is primarily hypothesis-generating but allows reproducible results that may lead to novel targets or drugs for potential repurposing, which require further validation. So any identified targets or drugs will always have to be tested in well balanced mouse models with both genders or more advanced tissue culture models (please see our discussion section for more information). While the intriguing question of gender-specific differences merits investigation, it extends beyond the scope of the current study.

Adjustment in methods, starting at **line 518** in the revised manuscript:

Cell Lines and Reagents

Human kidney PDGFRβ$^+$ mesenchymal cells, isolated from a 71 year old male patient with normal eGFR, were received from the Kramann lab (for further information please check (Bouwens et al. 2025; Kuppe et al. 2021)) and cultured in low glucose DMEM growth medium (Gibco 31885) supplemented with 5% FBS (Gibco A5256701). Cells were

maintained at 37°C in a humidified incubator with 5% CO_2 and passaged approximately three times a week. All experiments were carried out between passage 33 and 36. Mycoplasma testing was routinely conducted, yielding negative results.

Adjusted Results section, starting at **line 105** of the revised manuscript:

In this study, we present a comprehensive investigation of kidney fibrosis using an in vitro model system that enables detailed phenotypic and molecular disease characterization and has previously been used in the context of kidney fibrosis research (Bouwens et al. 2025; Kuppe et al. 2021).

Further adjustments are detailed in answer 3.6

Adjusted discussion section, starting at **line 474** in the revised manuscript:

On the cell line and reflected cell types we wrote:
As this study focuses on characterising TGF-β induced ECM production in vitro, it cannot fully recapitulate the complex multicellular interactions present in the kidney. While the used cell culture system is based on cells that co-express PDGFRα and PDGFRβ and can resemble the mesenchymal origin of myofibroblasts, their full in vivo heterogeneity cannot be reflected by a single cell line (Bouwens et al. 2025). Future studies could address this by incorporating co-culture systems, organoid models (Lassé et al. 2023; Piossek et al. 2022) or precision-cut kidney slices (Bigaeva et al. 2019, 2020; Poosti et al. 2015; Stribos et al. 2016) to better reflect the in vivo environment.

On the additional evidence on E2F1 importance we wrote, starting at **line 608**:
Further investigations into the molecular mechanism of E2F1-mediated regulation revealed a complex regulatory network involving E2F1 in both early and late responses. Initially, E2F1 functions downstream of RELA, while in the later phase, its activity appears to be downregulated through a TGFB1-PAX8 signaling axis (Chaves-Moreira et al. 2022; Li et al. 2011). This is also in line with earlier studies using these cells that show that E2F1 activity fluctuates post TGF-β treatment (Bouwens et al. 2025).

Additional computational analysis
On the robustness and generalizability of our observations we included an additional figure and text on the comparison to lung fibrosis data.

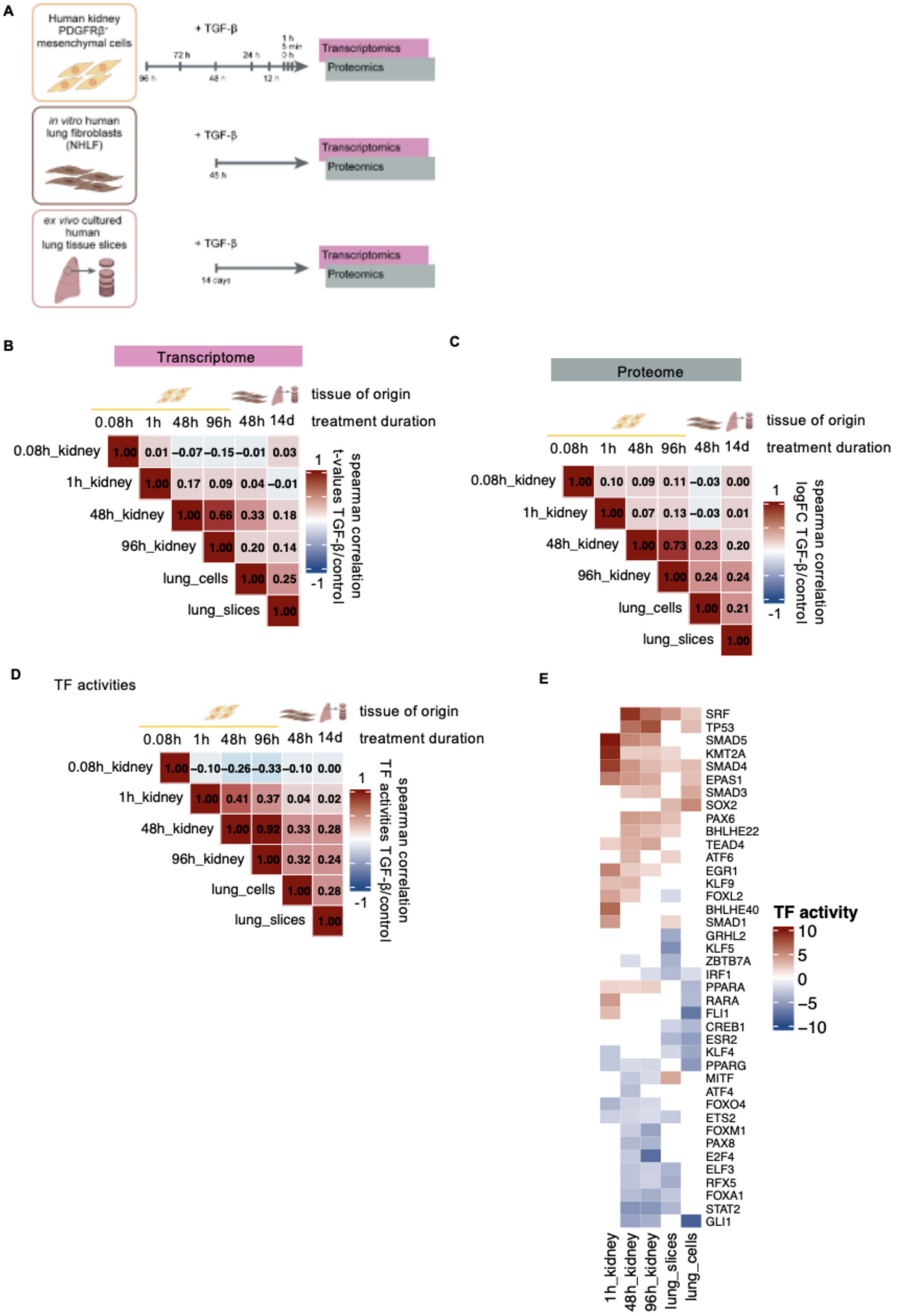
A
Human kidney PDGFRβ+ mesenchymal cells
+ TGF-β
Transcriptomics
Proteomics
in vitro human lung fibroblasts (NHLF)
ex vivo cultured human lung tissue slices
B
Transcriptome
tissue of origin
treatment duration
0.08h 1h 48h 96h 48h 14d
0.08h_kidney
1h_kidney
48h_kidney
96h_kidney
lung_cells
lung_slices
spearman correlation t-values TGF-β/control
C
Proteome
spearman correlation logFC TGF-β/control
D
TF activities
spearman correlation TF activities TGF-β/control
E
TF activity
SRF
TP53
SMAD5
KMT2A
SMAD4
EPAS1
SMAD3
SOX2
PAX6
BHLHE22
TEAD4
ATF6
EGR1
KLF9
FOXL2
BHLHE40
SMAD1
GRHL2
KLF5
ZBTB7A
IRF1
PPARA
RARA
FLI1
CREB1
ESR2
KLF4
PPARG
MITF
ATF4
FOXO4
ETS2
FOXM1
PAX8
E2F4
ELF3
RFX5
FOXA1
STAT2
GLI1
1h_kidney
48h_kidney
96h_kidney
lung_slices
lung_cells

Figure EV 4

(A) Overview of different studies and datasets included into the comparison analysis. The transcriptomics and proteomics data generated in this study were compared to omics data from TGF-β-treated normal human lung fibroblasts (NHLF) (Khan et al. 2024) and human lung tissue slices (Khan et al. 2021).

(B) Spearman correlation of all transcript t-values (DEseq2 or limma, t-test) comparing TGF-β treatment and the DMSO or untreated control for the samples of the different studies. Note: DMSO as control was only used in the lung related studies.

(C) Spearman correlation of all protein log2 fold-changes comparing TGF-β treatment and the DMSO control (limma, t-test) for the samples of the different studie.

(D) Spearman correlation of transcription factor activities affected upon TGF-β treatment or the samples of the different studies (enzyme activity enrichment analysis using decoupleR, normalised mean method, p-value < 0.1 in at least one condition).

(E) Top hits of transcription factor activities affected upon TGF-β treatment of the samples of the different studies (enzyme activity enrichment analysis using decoupleR, normalised mean method, p-value < 0.1, top 10 TFs per sample).

Additional Text in the results section starting at line 158

We further compared our findings with multi-omics data from two recent lung fibrosis studies (Khan et al. 2021, 2024) to determine cell line and organ specificity. These datasets included: (i) transcriptomics and proteomics from human lung fibroblasts treated with TGF-β for 48 hours, and (ii) similar data from ex vivo cultured human lung tissue slices treated with TGF-β for 14 days (Figure EV4A). We compared the results of the differential analysis for the transcriptomics and proteomics data for both datasets with two early and late time points of our human kidney cell dataset. We observed a correlation of the changes in the lung datasets with the late time points of TGF-β stimulation in the kidney cells to a similar extent as between the two lung datasets (Figure EV4B, Figure EV4C). Interestingly, all early processes of the TGF-β response in kidney cells represented in the 5 min and 1 hour time points appeared to be exclusive and absent in the lung data for both proteome and transcriptome (Figure EV4B, Figure EV4C).

Additional text in the results section starting at line 242

To better understand the robustness and generalizability of the obtained results, an additional estimation of TF activity was performed using two previously introduced lung fibrosis datasets (Khan et al. 2021, 2024)(Figure EV4). Similar to the results of the differential analysis (Figure EV4B, C), the predicted TF activities after TGF-β stimulation in

lung cells and tissue slices showed a correlation with late time points of kidney cell stimulation, while all early activation processes could not be detected with the later readout times (Figure EV4D). Of note, several of the TFs correlating between kidney and lung cells are known canonical TGF-β mediators (for example, SMAD3/4, SRF and GLI1; Figure EV4E).

Extended discussion content follows line 483

To contextualize our findings within the broader landscape of fibrotic disease mechanisms, we compared our kidney cell data with multi-omics datasets from human lung fibroblasts and ex vivo lung tissue slices treated with TGF-β (Khan et al. 2021, 2024)*). We detected a common response to TGF-β stimulation across cell culture and organ systems in terms of differential expression and TF activity levels for longer treatment durations (Figure EV4). The early TF activation processes that we further investigated in the kidney cell culture system are not present in the lung datasets. This is likely due to the fact that these early time points were uniquely studied in our work here, and emphasizes the power of longitudinal approaches in multi-omic studies. The presence of both shared and distinct transcriptional regulators highlights fundamental fibrotic pathways that operate across organs and supports the generalisability of the presented data to study fibrosis related processes. As expected, given the differences in the organ systems, the experimental setups used and the technical variability of the methods applied in our study, the spearman correlation values did not exceed a low to moderate range as reported in other studies* (Blank et al. 2020; Morrow et al. 2019)*. It will be important though to validate and complement our study in the future in systems which are closer to the organ level in animal models or patient tissues.*

Minor comments

Results section 2.1. Authors state "Specifically, we observed the activation of myofibroblast-specific gene expression as the fibrotic process progresses linking long-term patient data with in vitro data obtained over the course of hours". However, the transcriptomic data (Figure 1F) shows very low # of hits for these myofibroblast specific genes. Does this indicate that these cells are already in the myofibroblast state and that this is a model for TGFB stimulation of myofibroblasts? More clarification on this and what is being modeled (including starting and ending state of these cells) is needed.

Answer 3.2

We acknowledge that our experimental setup using PDGFRβ$^+$ cells cannot definitively assess transitions between distinct cellular states, as these cells share common gene

expression patterns. We further added clarification that we observe a change towards high-ECM-producing myofibroblasts (Kuppe et al. 2021) that are the main source of ECM in CKD.

Starting at **line 152** of the revised manuscript:

Specifically, we observed the increase of myofibroblast-specific protein expression as the fibrotic process progresses linking long-term patient data with in vitro data obtained over the course of hours. Together with the observed increased ECM accumulation this indicates the activation of high-ECM-producing myofibroblasts (Kuppe et al. 2021) that are the main source of ECM in CKD.

The authors tend to overstate how this in vitro model reflects complex disease phenotypes. The main issue is what is being modeled, which appears to be mostly TGF-B induced ECM production and possibly enhanced myofibroblast state signatures?

Answer 3.3

We agree with the reviewer and corrected our writing in the results sections and further elaborated on this in the discussion.

Starting at **line 474** in the revised manuscript:

As this study focuses on characterising TGF-β induced ECM production in vitro, it cannot fully recapitulate the complex multicellular interactions present in the kidney. While the used cell culture system is based on cells that co-express PDGFRα and PDGFRβ and can resemble the mesenchymal origin of myofibroblasts, their full in vivo heterogeneity cannot be reflected by a single cell line (Bouwens et al. 2025). Future studies could address this by incorporating co-culture systems, organoid models (Lassé et al. 2023; Piossek et al. 2022) or precision-cut kidney slices (Bigaeva et al. 2019, 2020; Poosti et al. 2015; Stribos et al. 2016) to better reflect the in vivo environment.

On page 23: "To summarize, the integration of multi-omic data into time-resolved network models of early and late fibrotic responses revealed dynamic shifts in signaling pathways, transcription factor activities, and protein interactions, highlighting the temporal complexity of kidney fibrosis progression and identifying both well-known and novel regulatory factors for further investigation." Here it is not clear that the timeline used in this paper is recapitulating "late fibrotic processes" seen in vivo nor how it truly relates to kidney fibrosis progression.

Answer 3.4

We agree with the reviewer and adapted this part to, starting at **line 318**:

To summarize, the integration of multi-omic data into time-resolved network models of early and late in vitro processes revealed dynamic shifts in signaling pathways, transcription factor activities, and protein interactions, highlighting the temporal complexity of ECM production in kidney fibroblasts upon TGF-β stimulation and identifying both well-known and novel regulatory factors for further investigation.

Also section 2.4: "To further validate the role of these transcription factors in the development of fibrotic diseases...". This is not something that this in vitro model can achieve.

Answer 3.5

We appreciate this important point raised by the reviewer and therefore adjusted following part of the manuscript starting at **line 326**:

To further validate the role of these transcription factors in the observed ECM accumulation, we exploited the perturbability of the used in vitro model system.

In section 2.4, the paragraph discussing E2F1 is poorly written, over uses the word "activity", and is not clear.

Answer 3.6

We agree with the reviewer and adapted following part of the revised manuscript starting at **line 365**:

A second interesting case is the transcription factor E2F1, which shows increased activity at earlier time points and then appears to be deactivated over time (Figure 3F). This is also in line with earlier studies using these cells that show that E2F1 activity is enhanced at 24 h but decreased at 72 h post TGF-β treatment (Bouwens et al. 2025). Upon E2F1 knockdown, increased deposition of ECM is observed in imaging data, as with most other early activated TFs (Figure 4B). In the early network model, E2F1 activation is predicted to occur downstream of RELA. (Figure 3E). The temporal downregulation of E2F1 is included in the late network model downstream of TGFB1 and PAX8 (Figure 4F), consistent with the estimated PAX8 activity, which shows a strong downregulation over time (Figure 4G).
Moreover, E2F1 regulation is associated with secretory processes, as shown by the late network model, which suggests that SERPINE1 (also known as plasminogen activator inhibitor-1 PAI-1) and SERPINE2 secretion, key modulators of collagen deposition, are upregulated downstream of E2F1 inhibition (Figure 4F, Figure S6E). SERPINE1 and SERPINE2 do not cause this effect by influencing COL1 expression, but by inhibiting collagen degradation and thus contributing to increased extracellular matrix deposition

(Bergheim et al. 2006; Qi et al. 2008). This effect is confirmed at the SERPINE1 mRNA level, which increases considerably upon E2F1 knockdown (Figure 4H, Figure S6F, G). Furthermore, the RT-qPCR data supports the hypothesis that E2F1 has an indirect effect on collagen deposition, as the expression of COL1A1 mRNA is only moderately affected by E2F1 knockdown, in contrast to the observations for FLI1 (Figure 4H, Figure S6F, G).

Figure 3E: it is a bit of surprise to see HDAC1 being a node there connecting RELA to KLF4/FLI1. HDAC1 deacetylates histones and many transcription factors, hence the effects are likely to be very broad. Can the authors explain why it has such a high specificity in this context?

Answer 3.7

We agree with the reviewer that HDAC1 is an enzyme involved in the regulation of many proteins and probably does not affect only two proteins specifically. This observation can be attributed to the way in which the computational network model is derived. We optimize to include as many proteins as possible with a signal (e.g. altered activity in TFs) while keeping the network size as small as possible. This means that there could be many more HDAC-regulated proteins in this dataset that were either not considered as input for the model or that are downstream of another protein (not all potential edges are returned by the algorithm used).

We included this information in the discussion section starting at **line 629**:

While our study provides valuable insights, it also has limitations. Despite the power of our integrative approach, there are still aspects that we do not fully understand, such as the precise mechanisms causing the downregulation of the described transcription factors. Additionally, our network model provides valuable insights and potential downstream mechanisms, but these need to be thoroughly validated. In contrast, there could also be a variety of interesting potential mechanisms reflected in the data that are missing in the computational network model because it optimises for a balance of size and signal and therefore contains incomplete parts.

Significance

Overall, this is a nice study with several strengths. The time-series multi-omics data along the course of myofibroblast activation generated in this study is very impressive. While transcriptomic data collection is quite routine, the proteomics, phosphoproteomics, and secretomics data really lifted the significance of this study to another level. As

demonstrated in their study, these data allowed the authors to carry out much more sophisticated computational analyses (which is another major strengths of this study), examining the responses in terms of gene regulation, protein production, modification, secretion at the early and late stages of fibrotic activation, formulating a mechanistic model. This study managed to get much closer to determining causal and direct regulation, compared with many other previous studies staying at the level of correlation and enrichments. Finally, some of the key regulators identified in their analyses were validated experimentally by siRNA knockdowns.

Bibliography

Arif M, Basu A, Wolf KM, Park JK, Pommerolle L, et al. 2023. An integrative multiomics framework for identification of therapeutic targets in pulmonary fibrosis. *Adv Sci (Weinh)*. e2207454

Badia-I-Mompel P, Vélez Santiago J, Braunger J, Geiss C, Dimitrov D, et al. 2022. decoupleR: ensemble of computational methods to infer biological activities from omics data. *Bioinformatics Advances*. 2(1):vbac016

Bergheim I, Guo L, Davis MA, Duveau I, Arteel GE. 2006. Critical role of plasminogen activator inhibitor-1 in cholestatic liver injury and fibrosis. *J. Pharmacol. Exp. Ther.* 316(2):592–600

Bhuva D, Smyth G, Garnham A. 2024. *Msigdb: An ExperimentHub Package for the Molecular Signatures Database (MSigDB)*. BIoconductor

Bigaeva E, Gore E, Simon E, Zwick M, Oldenburger A, et al. 2019. Transcriptomic characterization of culture-associated changes in murine and human precision-cut tissue slices. *Arch. Toxicol.* 93(12):3549–83

Bigaeva E, Stribos EGD, Mutsaers HAM, Piersma B, Leliveld AM, et al. 2020. Inhibition of tyrosine kinase receptor signaling attenuates fibrogenesis in an ex vivo model of human renal fibrosis. *Am. J. Physiol. Renal Physiol.* 318(1):F117–34

Bouwens D, Kabgani N, Bergerbit C, Kim H, Ziegler S, et al. 2025. A bioprinted and scalable model of human tubulo-interstitial kidney fibrosis. *Biomaterials*. 316:123009

Chaves-Moreira D, Mitchell MA, Arruza C, Rawat P, Sidoli S, et al. 2022. The transcription factor PAX8 promotes angiogenesis in ovarian cancer through interaction with SOX17. *Sci. Signal.* 15(728):eabm2496

Chen CZC, Peng YX, Wang ZB, Fish PV, Kaar JL, et al. 2009. The Scar-in-a-Jar: studying potential antifibrotic compounds from the epigenetic to extracellular level in a single well. *Br. J. Pharmacol.* 158(5):1196–1209

Cisek K, Krochmal M, Klein J, Mischak H. 2016. The application of multi-omics and systems biology to identify therapeutic targets in chronic kidney disease. *Nephrol. Dial. Transplant.* 31(12):2003–11

Coentro JQ, May U, Prince S, Zwaagstra J, Ritvos O, et al. 2021. Adapting the Scar-in-a-Jar to Skin Fibrosis and Screening Traditional and Contemporary Anti-Fibrotic Therapies. *Front. Bioeng. Biotechnol.* 9:756399

D'Souza RCJ, Knittle AM, Nagaraj N, van Dinther M, Choudhary C, et al. 2014. Time-resolved dissection of early phosphoproteome and ensuing proteome changes in response to TGF-β. *Sci. Signal.* 7(335):rs5

Dugourd A, Kuppe C, Sciacovelli M, Gjerga E, Gabor A, et al. 2021. Causal integration of multi-omics data with prior knowledge to generate mechanistic hypotheses. *Mol. Syst. Biol.* 17(1):e9730

Dugourd A, Saez-Rodriguez J. 2019. Footprint-based functional analysis of multiomic data. *Current Opinion in Systems Biology*. 15:82–90

Eddy S, Mariani LH, Kretzler M. 2020. Integrated multi-omics approaches to improve classification of chronic kidney disease. *Nat. Rev. Nephrol.* 16(11):657–68

Friedman SL, Sheppard D, Duffield JS, Violette S. 2013. Therapy for fibrotic diseases: nearing the starting line. *Sci. Transl. Med.* 5(167):167sr1

Garrido-Rodriguez M, Potel C, Burtscher ML, Becher I, Rodriguez-Mier P, et al. 2024. Evaluating signaling pathway inference from kinase-substrate interactions and phosphoproteomics data. *BioRxiv*

Ghosh AK, Vaughan DE. 2012. PAI-1 in tissue fibrosis. *J. Cell. Physiol.* 227(2):493–507
Huang S, Chen B, Humeres C, Alex L, Hanna A, Frangogiannis NG. 2020. The role of Smad2 and Smad3 in regulating homeostatic functions of fibroblasts in vitro and in adult mice. *Biochim. Biophys. Acta Mol. Cell Res.* 1867(7):118703
Karsdal MA, Nielsen SH, Leeming DJ, Langholm LL, Nielsen MJ, et al. 2017. The good and the bad collagens of fibrosis - Their role in signaling and organ function. *Adv. Drug Deliv. Rev.* 121:43–56
Khan MM, Galea G, Jung J, Zukowska J, Lauer D, et al. 2024. Dextromethorphan inhibits collagen and collagen-like cargo secretion to ameliorate lung fibrosis. *Sci. Transl. Med.* 16(778):eadj3087
Kuppe C, Ibrahim MM, Kranz J, Zhang X, Ziegler S, et al. 2021. Decoding myofibroblast origins in human kidney fibrosis. *Nature*. 589(7841):281–86
Lake BB, Menon R, Winfree S, Hu Q, Melo Ferreira R, et al. 2023. An atlas of healthy and injured cell states and niches in the human kidney. Nature. 619(7970):585–94
Lassé M, El Saghir J, Berthier CC, Eddy S, Fischer M, et al. 2023. An integrated organoid omics map extends modeling potential of kidney disease. *Nat. Commun.* 14(1):4903
Lichtman MK, Otero-Vinas M, Falanga V. 2016. Transforming growth factor beta (TGF-β) isoforms in wound healing and fibrosis. *Wound Repair Regen.* 24(2):215–22
Li CG, Nyman JE, Braithwaite AW, Eccles MR. 2011. PAX8 promotes tumor cell growth by transcriptionally regulating E2F1 and stabilizing RB protein. *Oncogene*. 30(48):4824–34
Meng X-M, Nikolic-Paterson DJ, Lan HY. 2016. TGF-β: the master regulator of fibrosis. *Nat. Rev. Nephrol.* 12(6):325–38
Meng XM, Huang XR, Chung ACK, Qin W, Shao X, et al. 2010. Smad2 protects against TGF-beta/Smad3-mediated renal fibrosis. *J. Am. Soc. Nephrol.* 21(9):1477–87
Mikhailova EV, Romanova IV, Bagrov AY, Agalakova NI. 2023. Fli1 and tissue fibrosis in various diseases. *Int. J. Mol. Sci.* 24(3):
Piossek F, Beneke S, Schlichenmaier N, Mucic G, Drewitz S, Dietrich DR. 2022. Physiological oxygen and co-culture with human fibroblasts facilitate in vivo-like properties in human renal proximal tubular epithelial cells. *Chem. Biol. Interact.* 361:109959
Poosti F, Pham BT, Oosterhuis D, Poelstra K, van Goor H, et al. 2015. Precision-cut kidney slices (PCKS) to study development of renal fibrosis and efficacy of drug targeting ex vivo. *Dis. Model. Mech.* 8(10):1227–36
Qi L, Higgins SP, Lu Q, Samarakoon R, Wilkins-Port CE, et al. 2008. SERPINE1 (PAI-1) is a prominent member of the early G0 --> G1 transition “wound repair” transcriptome in p53 mutant human keratinocytes. *J. Invest. Dermatol.* 128(3):749–53
Rasmussen DGK, Boesby L, Nielsen SH, Tepel M, Birot S, et al. 2019. Collagen turnover profiles in chronic kidney disease. *Sci. Rep.* 9(1):16062
Reznichenko A, Nair V, Eddy S, Tomilo M, Slidel T, et al. 2021. Molecular stratification of chronic kidney disease. *medRxiv*
Rønnow SR, Dabbagh RQ, Genovese F, Nanthakumar CB, Barrett VJ, et al. 2020. Prolonged Scar-in-a-Jar: an in vitro screening tool for anti-fibrotic therapies using biomarkers of extracellular matrix synthesis. *Respir. Res.* 21(1):108
Samarakoon R, Higgins SP, Higgins CE, Higgins PJ. 2008. TGF-beta1-induced plasminogen activator inhibitor-1 expression in vascular smooth muscle cells requires pp60(c-src)/EGFR(Y845) and Rho/ROCK signaling. *J. Mol. Cell. Cardiol.* 44(3):527–38
Stribos EGD, Luangmonkong T, Leliveld AM, de Jong IJ, van Son WJ, et al. 2016. Precision-cut human kidney slices as a model to elucidate the process of renal fibrosis. *Transl.*

Res. 170:8-16.e1

Szalai B, Saez-Rodriguez J. 2020. Why do pathway methods work better than they should? *FEBS Lett.* 594(24):4189–4200

Türei D, Korcsmáros T, Saez-Rodriguez J. 2016. OmniPath: guidelines and gateway for literature-curated signaling pathway resources. *Nat. Methods*. 13(12):966–67

Türei D, Valdeolivas A, Gul L, Palacio- Escat N, Klein M, et al. 2021. Integrated intra- and intercellular signaling knowledge for multicellular omics analysis. *Mol. Syst. Biol.* 17(3):

Zhao M, Wang L, Wang M, Zhou S, Lu Y, et al. 2022. Targeting fibrosis, mechanisms and cilinical trials. *Signal Transduct. Target. Ther.* 7(1):206

Zhou S, Yin X, Mayr M, Noor M, Hylands PJ, Xu Q. 2020. Proteomic landscape of TGF-β1-induced fibrogenesis in renal fibroblasts. *Sci. Rep.* 10(1):19054

Zi Z, Chapnick DA, Liu X. 2012. Dynamics of TGF-β/Smad signaling. *FEBS Lett.* 586(14):1921–28

4th Apr 2025

Manuscript Number: MSB-2025-12898R
Title: Dynamic multi-omics and mechanistic modeling approach uncovers novel mechanisms of kidney fibrosis progression

Dear Dr. Pepperkok,

Thank you for the submission of your revised manuscript to Molecular Systems Biology. Below you will find the re-review reports from two of the three original reviewers. In addition to the review submitted by Reviewer 1, they also commented on Reviewer 2's concerns and your responses (as Reviewer 2 was not available for re-review). Reviewer 1 said that the authors fairly responded to what was pointed out and that further experimental verification as suggested by Reviewer 2 is not necessary. Therefore I am pleased to inform you that we will be able to accept your manuscript pending the following final amendments and appropriate response to the remaining point from Reviewer 3.

1) Please update the "Author Checklist" in the general information boxes at the top (Corresponding author name, etc).
2) Please include a Data availability section describing how the data, code etc. have been made available. This section needs to be formatted according to the example below (please be sure to provide specific URLs for the PXD056096 and E-MTAB-14521 datasets):
"The datasets and computer code produced in this study are available in the following databases:
- Chip-Seq data: Gene Expression Omnibus GSE46748 (https://www.ncbi.nlm.nih.gov/geo/query/acc.cgi?acc=GSE46748)
- Modeling computer scripts: GitHub (https://github.com/SysBioChalmers/GECKO/releases/tag/v1.0)
- [data type]: [full name of the resource] [accession number/identifier] ([doi or URL or identifiers.org/DATABASE:ACCESSION])"
3) Data Availability: Please rename the 'Data and code availability' section to 'Data Availability'.
4) Data availability: Please now release the proteomics dataset in PXD056096 so that it is publicly available. Please be aware that all deposited datasets should be freely accessible prior to publication.
5) Please rename "Conflicts of Interests" to "Disclosure and competing interests statement". We updated our journal's competing interests policy in January 2022 and request authors to consider both actual and perceived competing interests. Please review the policy https://www.embopress.org/competing-interests and update your competing interests if necessary.
6) Author contributions: Please remove it from the manuscript and specify author contributions in our submission system. CRediT has replaced the traditional author contributions section because it offers a systematic machine-readable author contributions format that allows for more effective research assessment. You are encouraged to use the free text boxes beneath each contributing author's name to add specific details on the author's contribution. More information is available in our guide to authors:
https://www.embopress.org/page/journal/17574684/authorguide#authorshipguidelines
7) References: Please correct the reference citation in the reference list to be alphabetical (not numerical). Where there are more than 10 authors on a paper, only the first 10 should be listed, followed by "et al.". Please check "Author Guidelines" for more information.
https://www.embopress.org/page/journal/17574684/authorguide#referencesformat
8) Our journal encourages inclusion of *data citations in the reference list* to directly cite datasets that were re-used and obtained from public databases. Data citations in the article text are distinct from normal bibliographical citations and should directly link to the database records from which the data can be accessed. In the main text, data citations are formatted as follows: "Data ref: Smith et al, 2001" or "Data ref: NCBI Sequence Read Archive PRJNA342805, 2017". In the Reference list, data citations must be labeled with "[DATASET]". A data reference must provide the database name, accession number/identifiers and a resolvable link to the landing page from which the data can be accessed at the end of the reference. Further instructions are available at .
9) In the Methods, please take care of the following:
- The Materials and Methods section should be renamed to "Methods".
- It is currently unclear whether you have used an established cell line or cells from a human research participant, as it was indicated in the Author Checklist that a cell line has been used, but this information is not included in the Reagents and Tools table, and additionally the Methods state that the cells were isolated from a patient. The use of human samples requires information on the authority granting ethics approval (e.g. IRB) and informed consent. If the need for approval is waived, please cite the reason (e.g. non-human subject research because the samples used were de-identified/coded with no identifying information) and legislation in the relevant methods section. If the study does involve a human research participant, please also state that the experiments conformed to the principles set out in the WMA Declaration of Helsinki and the Department of Health and Human Services Belmont Report. The Author Checklist should also be updated with this information. If these cells are rather an established cell line, please be sure to include the appropriate information in the Reagents and Tools table and add a sentence in the Methods as to whether or not the cell lines were recently authenticated.
- Please ensure that a statement on whether or not blinding was done is included in the Methods even if no blinding was done. Please also be sure to update the Author Checklist with this information and where it can be found in the manuscript.
10) Please remove the Reagents and Tools Table from the Methods section of the manuscript and upload it as a separate file choosing the file type "Reagent Table".
11) Please place individual sections of the manuscript in the following order: Title page - Abstract & Keywords - Introduction -

Results - Discussion - Methods - Data Availability - Acknowledgements - Disclosure and Competing Interests Statement - References - Figure Legends - Expanded View Figure Legends.
12) For the figures and figure legends, please take care of the following:
- Please ensure that all figures are called out sequentially. Please also double check the callout for Figure E54C, as there is no such figure uploaded.
- Please note that the exact p values are not provided in the legends of figures 1C, 4B, D, H; EV7 A, C, F.
- Please indicate the statistical test used for data analysis in the legends of figures 1C, EV3 C, EV5 D, EV5 E, EV6 C.
- Please note that information related to n is missing in the legends of figures EV5 E, EV6 E.
13) Tables: Please rename Tables S1-S5 and S8-S10 to Dataset EV1-EV8 (source file names, titles, legends and manuscript callouts all need to be updated). These should be uploaded individually as Dataset files with legends in a separate tab/sheet in each Excel file; Tables S6 and S7 should be renamed to Table EV1-EV2 with the legends uploaded above the table in each Excel file. In addition, the "Supplementary Materials" section in the main manuscript should be removed, as the legends should be included in the individual Excel files.
14) Funding: Please ensure that all funding sources are listed in both the "Acknowledgements" section and are entered into the manuscript submission system.
15) Synopsis:
- Synopsis image: The dimensions of the synopsis image should be 550 pixels wide x (300-600) pixels high. Although we can resize the image for you, currently with a width of 550 pixels, the figure is not sufficiently high (197 pixels). Please reorganize the figure to fit within our requested dimensions.
- Synopsis text: Please provide a short standfirst (maximum of 300 characters, including space), limit the bullet points to max. 5 and upload it as a separate .doc file. Please write the bullet points to summarise the key NEW findings. They should be designed to be complementary to the abstract - i.e. not repeat the same text. We encourage inclusion of key acronyms and quantitative information (maximum of 30 words / bullet point). Please use the passive voice.
- Please check your synopsis text and image before submission with your revised manuscript. Please be aware that in the proof stage minor corrections only are allowed (e.g., typos).
16) Source Data: Thank you for providing the Source Data for Figure EV2. We have checked the figure and the blot looks fine. We would like to publish the source data for this figure along with the accepted manuscript in case a reader would like to take a look at the file.
17) As part of the EMBO Publications transparent editorial process initiative (see our policy here: https://www.embopress.org/transparent-process#Review_Process), Molecular Systems Biology will publish online a Peer Review File (PRF) to accompany accepted manuscripts. This file will be published in conjunction with your paper and will include the anonymous referee reports, your point-by-point response and all pertinent correspondence relating to the manuscript. Let us know whether you agree with the publication of the PRF and as here, if you want to remove or not any figures from it prior to publication. Please note that the Authors checklist will be published at the end of the PRF.
18) After your paper is published, we will promote it on social media. If you have any handles or hashtags for Bluesky you would like included, please let us know.
19) Please provide a point-by-point letter INCLUDING my comments as well as the reviewer's reports and your detailed responses (as Word file).

I look forward to reading a new revised version of your manuscript as soon as possible.

Yours sincerely,

Poonam Bheda, PhD
Scientific Editor
Molecular Systems Biology

Reviewer #1:

In this revised manuscript the authors have adequately addressed the concerns that I raised , and I'm supportive of accepting this manuscript.

Reviewer #3:

In general, the authors appropriately responded to my concerns.
The only major concern that remains is what is argued in "Answer 1.8".
I believe that It is not appropriate to use any fitting because nothing is known about the (statistical or kinetic) model behind these time courses.

Connect the time points as line graphs.

***
Rev_Com_number: RC-2024-02741
New_manu_number: MSB-2025-12898R
Corr_author: Pepperkok
Title: Dynamic multi-omics and mechanistic modeling approach uncovers novel mechanisms of kidney fibrosis progression

Point by point response letter

1) Please update the "Author Checklist" in the general information boxes at the top (Corresponding author name, etc).

   Response 1) We updated the document and added the missing information.

2) Please include a Data availability section describing how the data, code etc. have been made available. This section needs to be formatted according to the example below (please be sure to provide specific URLs for the PXD056096 and E-MTAB-14521 datasets):
   "The datasets and computer code produced in this study are available in the following databases:
   - Chip-Seq data: Gene Expression Omnibus GSE46748 (https://www.ncbi.nlm.nih.gov/geo/query/acc.cgi?acc=GSE46748)
   - Modeling computer scripts: GitHub (https://github.com/SysBioChalmers/GECKO/releases/tag/v1.0)
   - [data type]: [full name of the resource] [accession number/identifier] ([doi or URL or identifiers.org/DATABASE:ACCESSION])"

   Response 2) Please find the data availability section with the according format in the updated manuscript.

3) Data Availability: Please rename the 'Data and code availability' section to 'Data Availability'.

   Response 3) We have renamed the section accordingly.

4) Data availability: Please now release the proteomics dataset in PXD056096 so that it is publicly available. Please be aware that all deposited datasets should be freely accessible prior to publication.

   Response 4) The proteomics dataset was made publicly available.

5) Please rename "Conflicts of Interests" to "Disclosure and competing interests statement". We updated our journal's competing interests policy in January 2022 and request authors to consider both actual and perceived competing interests. Please review the policy https://www.embopress.org/competing-interests and update your competing interests if necessary.

   Response 5) We renamed the section and confirmed our statements with the updated regulations.

6) Author contributions: Please remove it from the manuscript and specify author contributions in our submission system. CRediT has replaced the traditional author contributions section because it offers a systematic machine-readable author contributions format that allows for more effective research assessment. You are encouraged to use the free text boxes beneath each contributing author's name to add specific details on the author's contribution. More information is available in our guide to authors: https://www.embopress.org/page/journal/17574684/authorguide#authorship

guidelines

Response 6) We removed the section from the manuscript.

7) References: Please correct the reference citation in the reference list to be alphabetical (not numerical). Where there are more than 10 authors on a paper, only the first 10 should be listed, followed by "et al.". Please check "Author Guidelines" for more information. https://www.embopress.org/page/journal/17574684/authorguide#referencesformat

Response 7) The authors thank the editor for the information and updated the references in the text as well as the reference list in the manuscript accordingly.

8) Our journal encourages inclusion of *data citations in the reference list* to directly cite datasets that were re-used and obtained from public databases. Data citations in the article text are distinct from normal bibliographical citations and should directly link to the database records from which the data can be accessed. In the main text, data citations are formatted as follows: "Data ref: Smith et al, 2001" or "Data ref: NCBI Sequence Read Archive PRJNA342805, 2017". In the Reference list, data citations must be labeled with "[DATASET]". A data reference must provide the database name, accession number/identifiers and a resolvable link to the landing page from which the data can be accessed at the end of the reference. Further instructions are available at https://www.embopress.org/page/journal/17574684/authorguide#referencesformat.

Response 8) Our manuscript does not include datasets from a database. All data was extracted from supplementary material of the cited publications.

9) In the Methods, please take care of the following:
- The Materials and Methods section should be renamed to "Methods".

- It is currently unclear whether you have used an established cell line or cells from a human research participant, as it was indicated in the Author Checklist that a cell line has been used, but this information is not included in the Reagents and Tools table, and additionally the Methods state that the cells were isolated from a patient. The use of human samples requires information on the authority granting ethics approval (e.g. IRB) and informed consent. If the need for approval is waived, please cite the reason (e.g. non-human subject research because the samples used were de-identified/coded with no identifying information) and legislation in the relevant methods section. If the study does involve a human research participant, please also state that the experiments conformed to the principles set out in the WMA Declaration of Helsinki and the Department of Health and Human Services Belmont Report. The Author Checklist should also be updated with this information. If these cells are rather an established cell line, please be sure to include the appropriate information in the Reagents and Tools table and add a sentence in the Methods as to

whether or not the cell lines were recently authenticated.

- Please ensure that a statement on whether or not blinding was done is included in the Methods even if no blinding was done. Please also be sure to update the Author Checklist with this information and where it can be found in the manuscript.

Response 9)
- We have renamed the section accordingly.
- We appreciate the editors feedback in this regards and have added a paragraph to the methods section:

Ethics
The local ethics committee of the University Hospital RWTH Aachen approved the generation of cells (EK-016/17). Kidney tissue was collected from the Urology Department of the Hospital Eschweiler from patients undergoing (partial) nephrectomy (Kuppe et al, 2021; Bouwens et al, 2025). All patients provided informed consent, and the study was performed in accordance with the Declaration of Helsinki.

More information about the cell line can be found in the publications of Kuppe et al, 2021 and Bouwens et al, 2025, which we also linked in the Methods section. Additionally, we updated the author checklist accordingly.

- No blinding was performed. We added this information in the method section and the author checklist.

10) Please remove the Reagents and Tools Table from the Methods section of the manuscript and upload it as a separate file choosing the file type "Reagent Table".

Response 10) The authors thank the editor for the information and removed the reagent and tools table from the Methods section. In addition, a new file with the name “Reagent Table.docx” has been uploaded.

11) Please place individual sections of the manuscript in the following order: Title page - Abstract & Keywords - Introduction - Results - Discussion - Methods - Data Availability - Acknowledgements - Disclosure and Competing Interests Statement - References - Figure Legends - Expanded View Figure Legends.

Response 11) Please find the new order in the updated manuscript version.

12) For the figures and figure legends, please take care of the following:
- Please ensure that all figures are called out sequentially. Please also double check the callout for Figure E54C, as there is no such figure uploaded.

Response 12) We corrected the typo in the figure label.

- Please note that the exact p values are not provided in the legends of figures 1C, 4B, D, H; EV7 A, C, F.

Response 12) We provided p-values for these figures in the figure or figure legend. Please notice a we made already in our last response to this comment (Document MSB requirements, which we included in our revision upload). For Figure 1C we used a test function (emmeans) which does not return exact p-values, wherefore we have to report the significance level only. We extended our description of the test to make this hopefully clearer.

- Please indicate the statistical test used for data analysis in the legends of figures 1C, EV3 C, EV5 D, EV5 E, EV6 C.

Response 12) We added this information in the corresponding figure legend.

- Please note that information related to n is missing in the legends of figures EV5 E, EV6 E.

Response 12) We added this information in the corresponding figure legend.

13) Tables: Please rename Tables S1-S5 and S8-S10 to Dataset EV1-EV8 (source file names, titles, legends and manuscript callouts all need to be updated). These should be uploaded individually as Dataset files with legends in a separate tab/sheet in each Excel file; Tables S6 and S7 should be renamed to Table EV1-EV2 with the legends uploaded above the table in each Excel file. In addition, the "Supplementary Materials" section in the main manuscript should be removed, as the legends should be included in the individual Excel files.

    Response 13) We updated all tables and datasets as well as their links accordingly.

14) Funding: Please ensure that all funding sources are listed in both the "Acknowledgements" section and are entered into the manuscript submission system.

    Response 14) We thank the editor for the information and updated our Acknowledgements and the manuscript submission system accordingly.

15) Synopsis:
    - Synopsis image: The dimensions of the synopsis image should be 550 pixels wide x (300-600) pixels high. Although we can resize the image for you, currently with a width of 550 pixels, the figure is not sufficiently high (197 pixels). Please reorganize the figure to fit within our requested dimensions.
    - Synopsis text: Please provide a short standfirst (maximum of 300 characters, including space), limit the bullet points to max. 5 and upload it as a separate .doc file. Please write the bullet points to summarise the key NEW

findings. They should be designed to be complementary to the abstract - i.e. not repeat the same text. We encourage inclusion of key acronyms and quantitative information (maximum of 30 words / bullet point). Please use the passive voice.
- Please check your synopsis text and image before submission with your revised manuscript. Please be aware that in the proof stage minor corrections only are allowed (e.g., typos).

Response 15) We thank the editor for this information and have adjusted the synopsis image accordingly (please see “Synopsis.jpg”) and provided the synopsis text again in a separate file (“Synopsis.docx”).

16) Source Data: Thank you for providing the Source Data for Figure EV2. We have checked the figure and the blot looks fine. We would like to publish the source data for this figure along with the accepted manuscript in case a reader would like to take a look at the file.

Response 16) We thank the editor for this information and agree to publish the source data along with the manuscript.

17) As part of the EMBO Publications transparent editorial process initiative (see our policy here: https://www.embopress.org/transparent-process#Review_Process), Molecular Systems Biology will publish online a Peer Review File (PRF) to accompany accepted manuscripts. This file will be published in conjunction with your paper and will include the anonymous referee reports, your point-by-point response and all pertinent correspondence relating to the manuscript. Let us know whether you agree with the publication of the PRF and as here, if you want to remove or not any figures from it prior to publication. Please note that the Authors checklist will be published at the end of the PRF.

Response 17) We appreciate this information and agree with the publication of the PRF.

18) After your paper is published, we will promote it on social media. If you have any handles or hashtags for Bluesky you would like included, please let us know.

Response 18) Please include savitski-lab.bsky.social, saezlab.bsky.social, miraburtscher.bsky.social

19) Please provide a point-by-point letter INCLUDING my comments as well as the reviewer's reports and your detailed responses (as Word file).

Response 19) We hope that we could clarify all open points in this point-by-point response letter.

Reviewer #1:

In this revised manuscript the authors have adequately addressed the concerns that I raised , and I'm supportive of accepting this manuscript.

Response: We appreciate the reviewers' support and input during the review process.

Reviewer #3:

In general, the authors appropriately responded to my concerns.

The only major concern that remains is what is argued in "Answer 1.8".

I believe that It is not appropriate to use any fitting because nothing is known about the (statistical or kinetic) model behind these time courses.

Connect the time points as line graphs.

We have adapted following figures according to the reviewers request: Figure 3, Figure 4 and Figure EV7.

25th Apr 2025
Manuscript number: MSB-2025-12898RR
Title: Dynamic multi-omics and mechanistic modeling approach uncovers novel mechanisms of kidney fibrosis progression

Dear Dr. Pepperkok,

Thank you again for sending us your revised manuscript. We are now satisfied with the modifications made and I am pleased to inform you that your paper has been accepted for publication.

Your manuscript will be processed for publication by EMBO Press. It will be copy edited and you will receive page proofs prior to publication. Please note that you will be contacted by Springer Nature Author Services to complete licensing and payment information.

You may qualify for financial assistance for your publication charges - either via a Springer Nature fully open access agreement or an EMBO initiative. Check your eligibility: https://www.embopress.org/page/journal/17444292/authorguide#chargesguide

Should you be planning a Press Release on your article, please get in contact with embo_production@springernature.com as early as possible in order to coordinate publication and release dates.

If you have any questions, please do not hesitate to contact the Editorial Office. Thank you for your contribution to Molecular Systems Biology.

Yours sincerely,

Sincerely,

Poonam Bheda, PhD
Scientific Editor
Molecular Systems Biology

>>> Please note that it is Molecular Systems Biology policy for the transcript of the editorial process (containing referee reports and your response letter) to be published as an online supplement to each paper. If you do NOT want this, you will need to inform the Editorial Office via email immediately. More information is available here: https://www.embopress.org/transparent-process#Review_Process

EMBO Press Author Checklist

Corresponding Author Name: Dr. Rainer Pepperkok	
Journal Submitted to: Molecular Systems Biology	
Manuscript Number: MSB-2025-12898R	

USEFUL LINKS FOR COMPLETING THIS FORM
The EMBO Journal - Author Guidelines
EMBO Reports - Author Guidelines
Molecular Systems Biology - Author Guidelines
EMBO Molecular Medicine - Author Guidelines

Reporting Checklist for Life Science Articles (updated January
This checklist is adapted from Materials Design Analysis Reporting (MDAR) Checklist for Authors. MDAR establishes a minimum set of requirements in transparent reporting in the life sciences (see Statement of Task: 10.31222/osf.io/9sm4x). Please follow the journal's guidelines in preparing your manuscript.
Please note that a copy of this checklist will be published alongside your article.

Abridged guidelines for figures
1. Data
The data shown in figures should satisfy the following conditions:
- the data were obtained and processed according to the field's best practice and are presented to reflect the results of the experiments in an accurate and unbiased manner.
- ideally, figure panels should include only measurements that are directly comparable to each other and obtained with the same assay.
- plots include clearly labeled error bars for independent experiments and sample sizes. Unless justified, error bars should not be shown for technical
- if n<5, the individual data points from each experiment should be plotted. Any statistical test employed should be justified.
- Source Data should be included to report the data underlying figures according to the guidelines set out in the authorship guidelines on Data

2. Captions
Each figure caption should contain the following information, for each panel where they are relevant:
- a specification of the experimental system investigated (eg cell line, species name).
- the assay(s) and method(s) used to carry out the reported observations and measurements.
- an explicit mention of the biological and chemical entity(ies) that are being measured.
- an explicit mention of the biological and chemical entity(ies) that are altered/varied/perturbed in a controlled manner.
- the exact sample size (n) for each experimental group/condition, given as a number, not a range;
- a description of the sample collection allowing the reader to understand whether the samples represent technical or biological replicates (including how many animals, litters, cultures, etc.).
- a statement of how many times the experiment shown was independently replicated in the laboratory.
- definitions of statistical methods and measures:

- common tests, such as t-test (please specify whether paired vs. unpaired), simple χ2 tests, Wilcoxon and Mann-Whitney tests, can be unambiguously identified by name only, but more complex techniques should be described in the methods section;

- are tests one-sided or two-sided?
- are there adjustments for multiple comparisons?
- exact statistical test results, e.g., P values = x but not P values < x;
- definition of 'center values' as median or average;
- definition of error bars as s.d. or s.e.m.

Please complete ALL of the questions below.
Select "Not Applicable" only when the requested information is not relevant for your study.

Materials

Newly Created Materials	Information included in the manuscript?	In which section is the information available? (Reagents and Tools Table, Materials and Methods, Figures, Data Availability Section)
New materials and reagents need to be available; do any restrictions apply?	Yes	Reagents and Tools Table, Methods

Antibodies	Information included in the manuscript?	In which section is the information available? (Reagents and Tools Table, Materials and Methods, Figures, Data Availability Section)
For **antibodies** provide the following information:<br>- Commercial antibodies: RRID (if possible) or supplier name, catalogue number and or/clone number<br>- Non-commercial: RRID or citation	Yes	Reagents and Tools Table, Methods

DNA and RNA sequences	Information included in the manuscript?	In which section is the information available? (Reagents and Tools Table, Materials and Methods, Figures, Data Availability Section)
**Short novel DNA or RNA including primers, probes**: provide the sequences.	Yes	EV Tables

Cell materials	Information included in the manuscript?	In which section is the information available? (Reagents and Tools Table, Materials and Methods, Figures, Data Availability Section)
**Cell lines:** Provide species information, strain. Provide accession number in repository **OR** supplier name, catalog number, clone number, and/**OR** RRID.	Not Applicable	N/A
**Primary cultures:** Provide species, strain, sex of origin, genetic modification status.	Yes	Methods
Report if the cell lines were recently **authenticated** (e.g., by STR profiling) and tested for mycoplasma contamination.	Yes	Methods

Experimental animals	Information included in the manuscript?	In which section is the information available? (Reagents and Tools Table, Materials and Methods, Figures, Data Availability Section)
**Laboratory animals or Model organisms:** Provide species, strain, sex, age, genetic modification status. Provide accession number in repository **OR** supplier name, catalog number, clone number, **OR** RRID.	Not Applicable	N/A
**Animal observed in or captured from the field:** Provide species, sex, and age where possible.	Not Applicable	N/A
Please detail **housing and husbandry conditions**.	Not Applicable	N/A

Plants and microbes	Information included in the manuscript?	In which section is the information available? (Reagents and Tools Table, Materials and Methods, Figures, Data Availability Section)
**Plants:** provide species and strain, ecotype and cultivar where relevant, unique accession number if available, and source (including location for collected wild specimens).	Not Applicable	N/A
**Microbes:** provide species and strain, unique accession number if available, and source.	Not Applicable	N/A

Human research participants	Information included in the manuscript?	In which section is the information available? (Reagents and Tools Table, Materials and Methods, Figures, Data Availability Section)
If collected and within the bounds of privacy constraints report on age, sex and gender or ethnicity for all study participants.	Not Applicable	N/A

Core facilities	Information included in the manuscript?	In which section is the information available? (Reagents and Tools Table, Materials and Methods, Figures, Data Availability Section)
If your work benefited from core facilities, was their service mentioned in the acknowledgments section?	Yes	Author list, Acknowledgements

Design

Study protocol	Information included in the manuscript?	In which section is the information available? (Reagents and Tools Table, Materials and Methods, Figures, Data Availability Section)
If study protocol has been **pre-registered, provide DOI in the manuscript**. For clinical trials, provide the trial registration number **OR** cite DOI.	Not Applicable	N/A
Report the **clinical trial registration number** (at ClinicalTrials.gov or equivalent), where applicable.	Not Applicable	N/A

Laboratory protocol	Information included in the manuscript?	In which section is the information available? (Reagents and Tools Table, Materials and Methods, Figures, Data Availability Section)
Provide DOI OR other citation details if **external detailed step-by-step protocols** are available.	Not Applicable	N/A

Experimental study design and statistics	Information included in the manuscript?	In which section is the information available? (Reagents and Tools Table, Materials and Methods, Figures, Data Availability Section)
Include a statement about **sample size** estimate even if no statistical methods were used.	Yes	manuscript text, figure legends
Were any steps taken to minimize the effects of subjective bias when allocating animals/samples to treatment (e.g. **randomization procedure**)? If yes, have they been described?	Not Applicable	N/A
Include a statement about **blinding** even if no blinding was done.	Yes	method section
Describe **inclusion/exclusion criteria** if samples or animals were excluded from the analysis. Were the criteria pre-established?<br><br>If sample or data points were omitted from analysis, report if this was due to attrition or intentional exclusion and provide justification.	Yes	manuscript text, figure legends
For every figure, are **statistical tests** justified as appropriate? Do the data meet the assumptions of the tests (e.g., normal distribution)? Describe any methods used to assess it. Is there an estimate of variation within each group of data? Is the variance similar between the groups that are being statistically compared?	Yes	manuscript text, figure legends

Sample definition and in-laboratory replication	Information included in the manuscript?	In which section is the information available? (Reagents and Tools Table, Materials and Methods, Figures, Data Availability Section)
In the figure legends: state number of times the experiment was **replicated** in laboratory.	Yes	manuscript text, figure legends
In the figure legends: define whether data describe **technical or biological replicates**.	Yes	manuscript text, figure legends

Ethics

Ethics	Information included in the manuscript?	In which section is the information available? (Reagents and Tools Table, Materials and Methods, Figures, Data Availability Section)
Studies involving **human participants**: State details of **authority granting ethics approval** (IRB or equivalent committee(s), provide reference number for approval.	Yes	Methods
Studies involving **human participants**: Include a statement confirming that **informed consent** was obtained from all subjects and that the experiments conformed to the principles set out in the WMA Declaration of Helsinki and the Department of Health and Human Services Belmont Report.	Yes	Methods
Studies involving **human participants:** For publication of **patient photos**, include a statement confirming that consent to publish was obtained.	Not Applicable	N/A
Studies involving experimental **animals**: State details of **authority granting ethics approva**l (IRB or equivalent committee(s), provide reference number for approval. Include a statement of compliance with ethical regulations.	Not Applicable	N/A
Studies involving **specimen and field samples:** State if relevant **permits** obtained, provide details of authority approving study; if none were required, explain why.	Not Applicable	N/A

Dual Use Research of Concern (DURC)	Information included in the manuscript?	In which section is the information available? (Reagents and Tools Table, Materials and Methods, Figures, Data Availability Section)
Could your study fall under dual use research restrictions? Please check biosecurity documents and list of **select agents and toxins** (CDC): https://www.selectagents.gov/sat/list.htm	Not Applicable	N/A
If you used a select agent, is the security level of the lab appropriate and reported in the manuscript?	Not Applicable	N/A
If a study is subject to dual use research of concern regulations, is the name of the **authority granting approval and reference number** for the regulatory approval provided in the manuscript?	Not Applicable	N/A

Reporting

The MDAR framework recommends adoption of discipline-specific guidelines, established and endorsed through community initiatives. Journals have their own policy about requiring specific guidelines and recommendations to complement MDAR.

Adherence to community standards	Information included in the manuscript?	In which section is the information available? (Reagents and Tools Table, Materials and Methods, Figures, Data Availability Section)
State if relevant guidelines or checklists (e.g., **ICMJE, MIBBI, ARRIVE, PRISMA**) have been followed or provided.	Not Applicable	N/A
For **tumor marker prognostic studies,** we recommend that you follow the **REMARK** reporting guidelines (see link list at top right). See author guidelines, under 'Reporting Guidelines'. Please confirm you have followed these guidelines.	Not Applicable	N/A
For **phase II and III randomized controlled trials**, please refer to the **CONSORT** flow diagram (see link list at top right) and submit the CONSORT checklist (see link list at top right) with your submission. See author guidelines, under 'Reporting Guidelines'. Please confirm you have submitted this list.	Not Applicable	N/A

Data Availability

Data availability	Information included in the manuscript?	In which section is the information available? (Reagents and Tools Table, Materials and Methods, Figures, Data Availability Section)
Have **primary datasets** been deposited according to the journal's guidelines (see 'Data Deposition' section) and the respective accession numbers provided in the Data Availability Section?	Yes	Data Availability Section
Were **human clinical and genomic datasets** deposited in a public access-controlled repository in accordance to ethical obligations to the patients and to the applicable consent agreement?	Yes	Data Availability Section
Are **computational models** that are central and integral to a study available without restrictions in a machine-readable form? Were the relevant accession numbers or links provided?	Yes	Data Availability Section
If publicly available data were reused, provide the respective **data citations in the reference list**.	Yes	Data Availability Section
