# Supplementary figures and images for: Dynamic multi-omics and mechanistic modeling approach uncovers novel mechanisms of kidney fibrosis progression
Source: Mol Syst Biol. 2025 Jun 5;21(8):1030–65. doi: 10.1038/s44320-025-00116-2 (PMC12322177; doi:10.1038/s44320-025-00116-2)

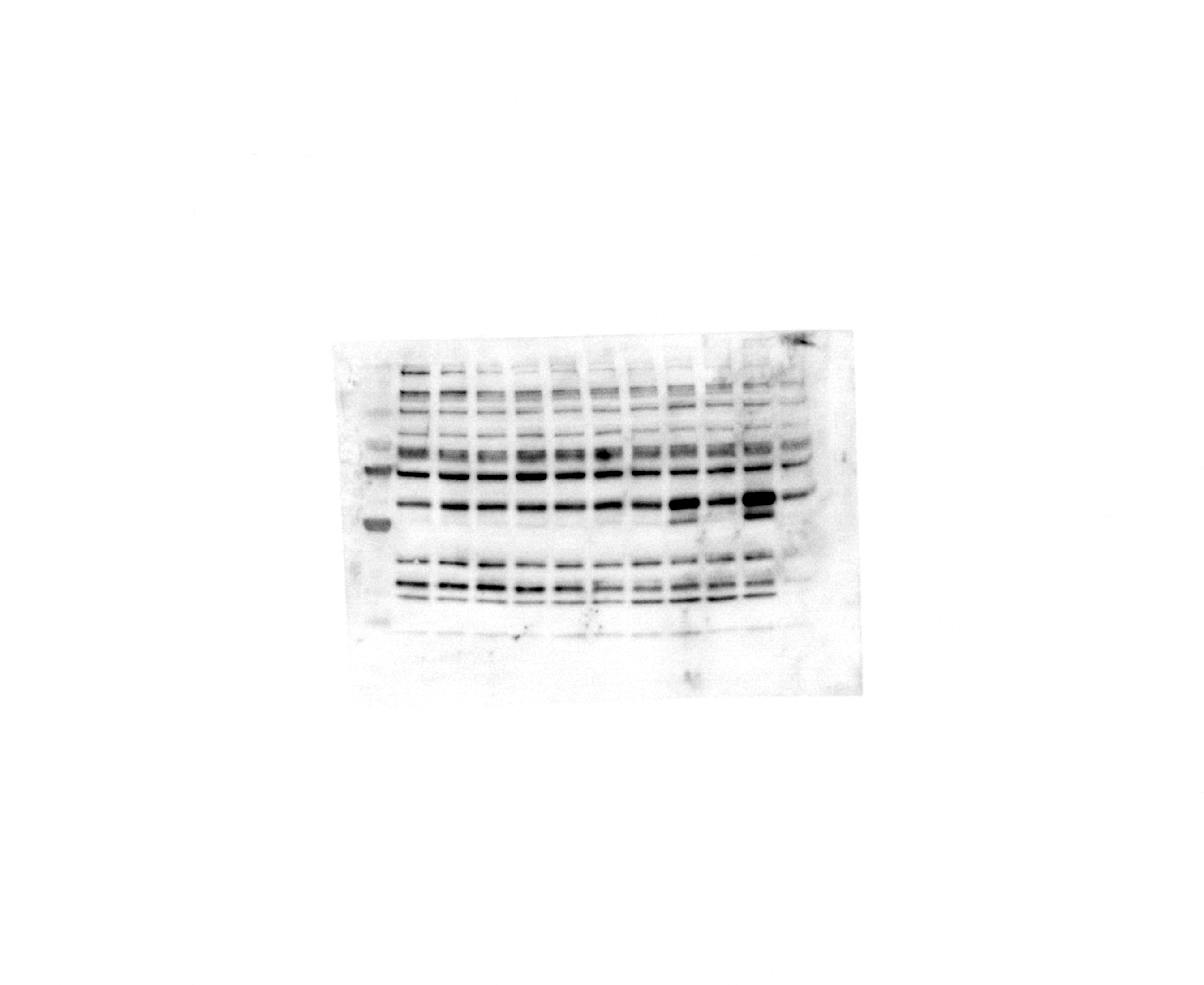

Supplement: Supplementary file 12 — Source Data Fig EV2 [file 44320_2025_116_MOESM12_ESM.zip › Fig EV2/Fig EV2A/pSMAD2_membrane1_30sec-10min_21.09.23_12.36.22blot_PUB_600 (1).tif]

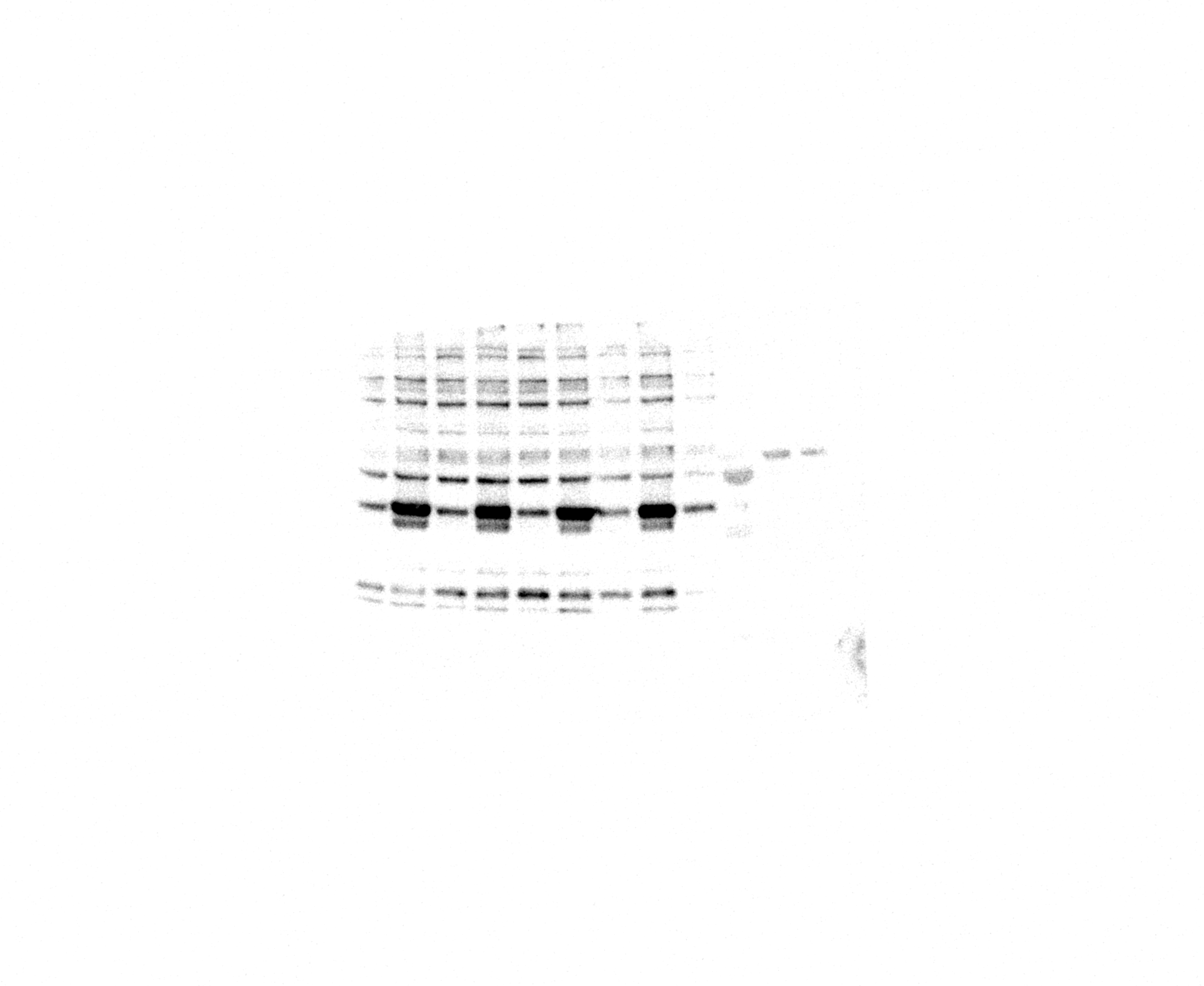

Supplement: Supplementary file 12 — Source Data Fig EV2 [file 44320_2025_116_MOESM12_ESM.zip › Fig EV2/Fig EV2A/pSMAD2_membrane3_30min-6h_21.09.23_12.44.24_PUB_600copy.tif]

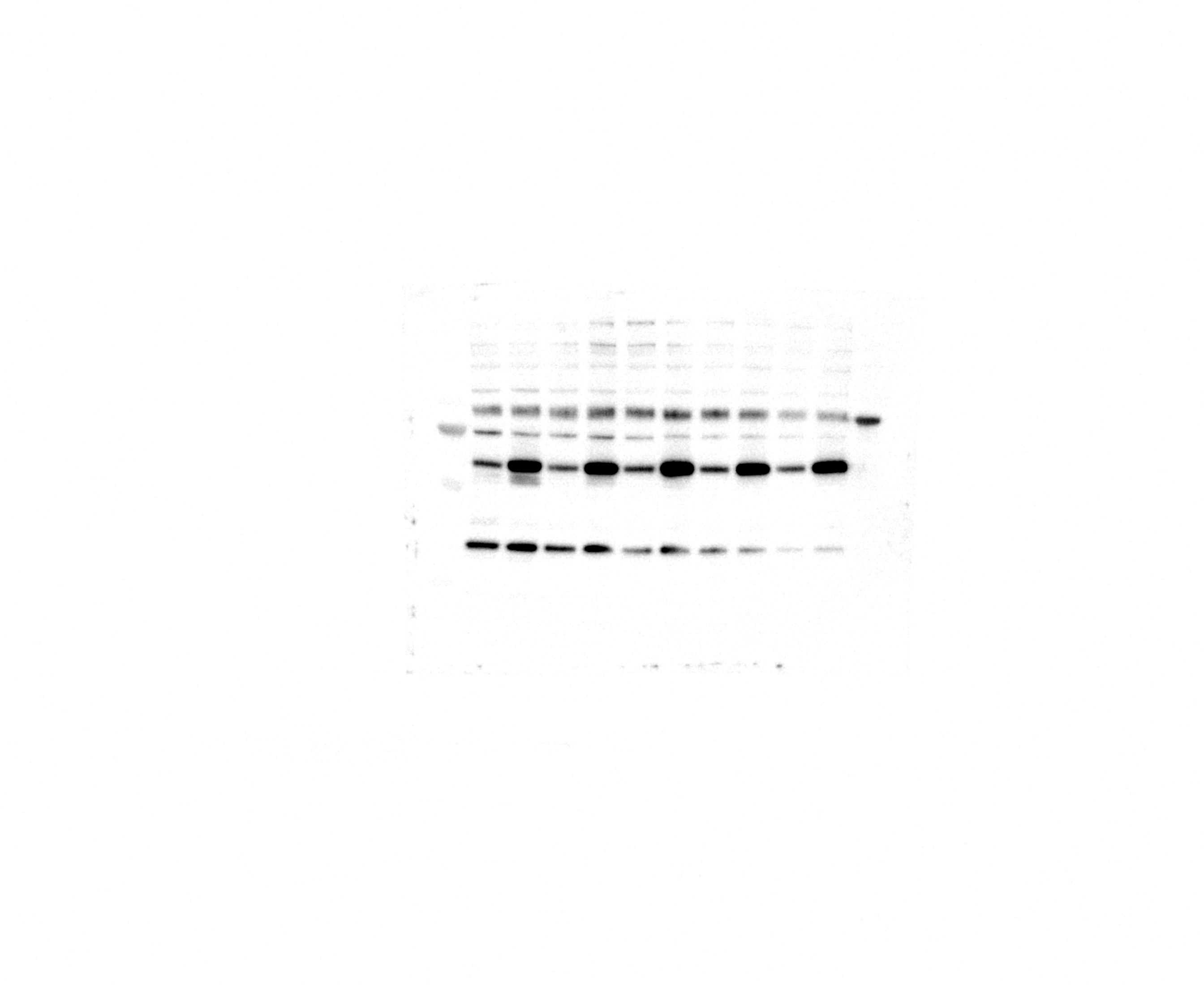

Supplement: Supplementary file 12 — Source Data Fig EV2 [file 44320_2025_116_MOESM12_ESM.zip › Fig EV2/Fig EV2A/pSMAD2_membrane6_12-96h_21.09.28_11.43.48_PUB_600.tif]

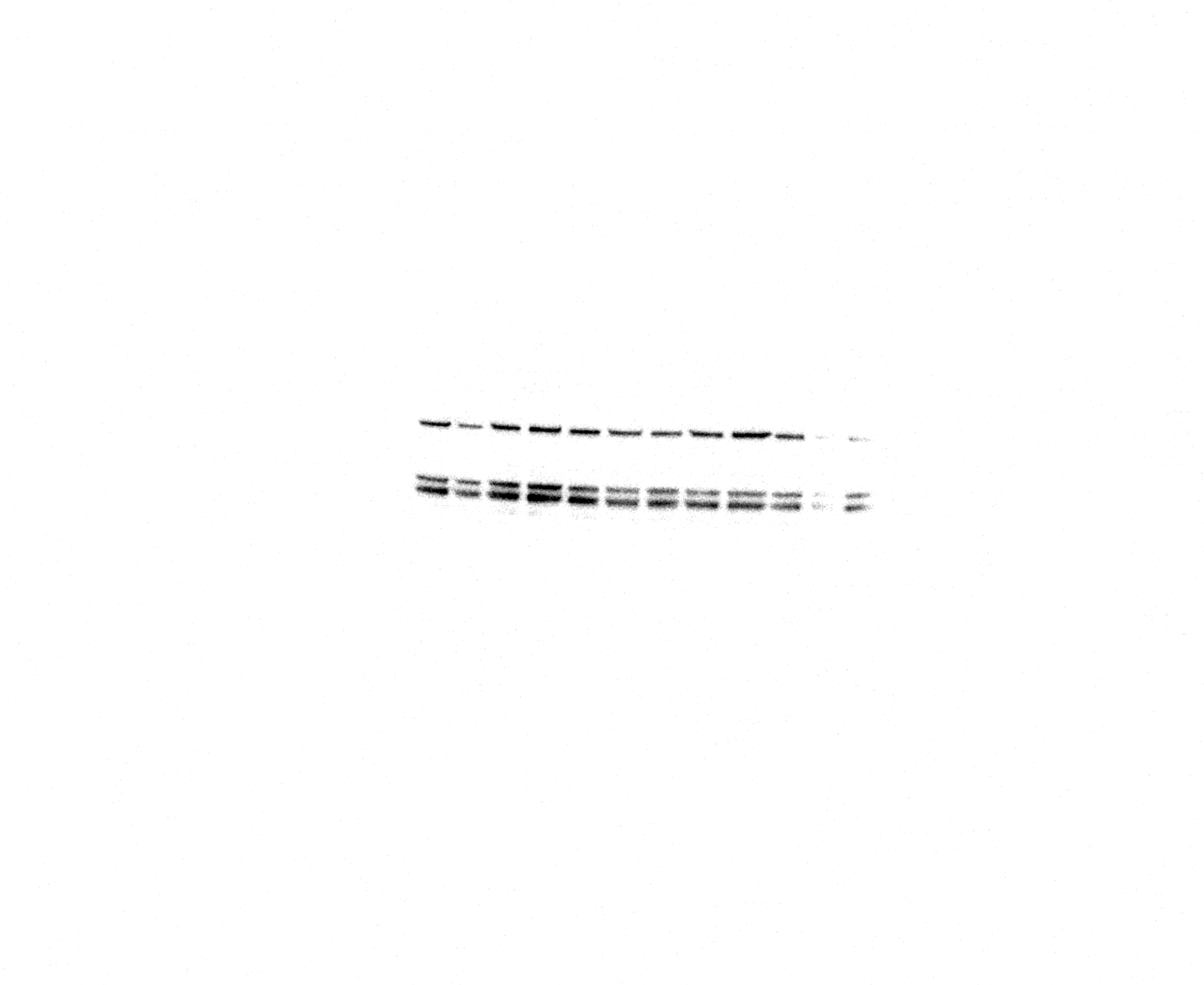

Supplement: Supplementary file 12 — Source Data Fig EV2 [file 44320_2025_116_MOESM12_ESM.zip › Fig EV2/Fig EV2A/SMAD2_membrane2_30sec-10min_21.09.23_12.16.45blo_PUB_600.tif]

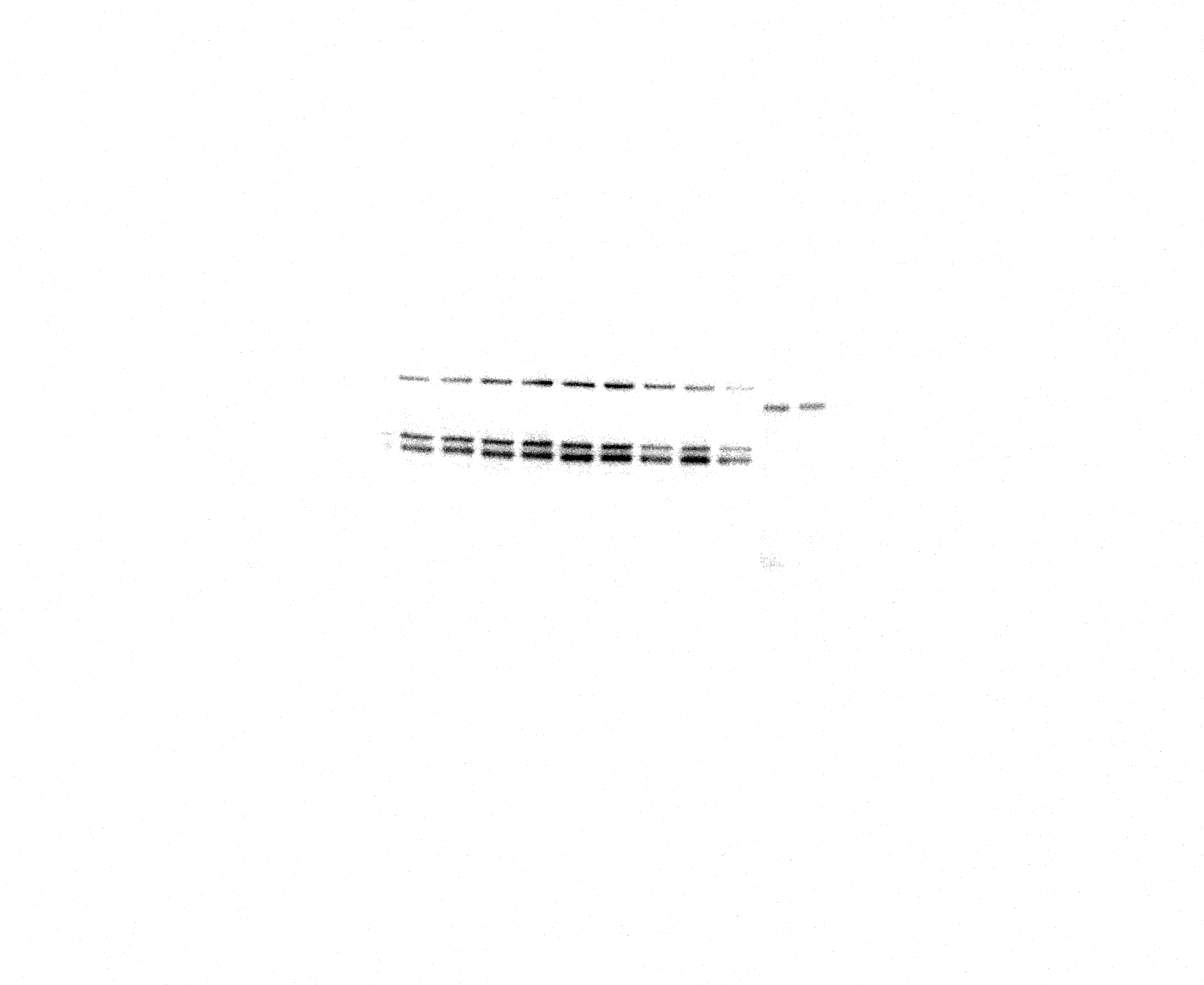

Supplement: Supplementary file 12 — Source Data Fig EV2 [file 44320_2025_116_MOESM12_ESM.zip › Fig EV2/Fig EV2A/SMAD2_membrane4_30min-6h_21.09.23_12.20.53_PUB_600.tif]

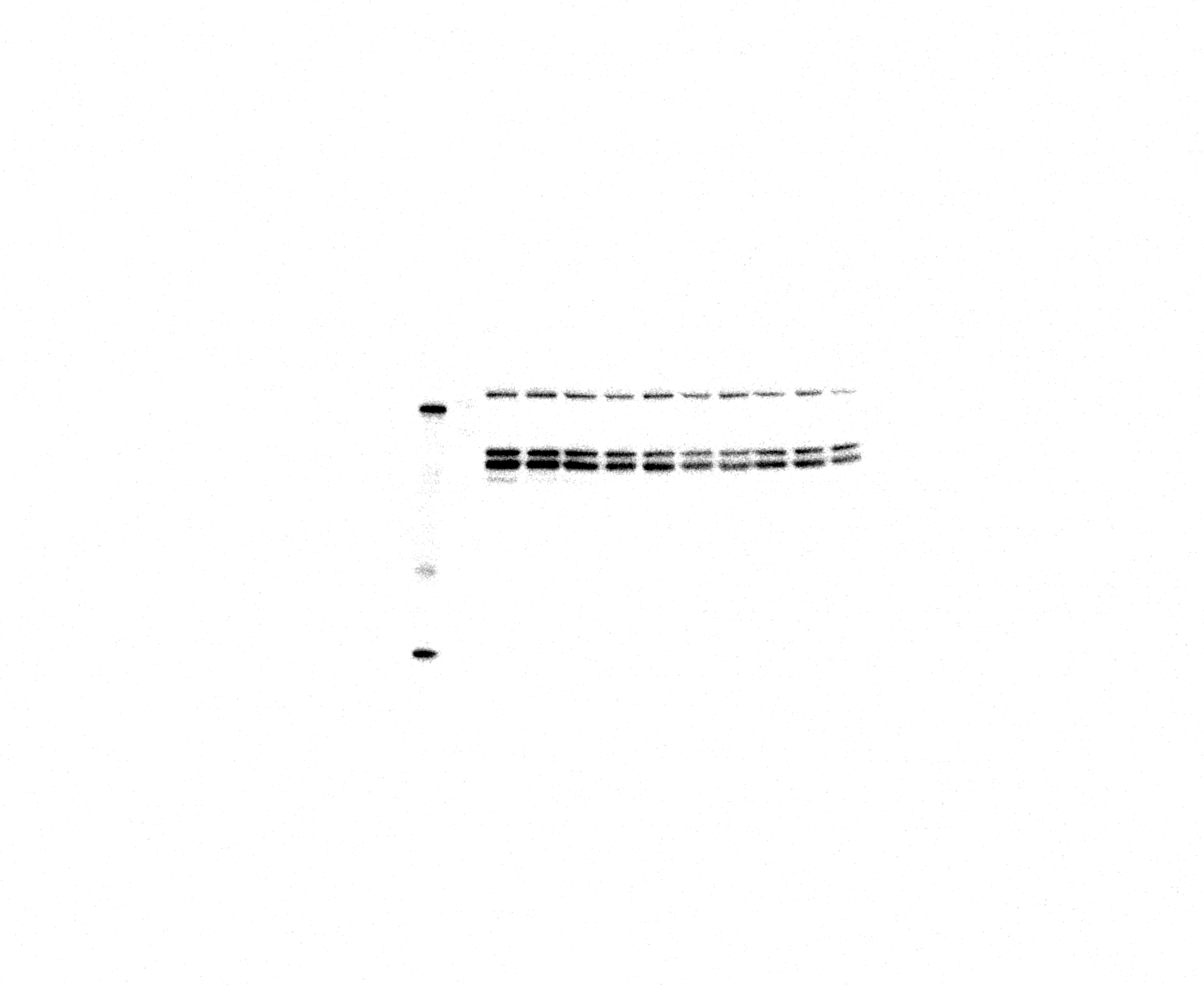

Supplement: Supplementary file 12 — Source Data Fig EV2 [file 44320_2025_116_MOESM12_ESM.zip › Fig EV2/Fig EV2A/SMAD2_membrane5_12-96h_21.09.28_11.59.18b_PUB_600.tif]

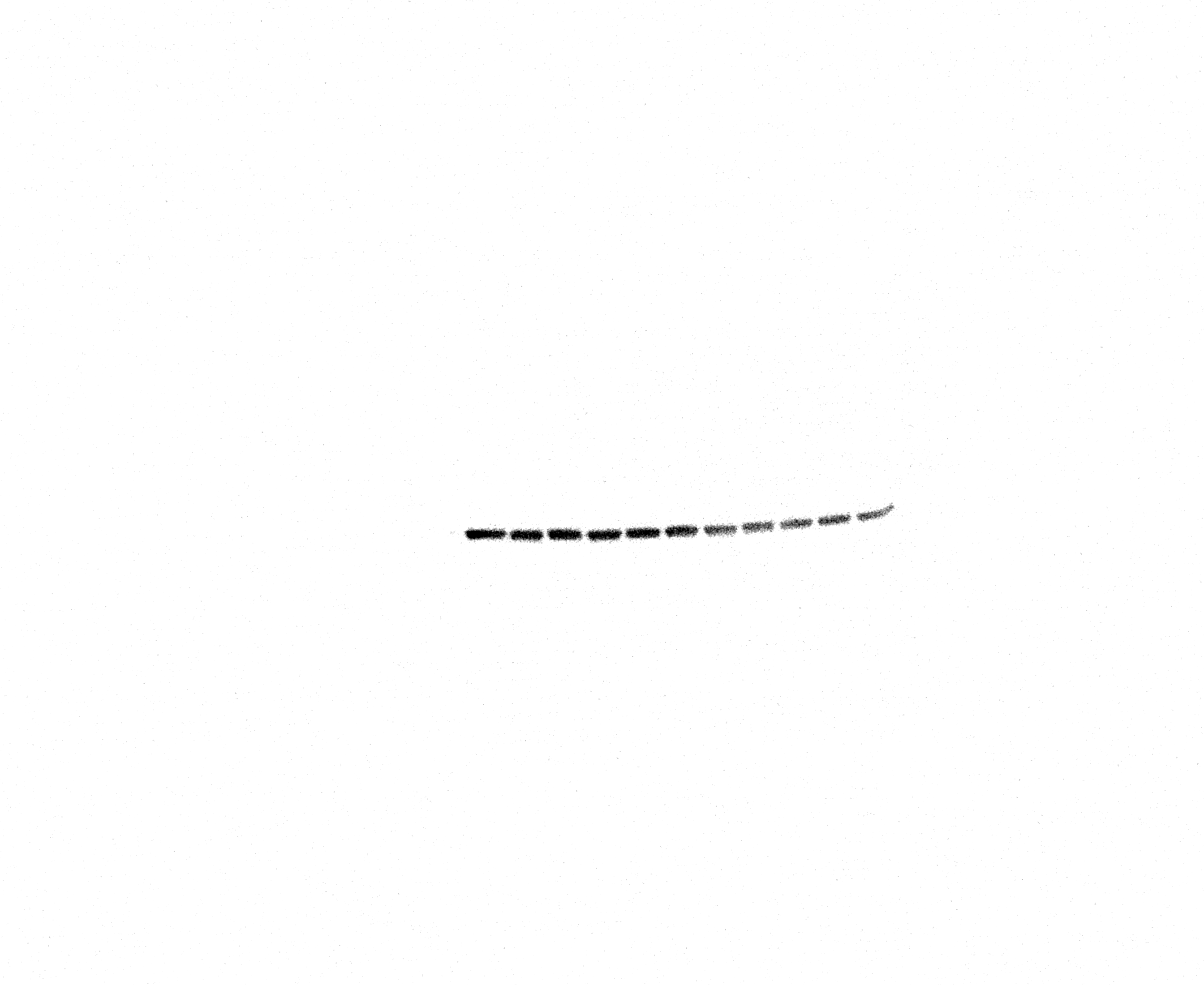

Supplement: Supplementary file 12 — Source Data Fig EV2 [file 44320_2025_116_MOESM12_ESM.zip › Fig EV2/Fig EV2A/tubulin_membrane1_21.09.23_15.15.33-q1_PUB_600.tif]

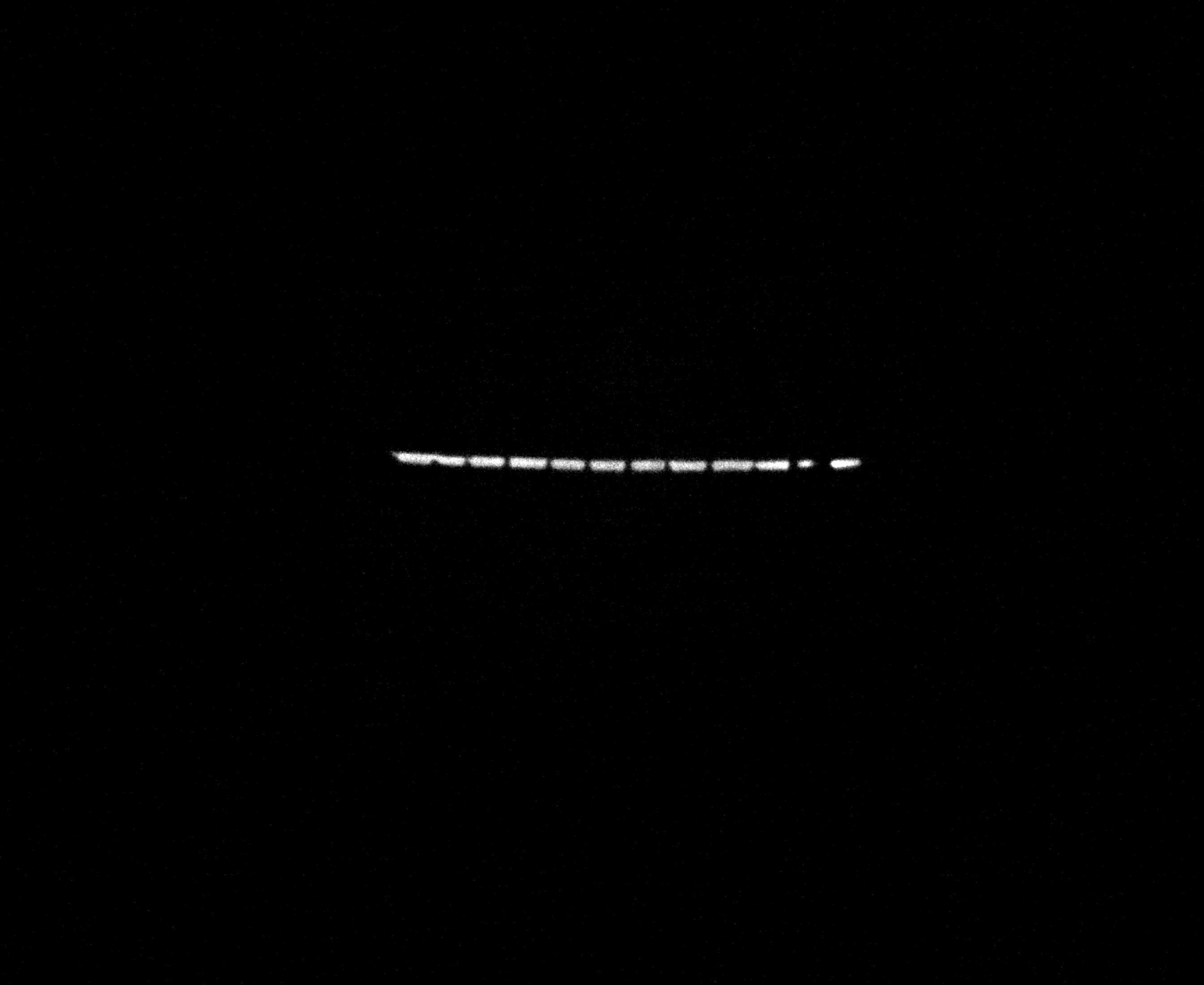

Supplement: Supplementary file 12 — Source Data Fig EV2 [file 44320_2025_116_MOESM12_ESM.zip › Fig EV2/Fig EV2A/tubulin_membrane2_21.09.23_15.25.12-2inv_PUB_600.tif]

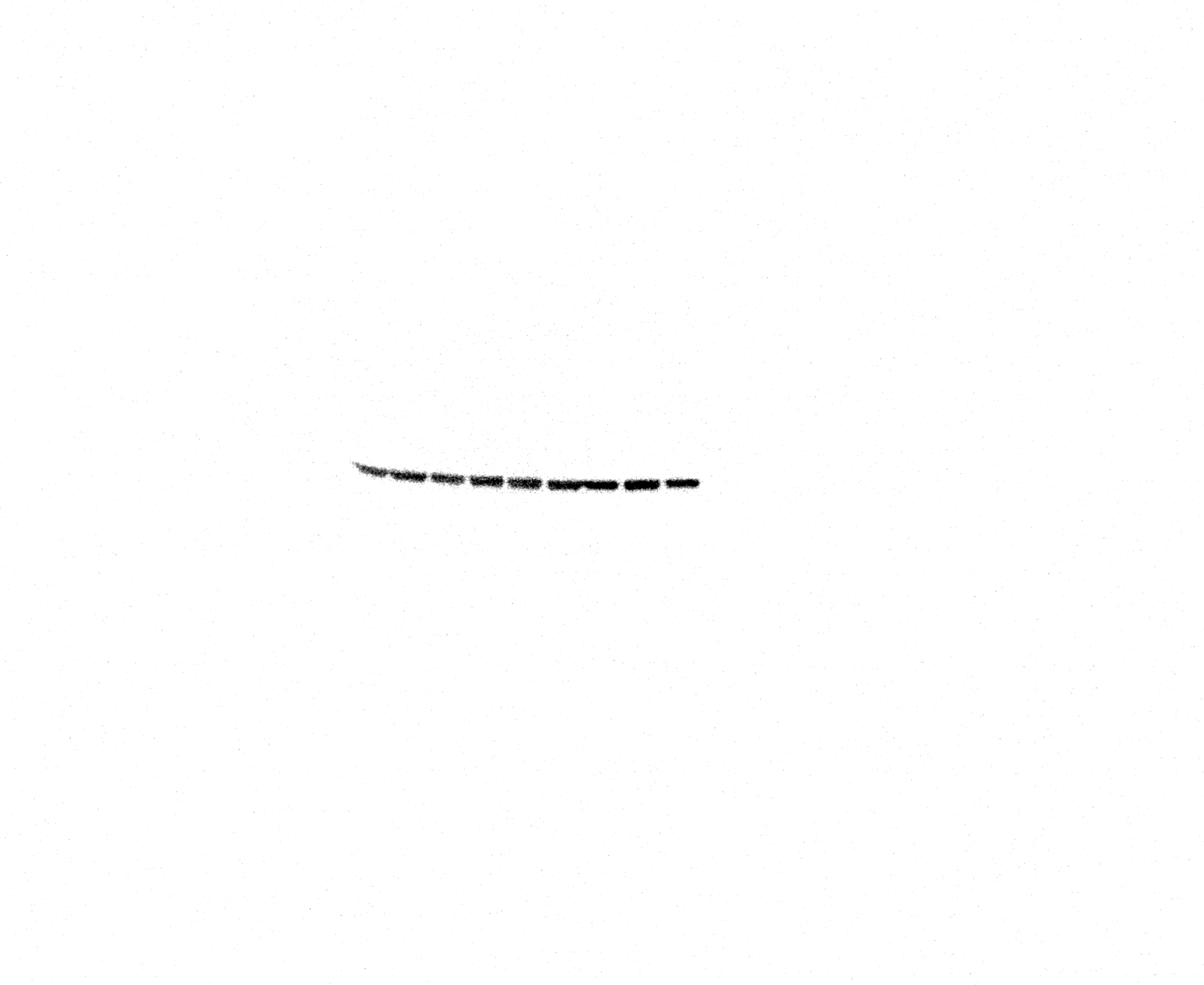

Supplement: Supplementary file 12 — Source Data Fig EV2 [file 44320_2025_116_MOESM12_ESM.zip › Fig EV2/Fig EV2A/tubulin_membrane3_21.09.23_15.19.17-w3_PUB_600 (1).tif]

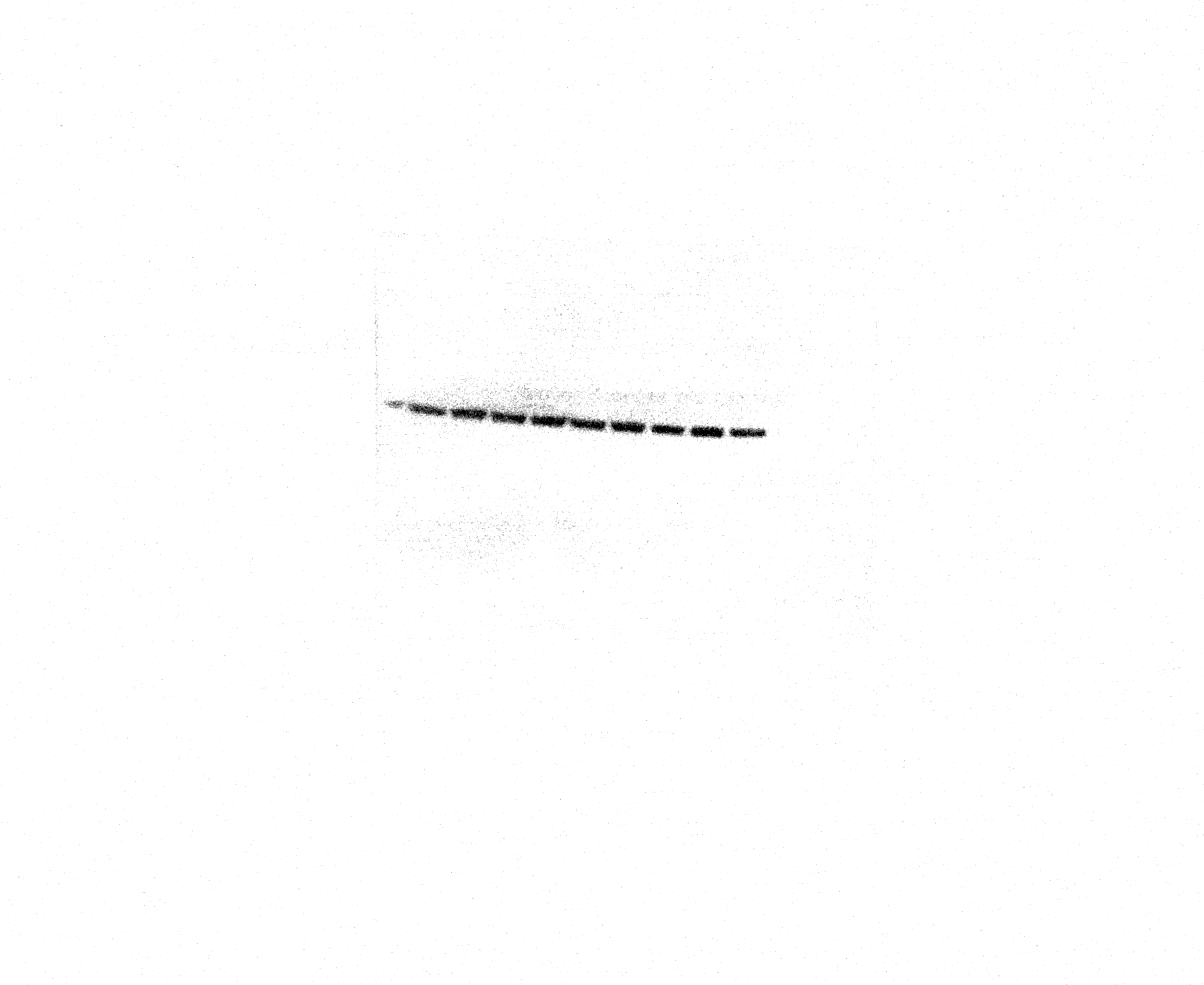

Supplement: Supplementary file 12 — Source Data Fig EV2 [file 44320_2025_116_MOESM12_ESM.zip › Fig EV2/Fig EV2A/tubulin_membrane4_21.09.23_15.21.45-4blot_PUB_600.tif]

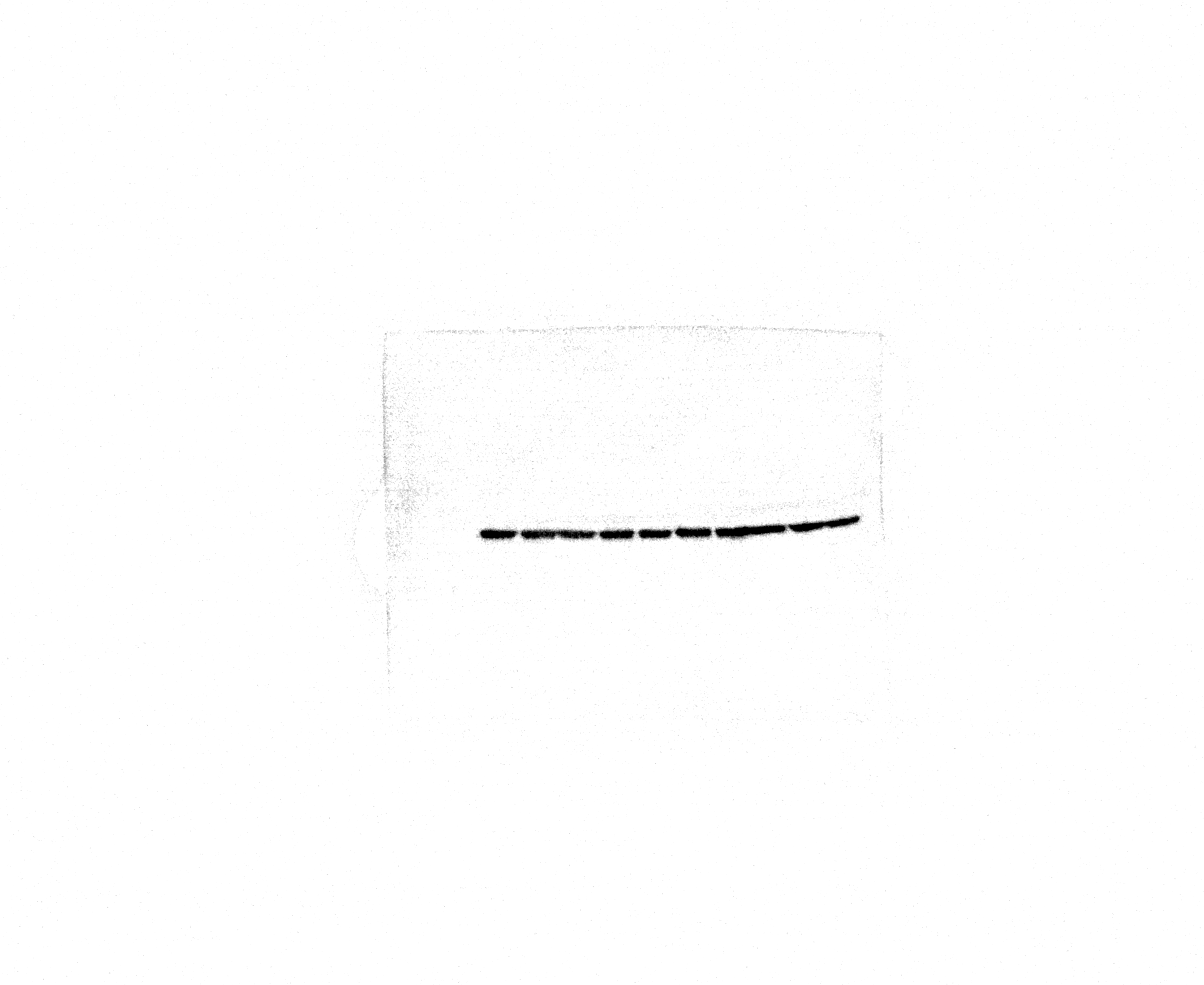

Supplement: Supplementary file 12 — Source Data Fig EV2 [file 44320_2025_116_MOESM12_ESM.zip › Fig EV2/Fig EV2A/tubulin_membrane5_21.09.28_15.38.22b_PUB_600.tif]

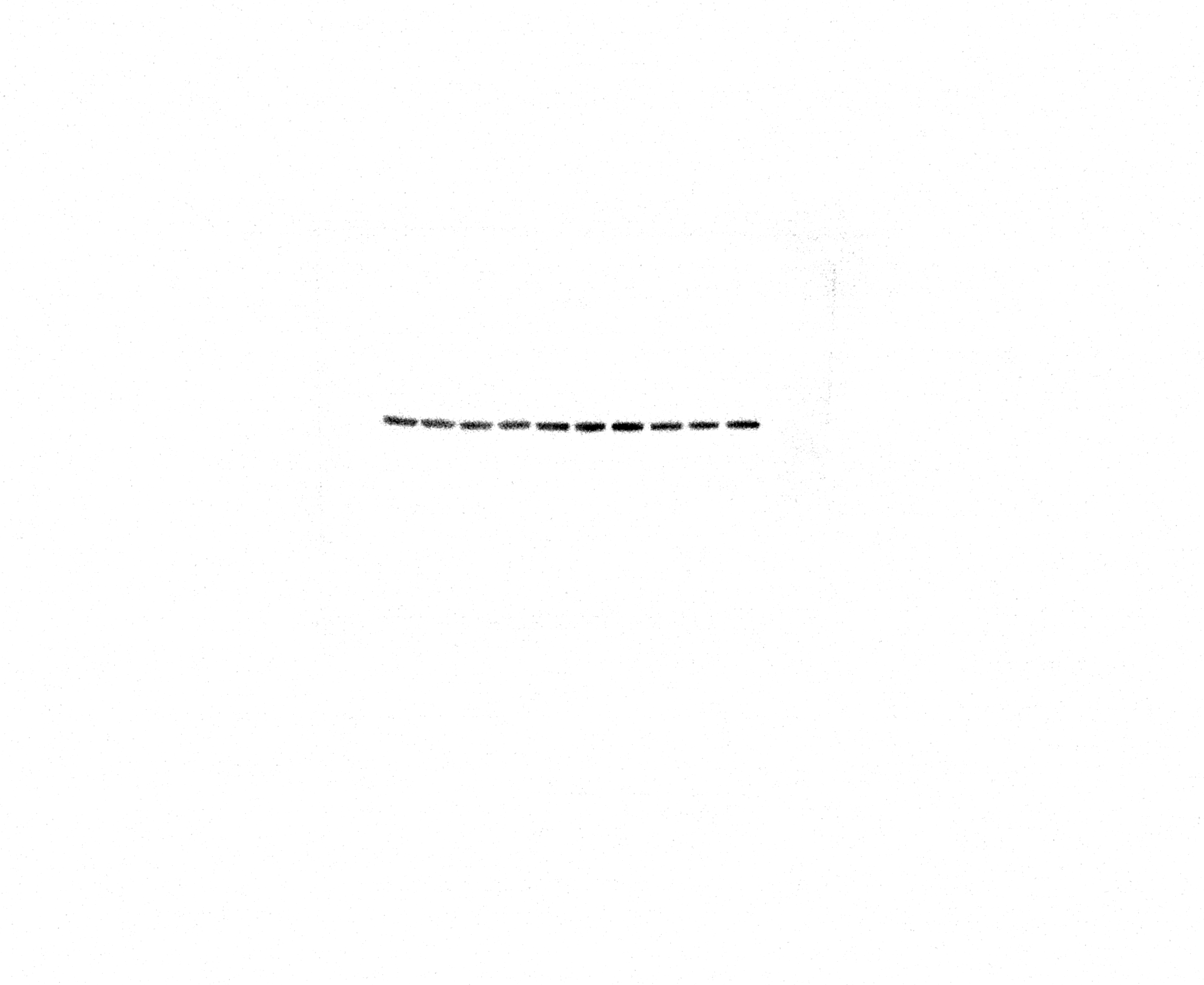

Supplement: Supplementary file 12 — Source Data Fig EV2 [file 44320_2025_116_MOESM12_ESM.zip › Fig EV2/Fig EV2A/tubulin_membrane6_21.09.28_15.32.21_PUB_600 (1).tif]

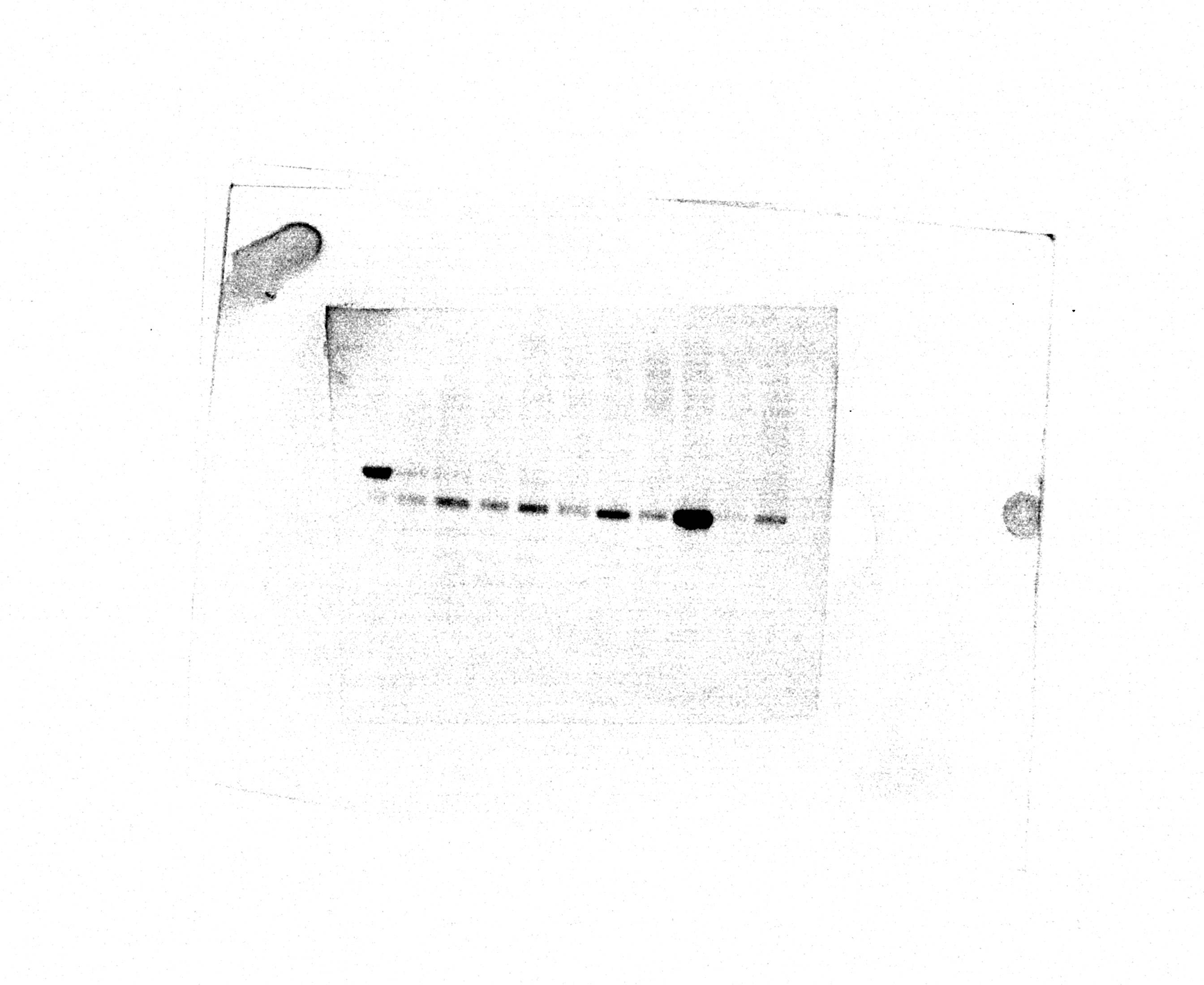

Supplement: Supplementary file 12 — Source Data Fig EV2 [file 44320_2025_116_MOESM12_ESM.zip › Fig EV2/Fig EV2B/pSMAD2_membrane1_21.11.12_14.30.00-1_PUB_600.tif]

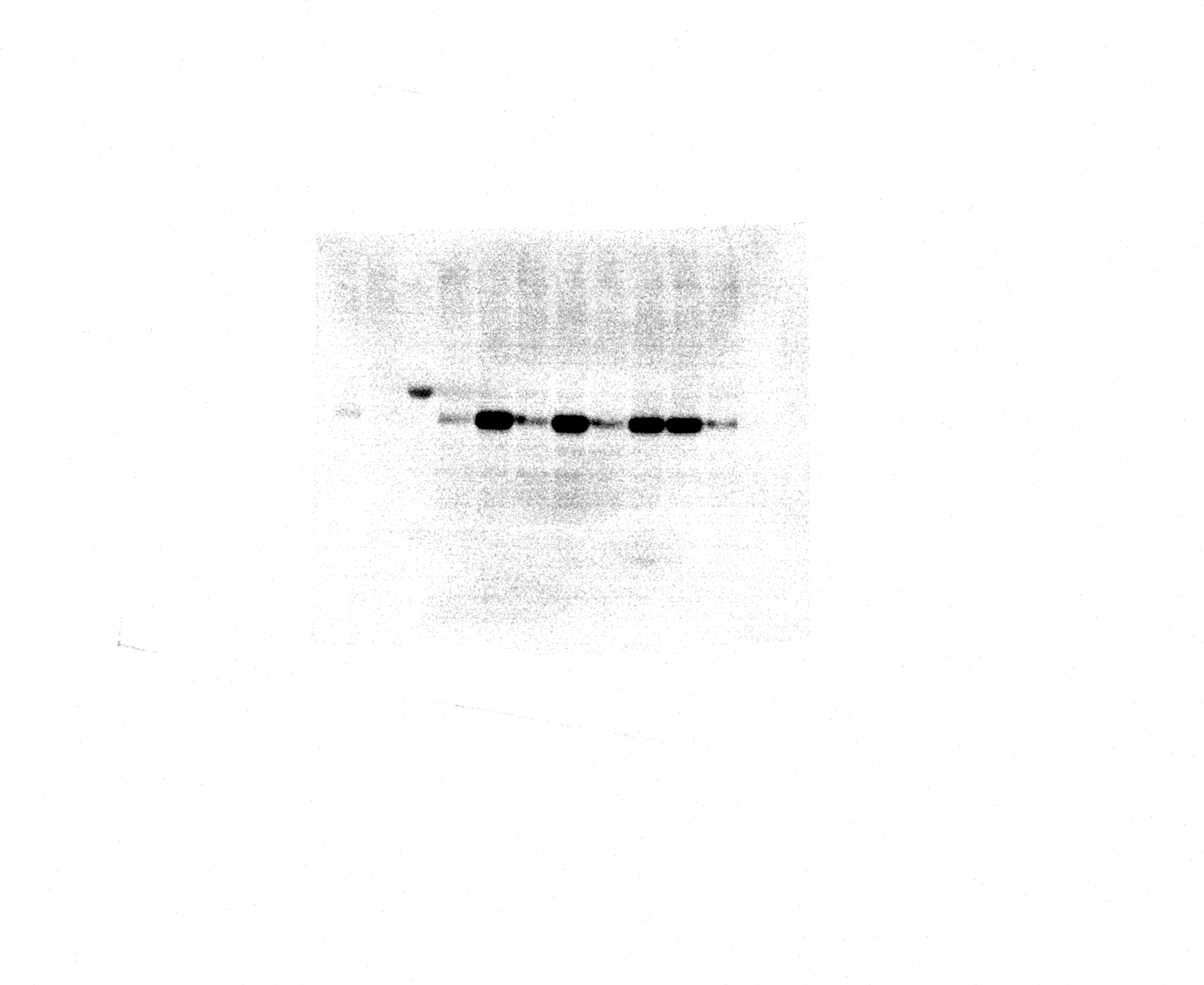

Supplement: Supplementary file 12 — Source Data Fig EV2 [file 44320_2025_116_MOESM12_ESM.zip › Fig EV2/Fig EV2B/pSMAD2_membrane3_21.11.12_14.49.22-3blot_PUB_600.tif]

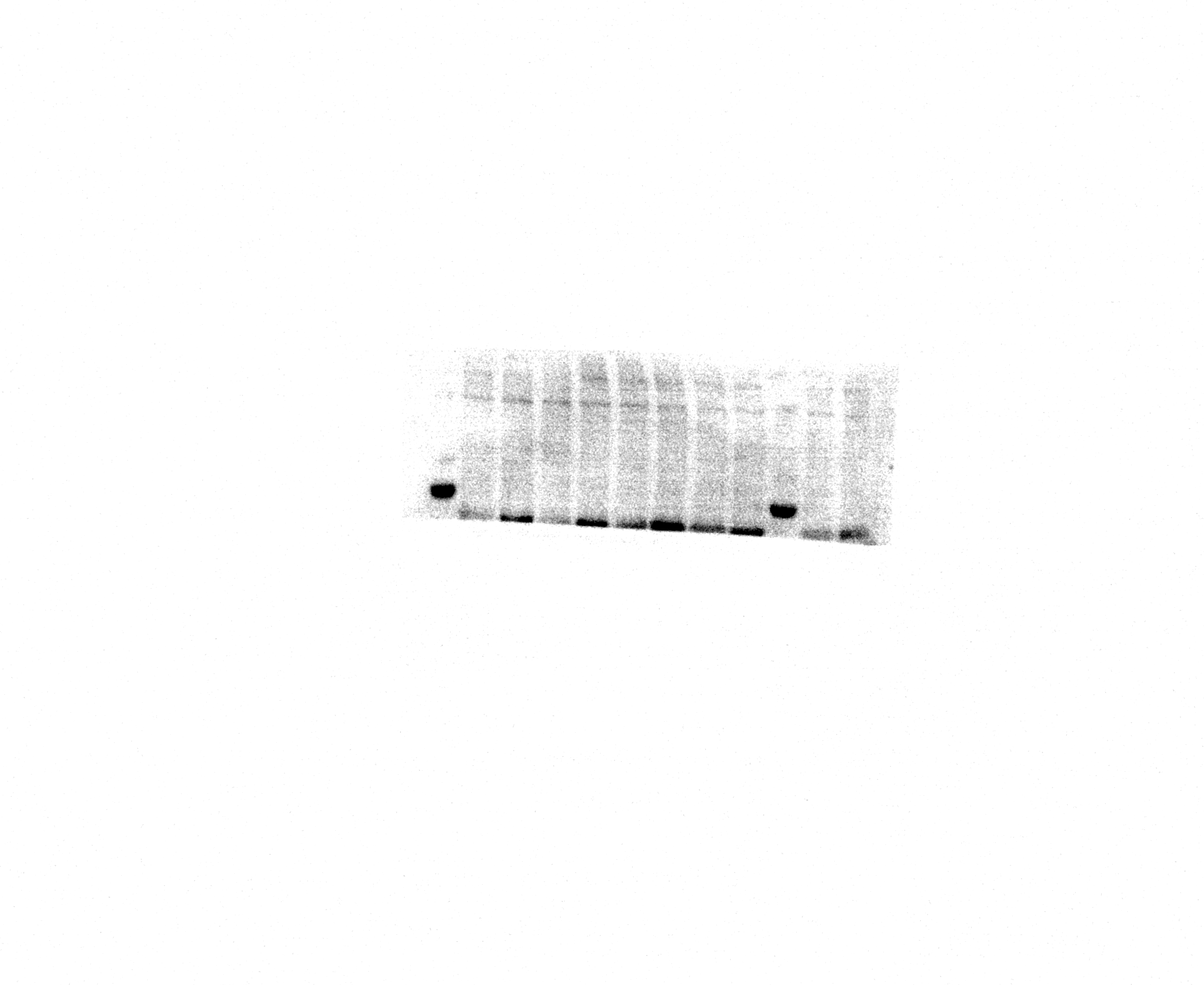

Supplement: Supplementary file 12 — Source Data Fig EV2 [file 44320_2025_116_MOESM12_ESM.zip › Fig EV2/Fig EV2B/pSMAD2_membrane5_21.11.16_12.02.20-5-blot_PUB_600.tif]

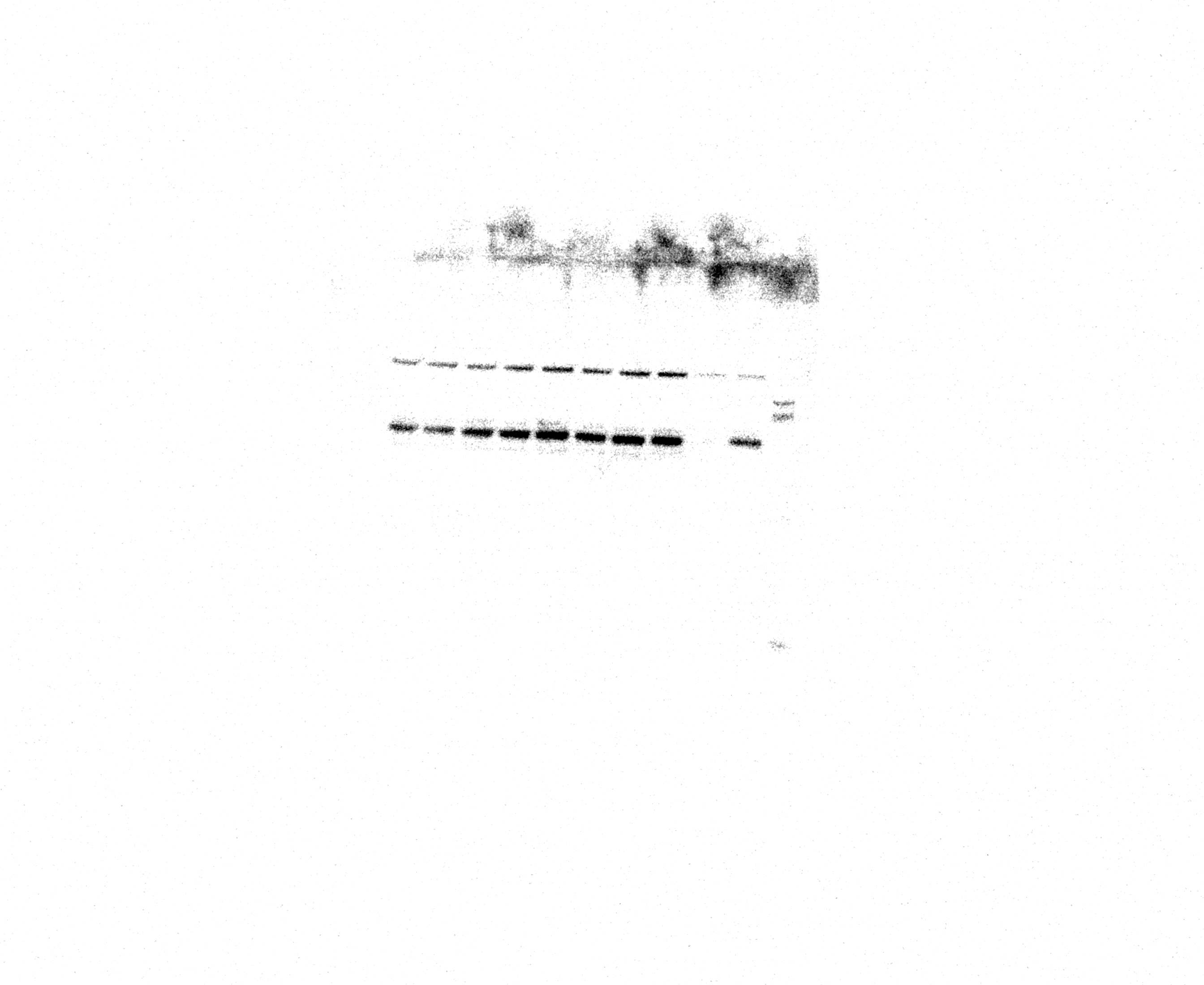

Supplement: Supplementary file 12 — Source Data Fig EV2 [file 44320_2025_116_MOESM12_ESM.zip › Fig EV2/Fig EV2B/SMAD2_membrane2_21.11.12_14.36.47-2blo_PUB_600.tif]

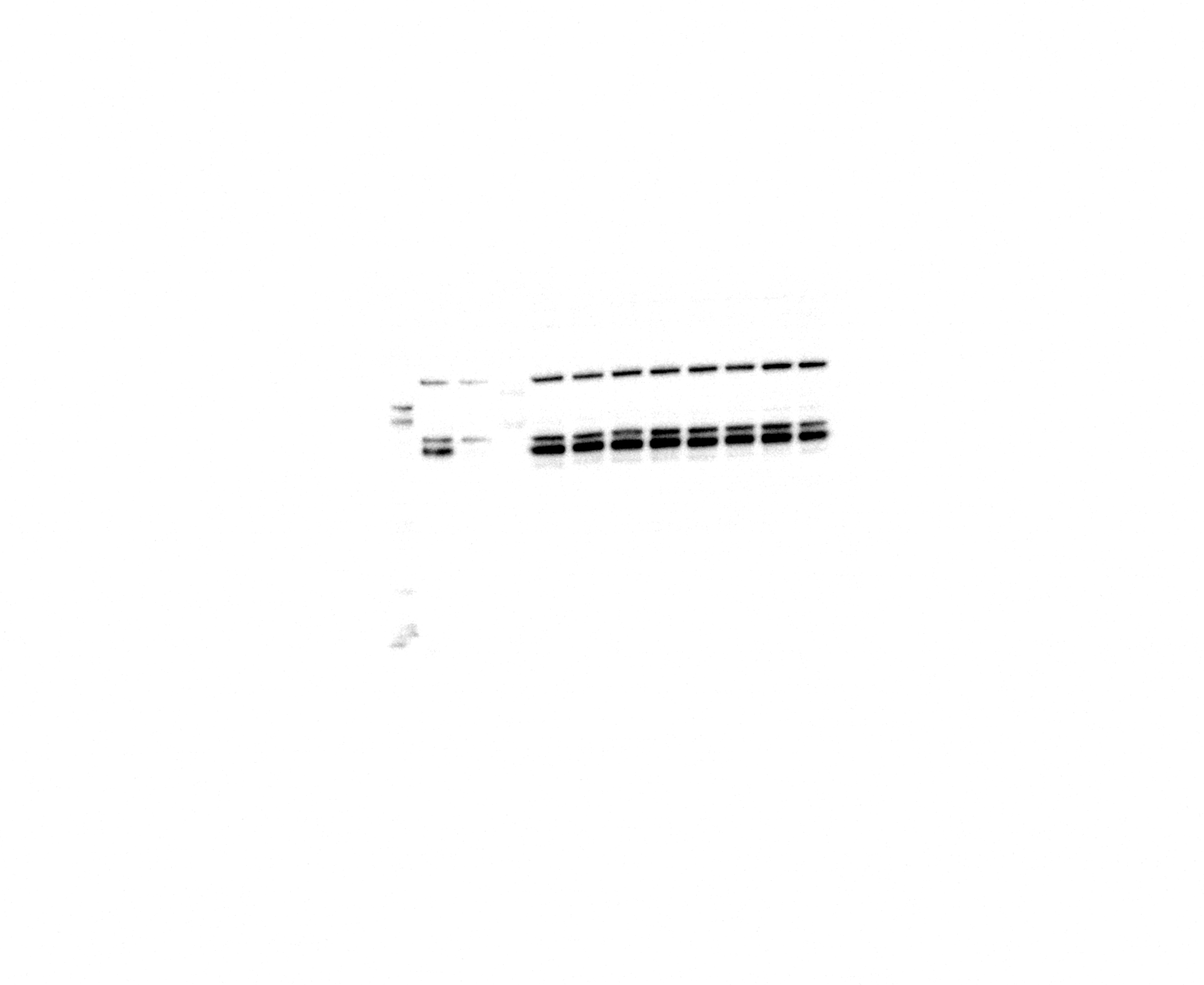

Supplement: Supplementary file 12 — Source Data Fig EV2 [file 44320_2025_116_MOESM12_ESM.zip › Fig EV2/Fig EV2B/SMAD2_membrane4_21.11.12_14.34.13-4_PUB_600.tif]

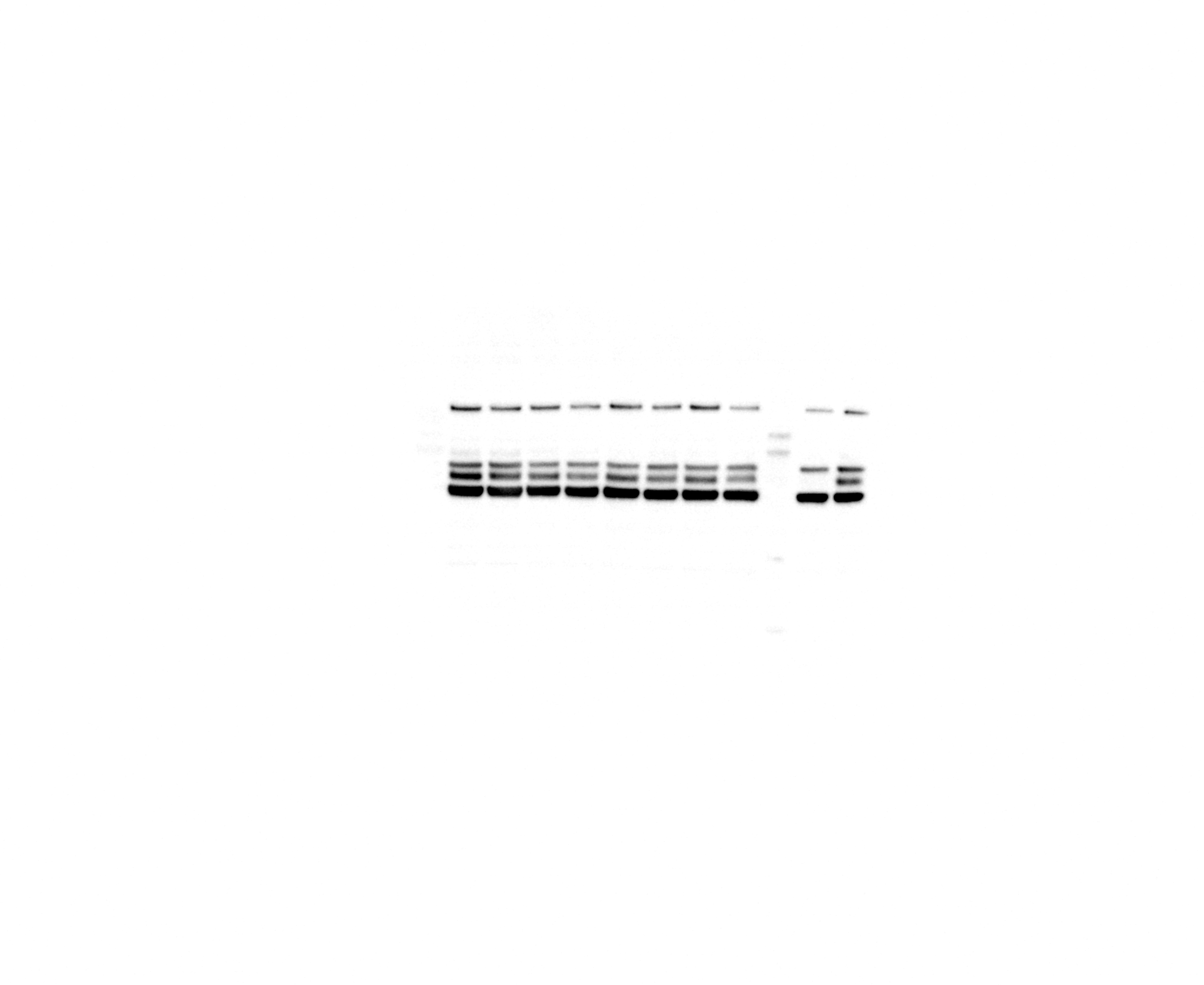

Supplement: Supplementary file 12 — Source Data Fig EV2 [file 44320_2025_116_MOESM12_ESM.zip › Fig EV2/Fig EV2B/SMAD2_membrane6_21.11.16_11.20.47_6_PUB_600.tif]

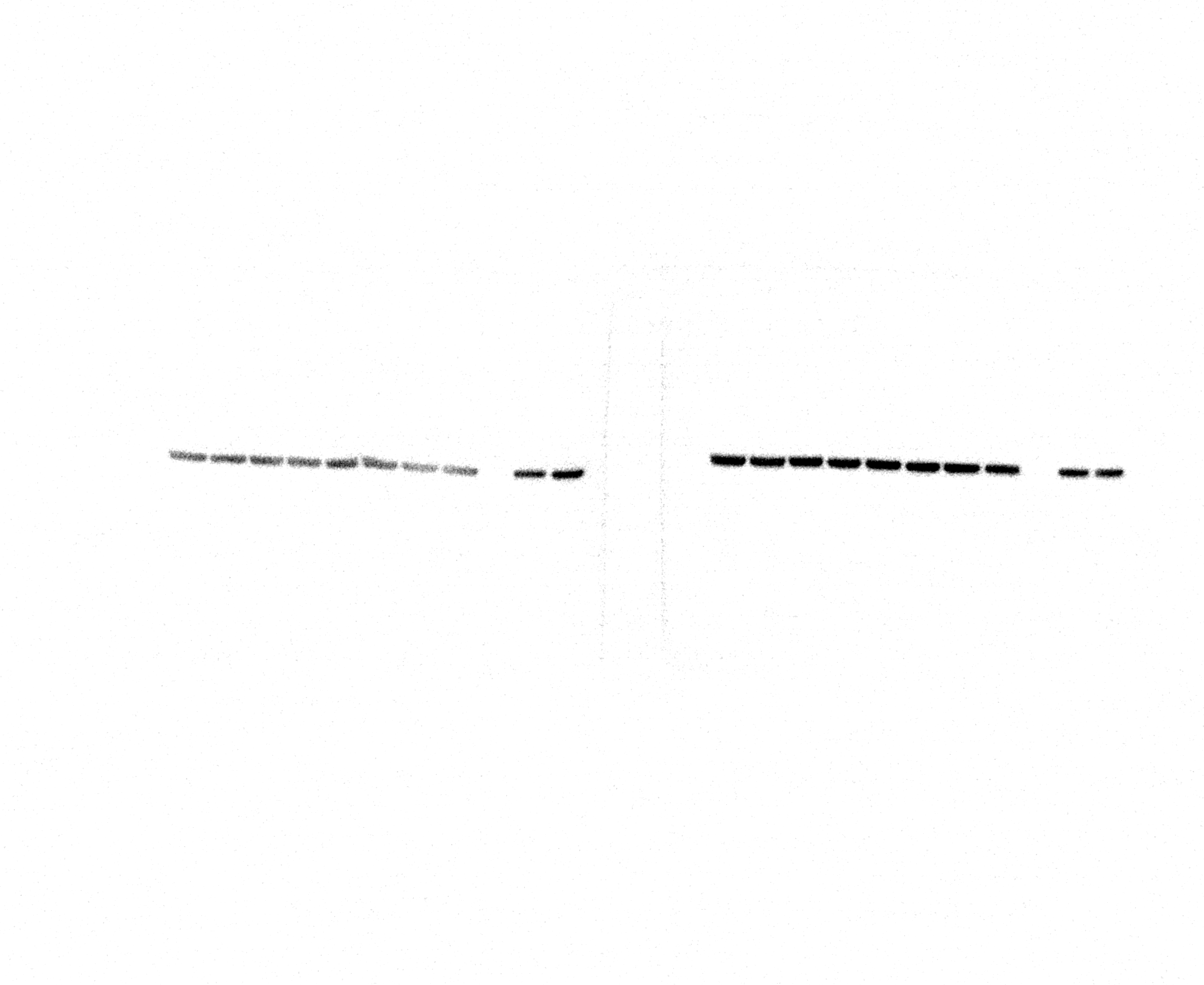

Supplement: Supplementary file 12 — Source Data Fig EV2 [file 44320_2025_116_MOESM12_ESM.zip › Fig EV2/Fig EV2B/tubilin_membrane5and621.11.15_16.17.57_PUB_600.tif]

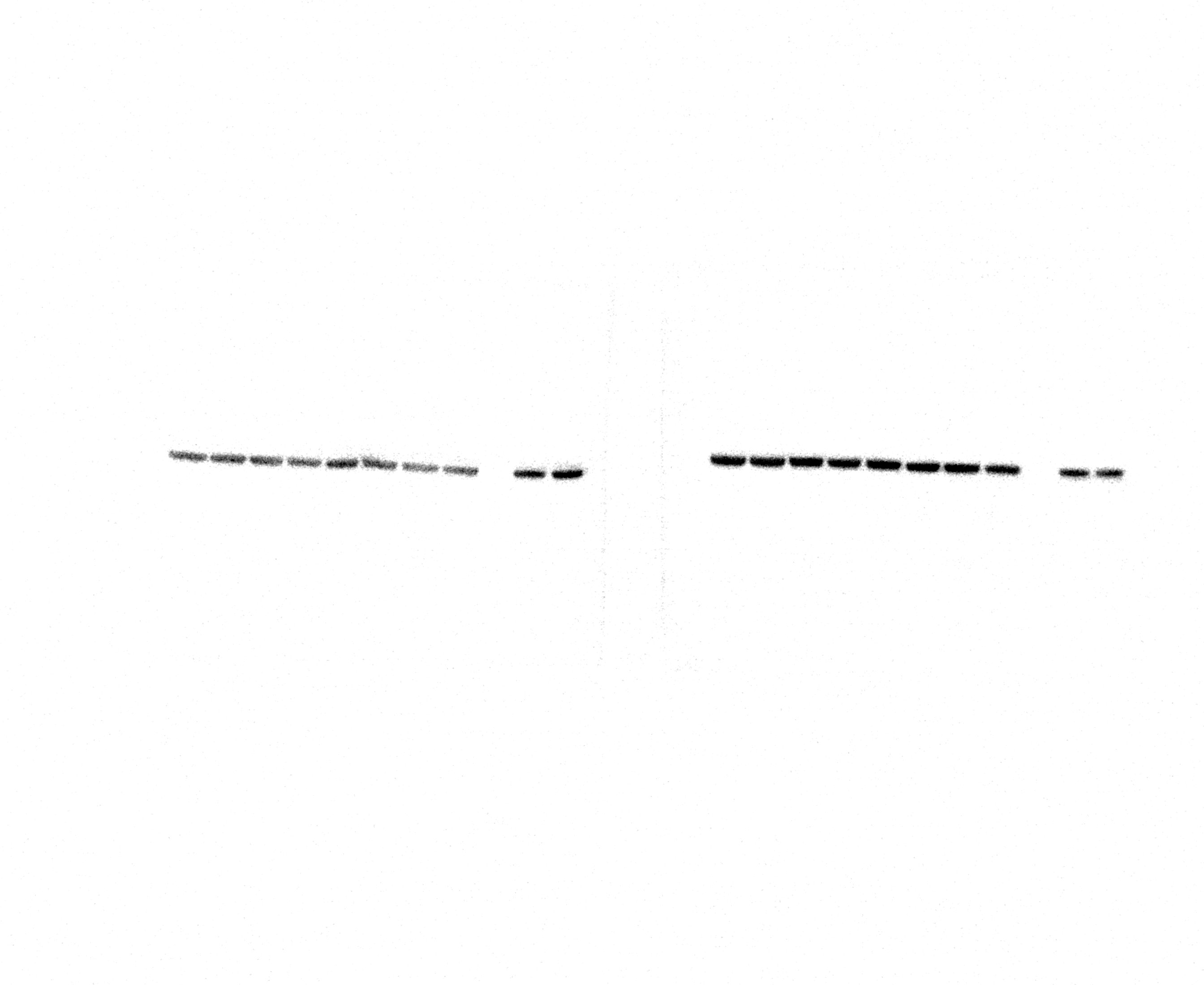

Supplement: Supplementary file 12 — Source Data Fig EV2 [file 44320_2025_116_MOESM12_ESM.zip › Fig EV2/Fig EV2B/tubilin_membrane5and6_21.11.15_16.16.11blot_PUB_600.tif]

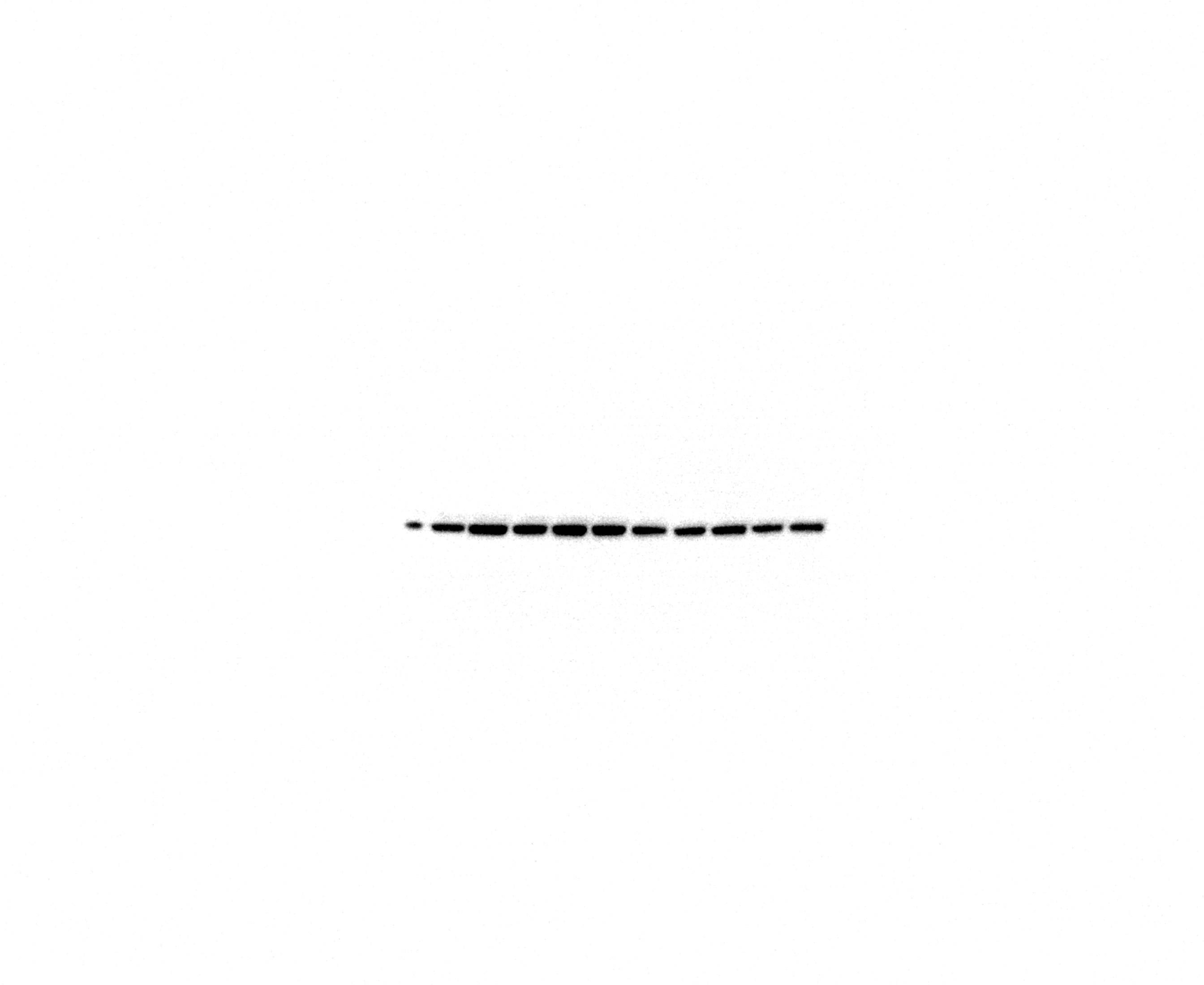

Supplement: Supplementary file 12 — Source Data Fig EV2 [file 44320_2025_116_MOESM12_ESM.zip › Fig EV2/Fig EV2B/tubulin_membrane1_21.11.12_18.08.29_1_PUB_600.tif]

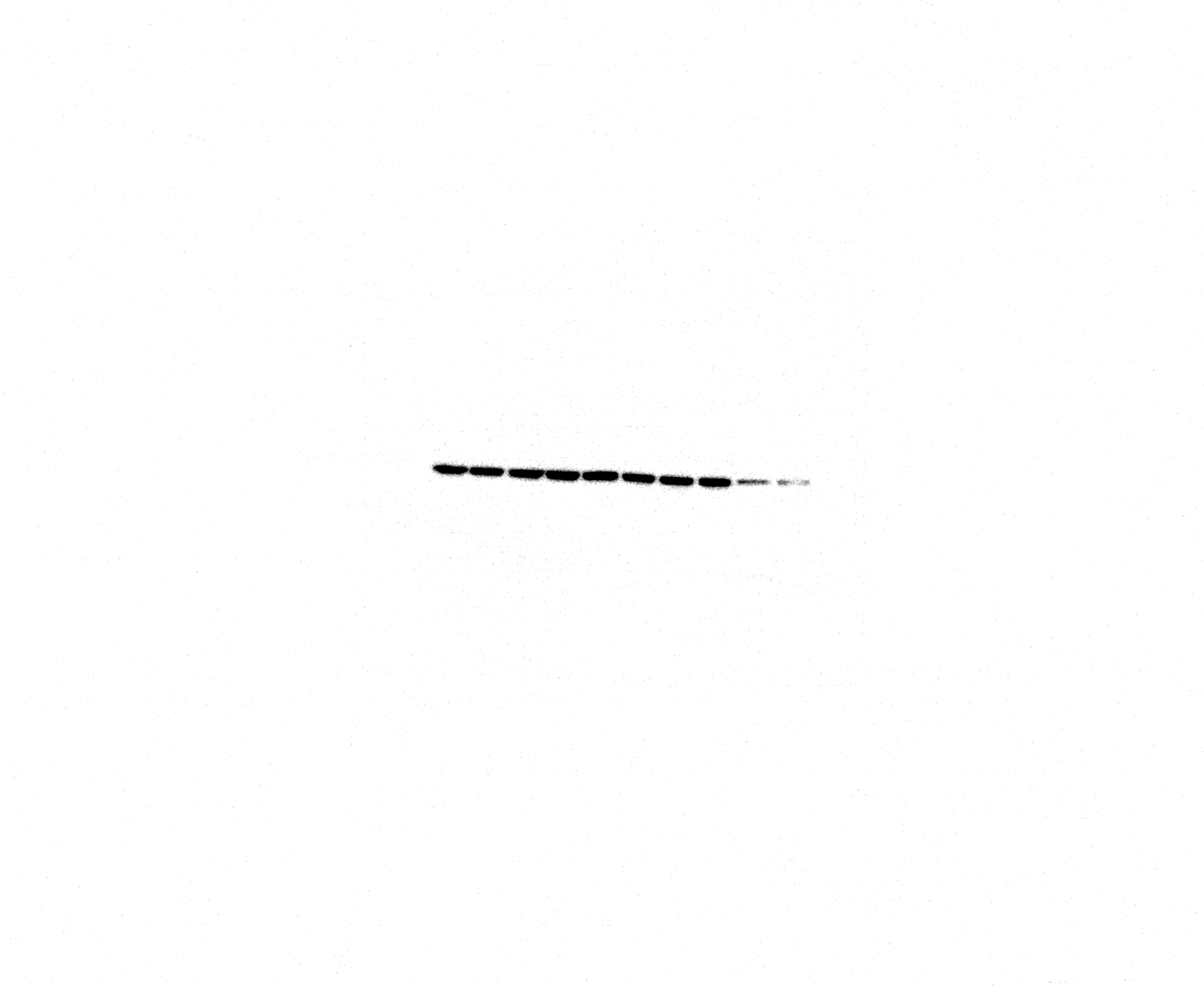

Supplement: Supplementary file 12 — Source Data Fig EV2 [file 44320_2025_116_MOESM12_ESM.zip › Fig EV2/Fig EV2B/tubulin_membrane2_21.11.12_17.59.48_2_PUB_600.tif]

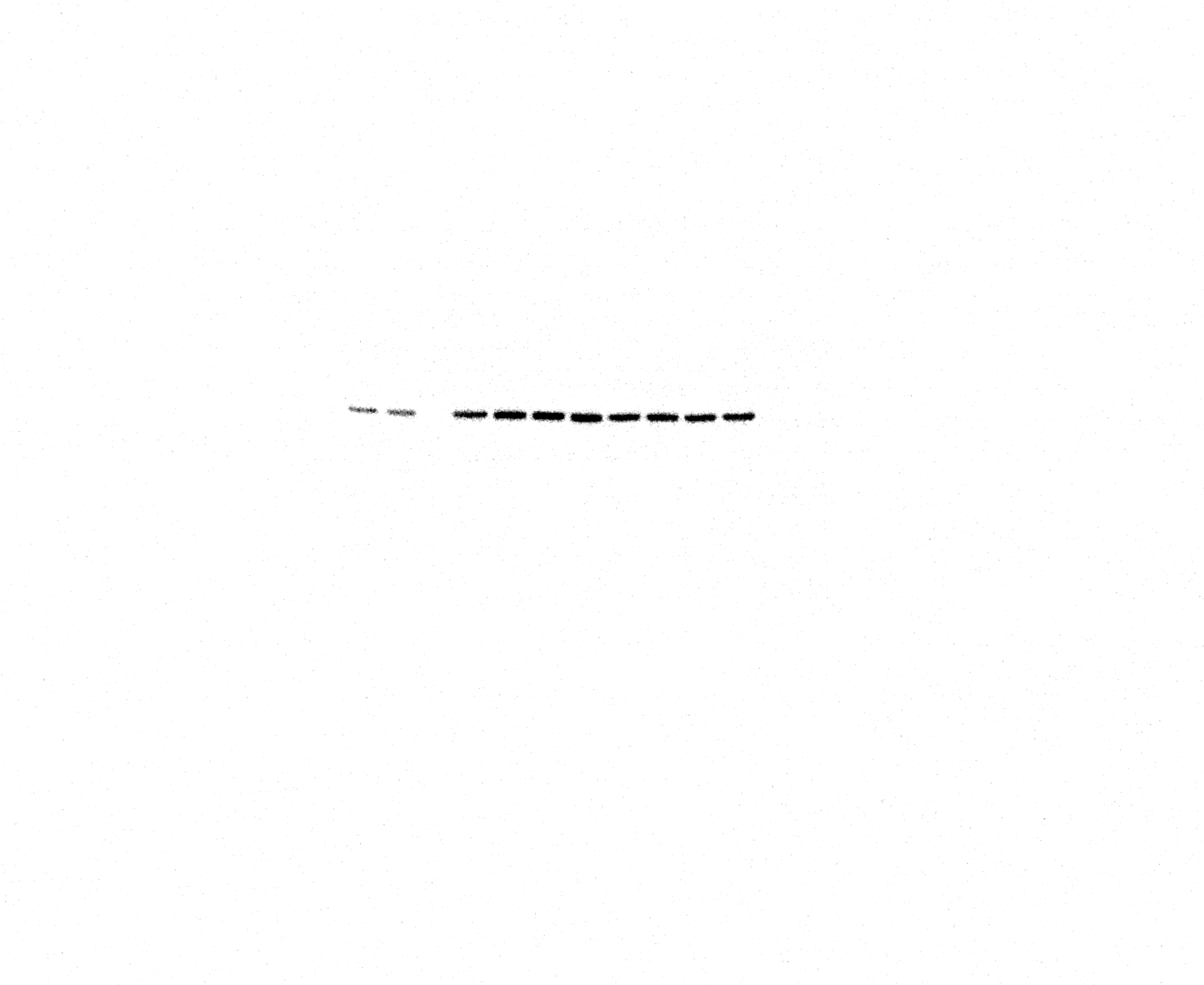

Supplement: Supplementary file 12 — Source Data Fig EV2 [file 44320_2025_116_MOESM12_ESM.zip › Fig EV2/Fig EV2B/tubulin_membrane3_21.11.12_18.02.52_3_PUB_600.tif]

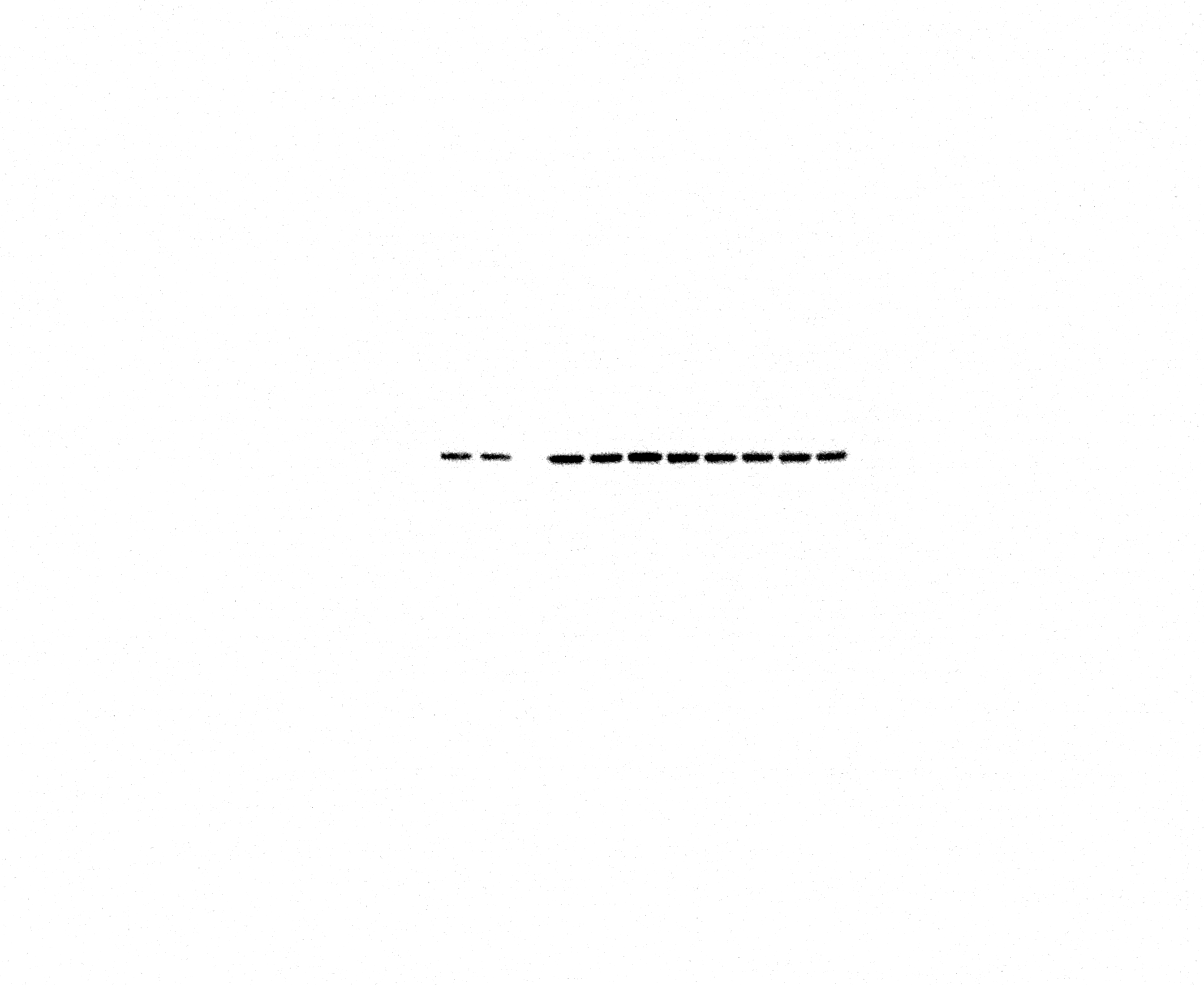

Supplement: Supplementary file 12 — Source Data Fig EV2 [file 44320_2025_116_MOESM12_ESM.zip › Fig EV2/Fig EV2B/tubulin_membrane4_21.11.12_18.07.06_4_PUB_600.tif]
