# Supplementary material for: Dynamic multi-omics and mechanistic modeling approach uncovers novel mechanisms of kidney fibrosis progression
Source: Mol Syst Biol. 2025 Jun 5;21(8):1030–65. doi: 10.1038/s44320-025-00116-2 (PMC12322177; doi:10.1038/s44320-025-00116-2)
Supplement: Supplementary file 13 — Expanded View Figures [file 44320_2025_116_MOESM13_ESM.pdf]

Expanded View Figures

Figure EV1. Time course of COL1 expression and cytoskeletal changes in response to TGF-β treatment.

(**A**) Widefield microscopy images showing COL1 (top rows) and nuclear (Hoechst, bottom rows) staining in control and TGF-β-treated conditions at 0, 12, 24, 48, 72, and 96 h. (**B**) Quantification of COL1 expression per cell over time. Cells were cultured in four biological replicates (**A–C**) ± TGF-β for 0-96 h. Data points represent individual images, color-coded for control (gray) and TGF-β (orange) conditions. *Y* axis shows normalized COL1 intensity per cell, *x* axis shows nuclei count per image. Images with less than 20 nuclei were excluded from the analysis. (**C**) Sum Z-projections of confocal microscopy images displaying F-actin (phalloidin, top rows) and nuclear (bottom rows) staining in control and TGF-β treated conditions at the same time points as in (**A**). In panels (**A**, **C**), the control condition is shown in the lower half (gray background), while the TGF-β-treated condition is presented in the upper half (orange background). Scale bar = 100 µm. Yellow arrowheads indicate specific features of interest (fibrillar collagen or F-actin, respectively), while hollow arrowheads indicate autofluorescence of cells.

© The Author(s)

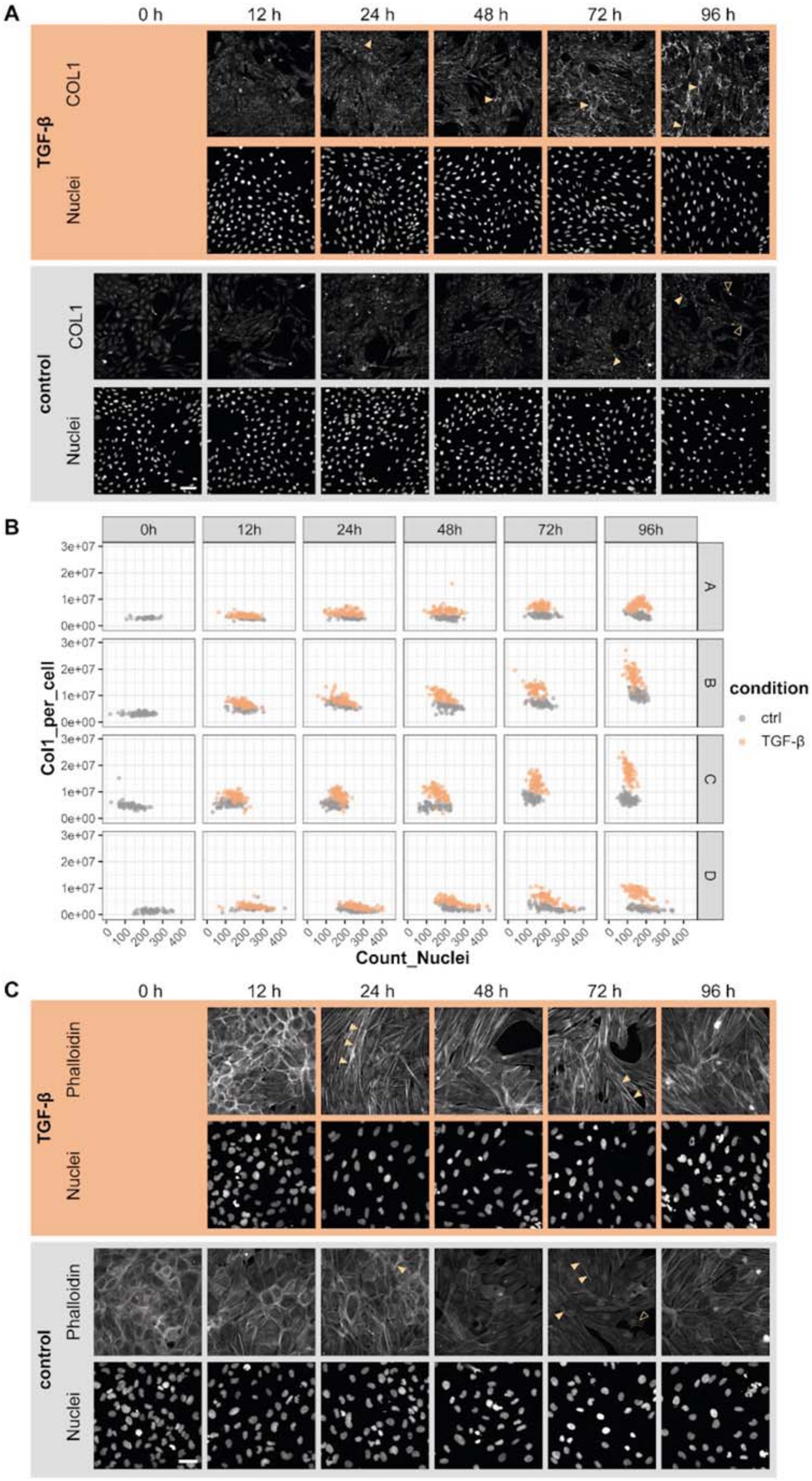
A
0 h
12 h
24 h
48 h
72 h
96 h
TGF-β
COL1
Nuclei
control
COL1
Nuclei
B
0h
12h
24h
48h
72h
96h
A
B
C
D
Col1_per_cell
Count_Nuclei
condition
ctrl
TGF-β
C
0 h
12 h
24 h
48 h
72 h
96 h
TGF-β
Phalloidin
Nuclei
control
Phalloidin
Nuclei

 © The Author(s)

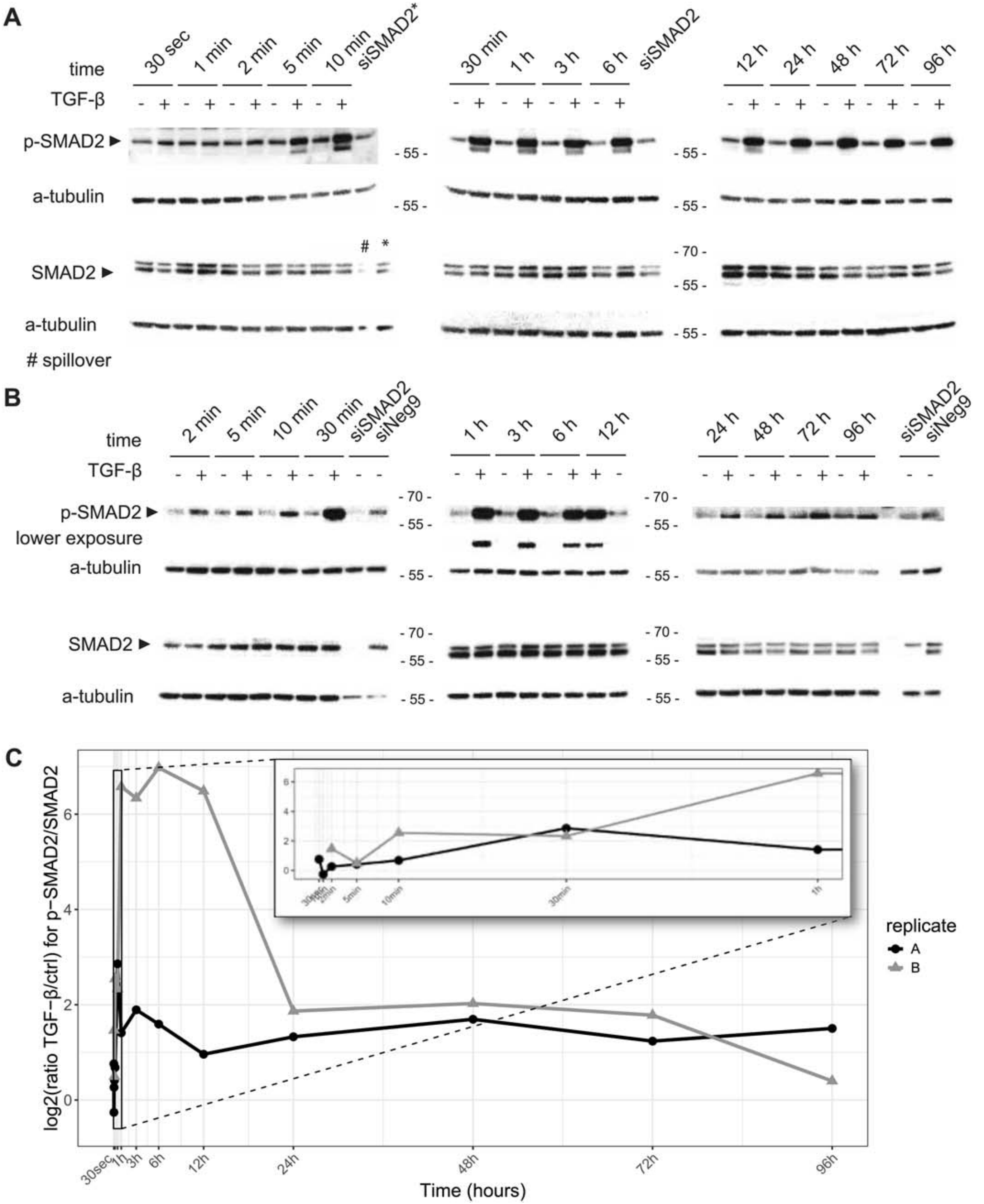
A
time
30 sec
1 min
2 min
5 min
10 min
siSMAD2*
30 min
1 h
3 h
6 h
siSMAD2
12 h
24 h
48 h
72 h
96 h
TGF-β
p-SMAD2
a-tubulin
SMAD2
spillover
- 55 -
- 70 -
B
siNeg9
lower exposure
C
log2(ratio TGF−β/ctrl) for p−SMAD2/SMAD2
Time (hours)
replicate
A
B

Figure EV2. TGF-β-induced SMAD2 phosphorylation dynamics.

(**A**) Western blot analysis of phosphorylated SMAD2 (p-SMAD2), total SMAD2, and α-tubulin (loading control) in response to TGF-β treatment over time. Time points range from 30 s to 96 h. siSMAD2 condition is included as a control. A spill-over has been labeled with '#', the correct corresponding band and position has been labeled with '*'. (**B**) Western blot analysis of a biological replicate, similar to (**A**). Time points range from 2 min to 96 h. siSMAD2 and siNeg9 conditions are included as controls. (**C**) Quantification of p-SMAD2 levels normalized to total SMAD2 from (**A**, **B**). Each of the measurements was normalized to the corresponding loading control before calculating the log2 fold change between TGF-β and control-treated samples. The graph shows the log2 ratio of TGF-β/ctrl for p-SMAD2/SMAD2 across all time points. Two replicates (**A**, **B**) are represented. Source data are available online for this figure.

 © The Author(s)

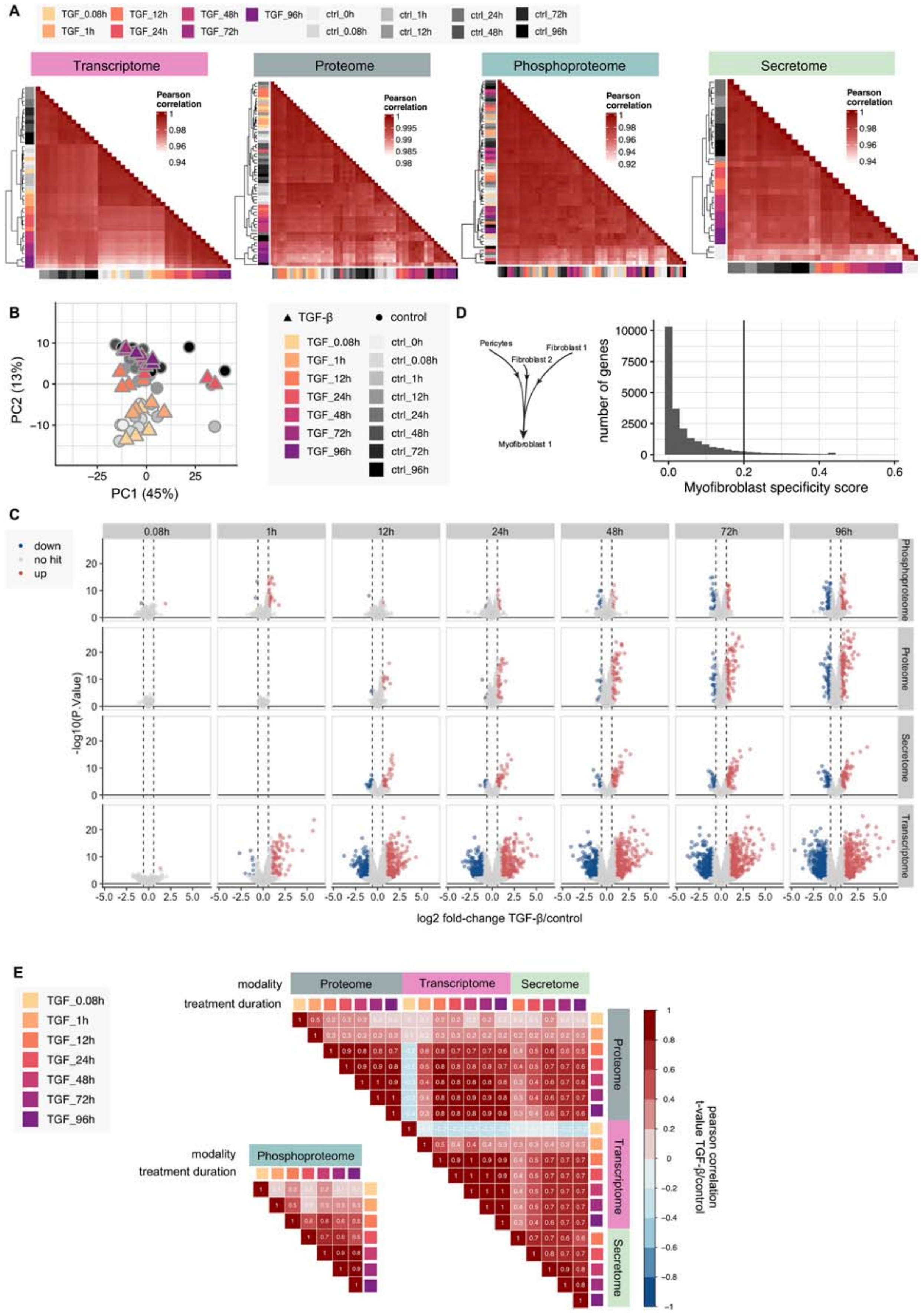
A
Transcriptome
Proteome
Phosphoproteome
Secretome
Pearson correlation
B
PC1 (45%)
PC2 (13%)
TGF-β
control
C
down
no hit
up
-log10(P.Value)
log2 fold-change TGF-β/control
D
Pericytes
Fibroblast 1
Fibroblast 2
Myofibroblast 1
number of genes
Myofibroblast specificity score
E
modality
treatment duration
pearson correlation t-value TGF-β/control

◀ **Figure EV3. Reproducible multi-omic characterisation of TGF-β-induced effects over time.**

(**A**) Heatmap showing Pearson correlation coefficients between individual biological replicates, calculated using TMT reporter intensities (proteomics/phosphoproteomics) or gene counts (transcriptomic). Each row/column represents a single biological replicate. The color gradients indicate the time points for TGF-β-treated (yellow to purple) and control (greyscale) samples. (**B**) PCA scatter plot for phosphoproteomics data (PC1 vs PC2). Triangles represent TGF-β-treated samples, circles represent controls. Color gradients indicate time points as in (A). (**C**) Results of the differential expression analysis per time point and omics modality. Colors indicate transcripts and proteins significantly deregulated in abundance upon TGF-β stimulation in comparison to control samples (limma *t* test, adjusted *P* value < 0.05, absolute log2 fold change > log2(2) for transcripts, absolute log2 fold change > log2 (1.5) for proteins). Sample sizes are as in Fig. 1F. (**D**) Specificity score distribution for myofibroblasts from CKD patients (Human PDGFRβ+ level 2) retrieved from Kuppe et al, 2021 (Kuppe et al, 2021). The black line indicates the chosen specificity cutoff for comparisons to this study (0.2). (**E**) Heatmap showing Pearson correlations between time points and omics modalities for genes with a significantly deregulated transcript or protein in at least one modality (limma, adjusted *P* value < 0.05, absolute log2 fold change > log2(2) for transcripts, absolute log2 fold change > log2(1.5) for proteins, $n = 242$ and $n = 780$ for phosphopeitdes), filtered for genes detected in all modalities.

 © The Author(s)

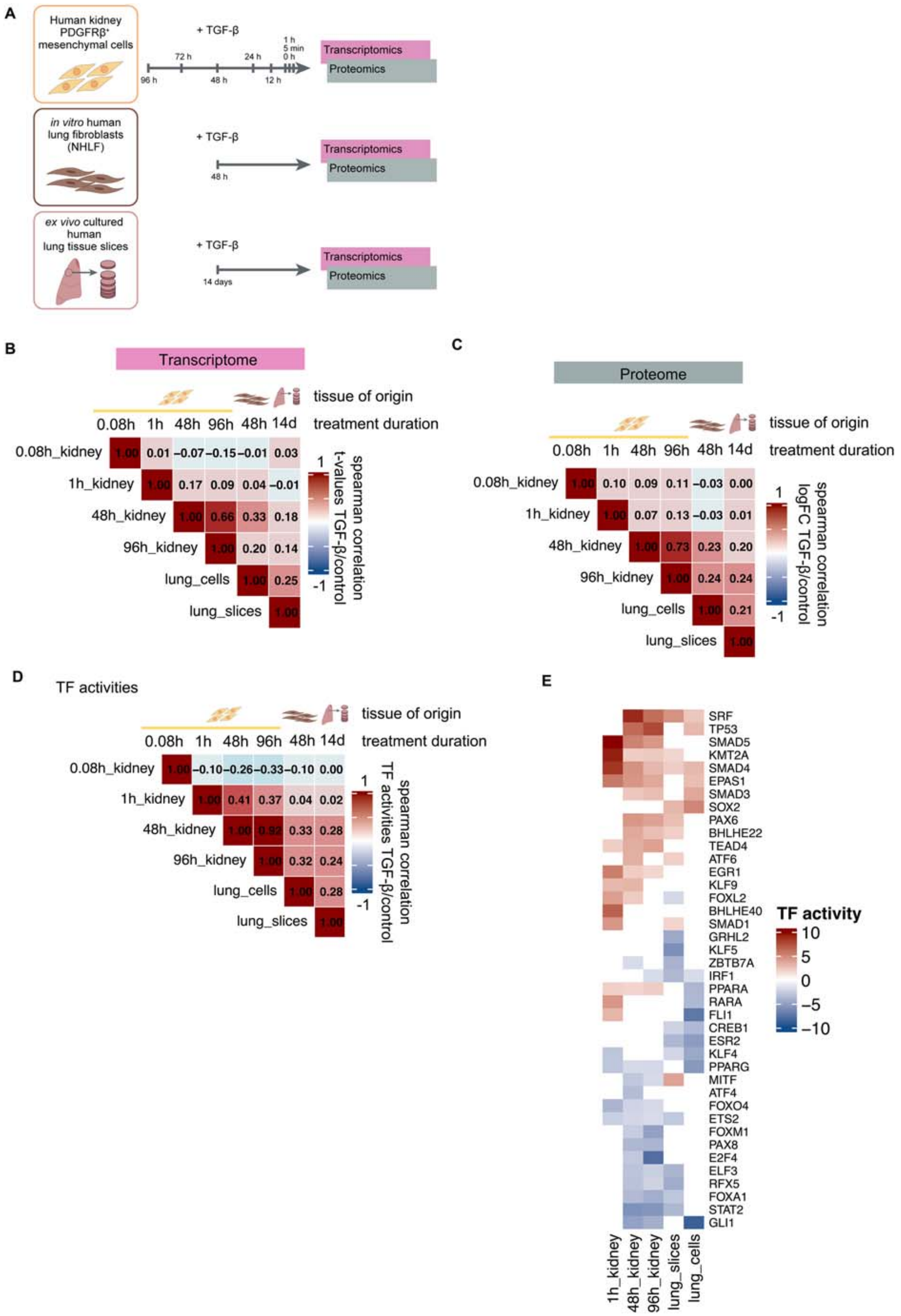
A
Human kidney PDGFRβ+ mesenchymal cells
in vitro human lung fibroblasts (NHLF)
ex vivo cultured human lung tissue slices
+ TGF-β
Transcriptomics
Proteomics
B
Transcriptome
C
Proteome
D
TF activities
tissue of origin
treatment duration
spearman correlation t-values TGF-β/control
spearman correlation logFC TGF-β/control
spearman correlation TF activities TGF-β/control
E
TF activity

Figure EV4. Cross-study Comparison of TGF-β Response in Cellular and Tissue Models.

(**A**) Overview of different studies and datasets included into the comparison analysis. The transcriptomics and proteomics data generated in this study were compared to omics data from TGF-β-treated normal human lung fibroblasts (NHLF) (Khan et al, 2024) and human lung tissue slices (Khan et al, 2021). (**B**) Spearman correlation of all transcript t-values (DEseq2 or limma, *t*-test) comparing TGF-β treatment and the DMSO or untreated control for the samples of the different studies. Note: DMSO as control was only used in the lung-related studies. (**C**) Spearman correlation of all protein log2 fold-changes comparing TGF-β treatment and the DMSO control (limma, *t* test) for the samples of the different studies. (**D**) Spearman correlation of transcription factor activities affected upon TGF-β treatment or the samples of the different studies (enzyme activity enrichment analysis using decoupleR, normalized mean method, P value < 0.1 in at least one condition). (**E**) Top hits of transcription factor activities affected upon TGF-β treatment of the samples of the different studies (enzyme activity enrichment analysis using decoupleR, normalized mean method, P value < 0.1, top 10 TFs per sample).

 © The Author(s)

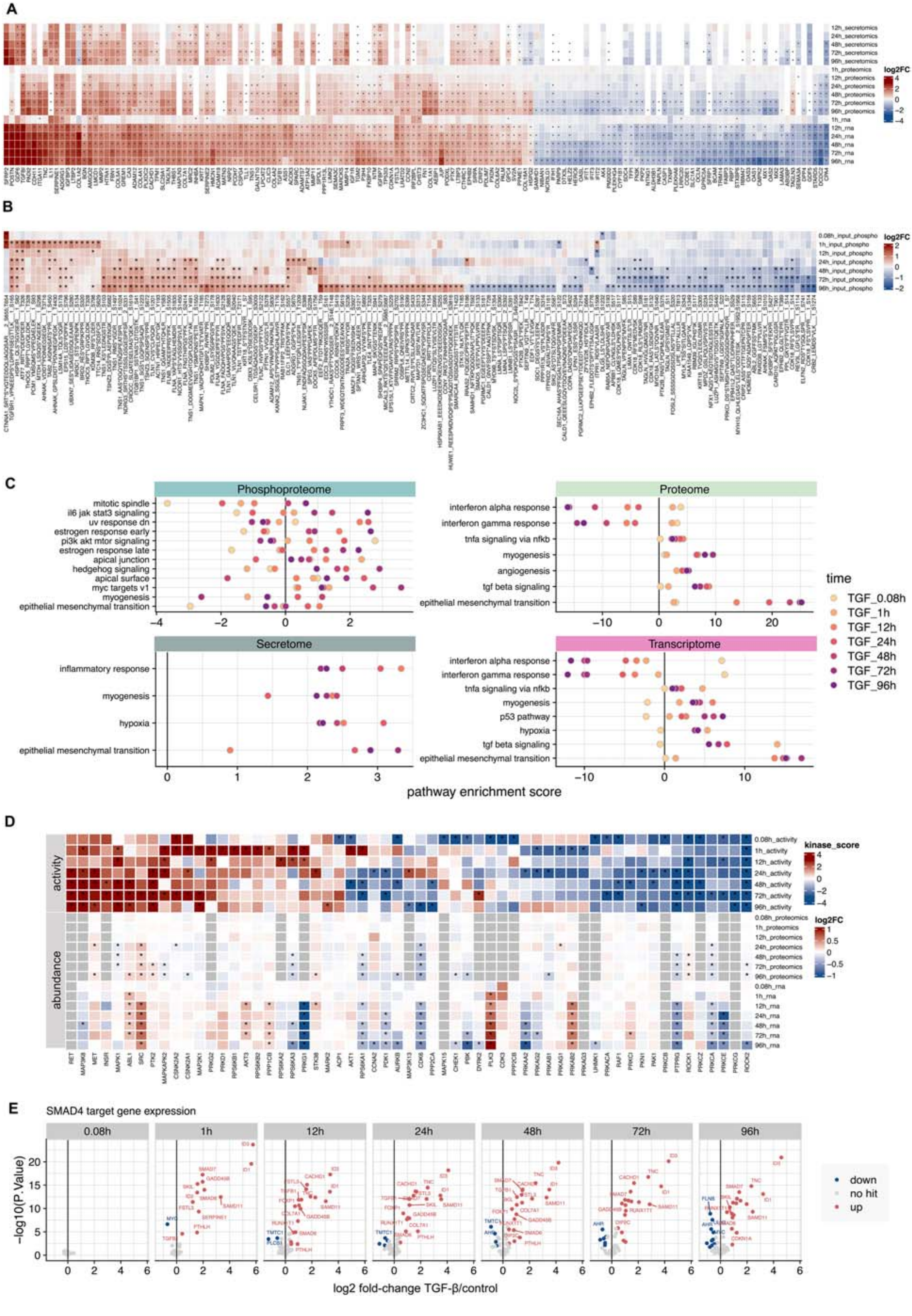
A
log2FC
B
log2FC
C
Phosphoproteome
mitotic spindle
il6 jak stat3 signaling
uv response dn
estrogen response early
pi3k akt mtor signaling
estrogen response late
apical junction
hedgehog signaling
apical surface
myc targets v1
myogenesis
epithelial mesenchymal transition
Proteome
interferon alpha response
interferon gamma response
tnfa signaling via nfkb
myogenesis
angiogenesis
tgf beta signaling
epithelial mesenchymal transition
Secretome
inflammatory response
myogenesis
hypoxia
epithelial mesenchymal transition
Transcriptome
interferon alpha response
interferon gamma response
tnfa signaling via nfkb
myogenesis
p53 pathway
hypoxia
tgf beta signaling
epithelial mesenchymal transition
pathway enrichment score
time
TGF_0.08h
TGF_1h
TGF_12h
TGF_24h
TGF_48h
TGF_72h
TGF_96h
D
activity
abundance
kinase_score
log2FC
E
SMAD4 target gene expression
0.08h
1h
12h
24h
48h
72h
96h
−log10(P.Value)
log2 fold-change TGF-β/control
down
no hit
up

Figure EV5. Temporal multi-omics analysis of TGF-β signaling dynamics.

(**A**) Heatmap of differential abundance per time point of transcripts, proteins and secreted proteins across time points. Included are genes significantly affected in at least two modalities upon TGF-β stimulation (limma, *t*-test, adjusted *P* value < 0.05, absolute log2 fold change > log2(2) for transcripts, absolute log2 fold change > log2(1.5) for proteins). The color intensity indicates the magnitude and direction of change. Exact *P* values are listed in Dataset EV1. (**B**) Heatmap displaying differential abundance per time point of the top affected phosphopeptides upon TGF-β stimulation. The color indicates the direction of change. Exact *P* values are listed in Dataset EV1. (**C**) Dot plots of top significantly enriched pathways (GSEA using decoupleR with MSIGDB reactome database, *P* value < 0.05) per time point and omics modality. Color represents the time points. (**D**) Heatmap of differential protein and transcript abundance with matched activity scores of kinases/phosphatases per time point. Kinases/phosphatases were considered if they showed significantly altered activity upon TGF-β stimulation (enzyme activity enrichment analysis using decoupleR, normalized weighted mean test, *P* value < 0.05 and absolute enrichment score > 3). Exact *P* values are listed in Dataset EV1. (**E**) Exemplary target profile of the transcription factor SMAD4 over time. Each point represents a known SMAD4 target transcript, with color indicating up- or downregulation. The *y* axis shows statistical significance ($n = 68$, limma's *t* test, BH-adjusted *P* value < 0.05 and absolute log2 fold change > log2(2)). The differential abundance signal of these known target transcripts is summarized to an activity score per time point.

© The Author(s)

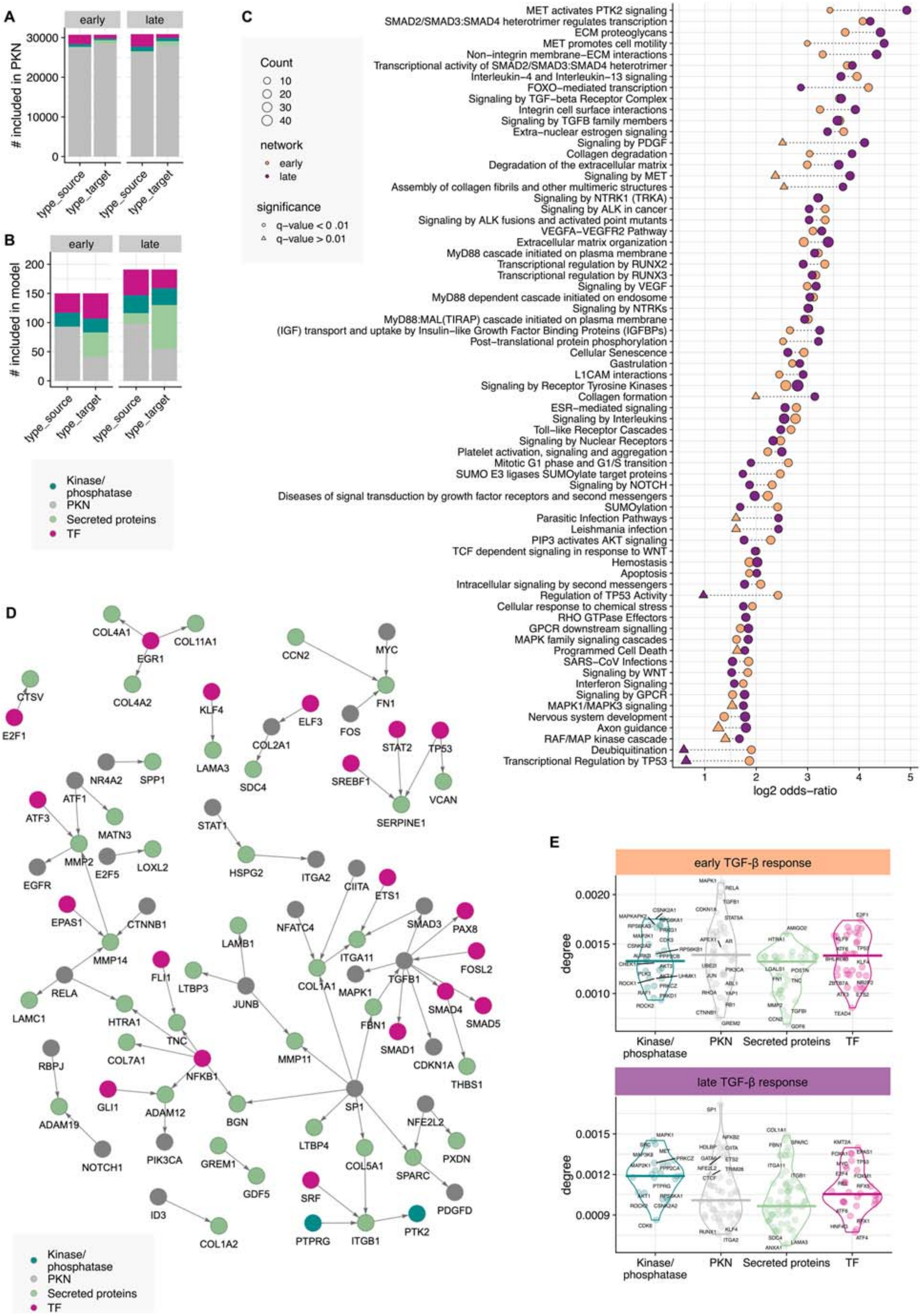
A
early
late
included in PKN
30000
20000
10000
0
type_source
type_target
B
included in model
200
150
100
50
Kinase/ phosphatase
PKN
Secreted proteins
TF
C
Count
10
20
30
40
network
early
late
significance
q-value < 0 .01
q-value > 0.01
MET activates PTK2 signaling
SMAD2/SMAD3:SMAD4 heterotrimer regulates transcription
ECM proteoglycans
MET promotes cell motility
Non-integrin membrane-ECM interactions
Transcriptional activity of SMAD2/SMAD3:SMAD4 heterotrimer
Interleukin-4 and Interleukin-13 signaling
FOXO-mediated transcription
Signaling by TGF-beta Receptor Complex
Integrin cell surface interactions
Signaling by TGFB family members
Extra-nuclear estrogen signaling
Signaling by PDGF
Collagen degradation
Degradation of the extracellular matrix
Signaling by MET
Assembly of collagen fibrils and other multimeric structures
Signaling by NTRK1 (TRKA)
Signaling by ALK in cancer
Signaling by ALK fusions and activated point mutants
VEGFA-VEGFR2 Pathway
Extracellular matrix organization
MyD88 cascade initiated on plasma membrane
Transcriptional regulation by RUNX2
Transcriptional regulation by RUNX3
Signaling by VEGF
MyD88 dependent cascade initiated on endosome
Signaling by NTRKs
MyD88:MAL(TIRAP) cascade initiated on plasma membrane
(IGF) transport and uptake by Insulin-like Growth Factor Binding Proteins (IGFBPs)
Post-translational protein phosphorylation
Cellular Senescence
Gastrulation
L1CAM interactions
Signaling by Receptor Tyrosine Kinases
Collagen formation
ESR-mediated signaling
Signaling by Interleukins
Toll-like Receptor Cascades
Signaling by Nuclear Receptors
Platelet activation, signaling and aggregation
Mitotic G1 phase and G1/S transition
SUMO E3 ligases SUMOylate target proteins
Signaling by NOTCH
Diseases of signal transduction by growth factor receptors and second messengers
SUMOylation
Parasitic Infection Pathways
Leishmania infection
PIP3 activates AKT signaling
TCF dependent signaling in response to WNT
Hemostasis
Apoptosis
Intracellular signaling by second messengers
Regulation of TP53 Activity
Cellular response to chemical stress
RHO GTPase Effectors
GPCR downstream signalling
MAPK family signaling cascades
Programmed Cell Death
SARS-CoV Infections
Signaling by WNT
Interferon Signaling
Signaling by GPCR
MAPK1/MAPK3 signaling
Nervous system development
Axon guidance
RAF/MAP kinase cascade
Deubiquitination
Transcriptional Regulation by TP53
1 2 3 4 5
log2 odds-ratio
D
COL4A1 COL11A1 EGR1 COL4A2 CTSV E2F1 KLF4 LAMA3 ELF3 COL2A1 SDC4 CCN2 MYC FN1 FOS STAT2 TP53 SREBF1 VCAN SERPINE1
NR4A2 SPP1 ATF1 ATF3 MATN3 MMP2 EGFR E2F5 LOXL2 STAT1 HSPG2 ITGA2 CIITA ETS1
EPAS1 CTNNB1 MMP14 FLI1 LTBP3 LAMB1 JUNB NFATC4 COL1A1 ITGA11 MAPK1 SMAD3 PAX8 TGFB1 FOSL2
RELA LAMC1 HTRA1 TNC COL7A1 NFKB1 FBN1 SMAD4 SMAD5 SMAD1 CDKN1A THBS1 MMP11
RBPJ ADAM19 GLI1 ADAM12 BGN SP1 NFE2L2 NOTCH1 PIK3CA LTBP4 COL5A1 PXDN GREM1 SPARC GDF5 PDGFD
ID3 COL1A2 SRF PTPRG ITGB1 PTK2
Kinase/ phosphatase
PKN
Secreted proteins
TF
E
early TGF-β response
late TGF-β response
degree
0.0020
0.0015
0.0010
0.0012
0.0009
Kinase/ phosphatase
PKN
Secreted proteins
TF

Figure EV6. Network structure and analysis of early and late TGF-β response models.

(**A**) Overview of node types in the prior knowledge network. All modalities are reflected as protein-protein interaction source and target for the early and late TGF-β response model. (**B**) Overview of node types in the obtained solution network models for early and late TGF-β response. The node type for protein-protein interaction source and target nodes reflect the chosen hierarchy. (**C**) Significant pathways in early and late TGF-β response network model (pathway overrepresentation analysis, unpaired two-sided Wilcoxon test, of network nodes using the Reactome pathway database, P value < 0.05). Point color indicates the network for which the enrichment has been calculated. Point size shows the number of nodes in the network mapped to the pathway. (**D**) Reactome TGF-β signaling pathway in network models shows links between different node types. Node color indicates node type. (**E**) Node centrality in the early and late network model per node type (n in the order of the plot, early: 21, 36, 40, 33; late: 21, 50, 66, 27).

 © The Author(s)

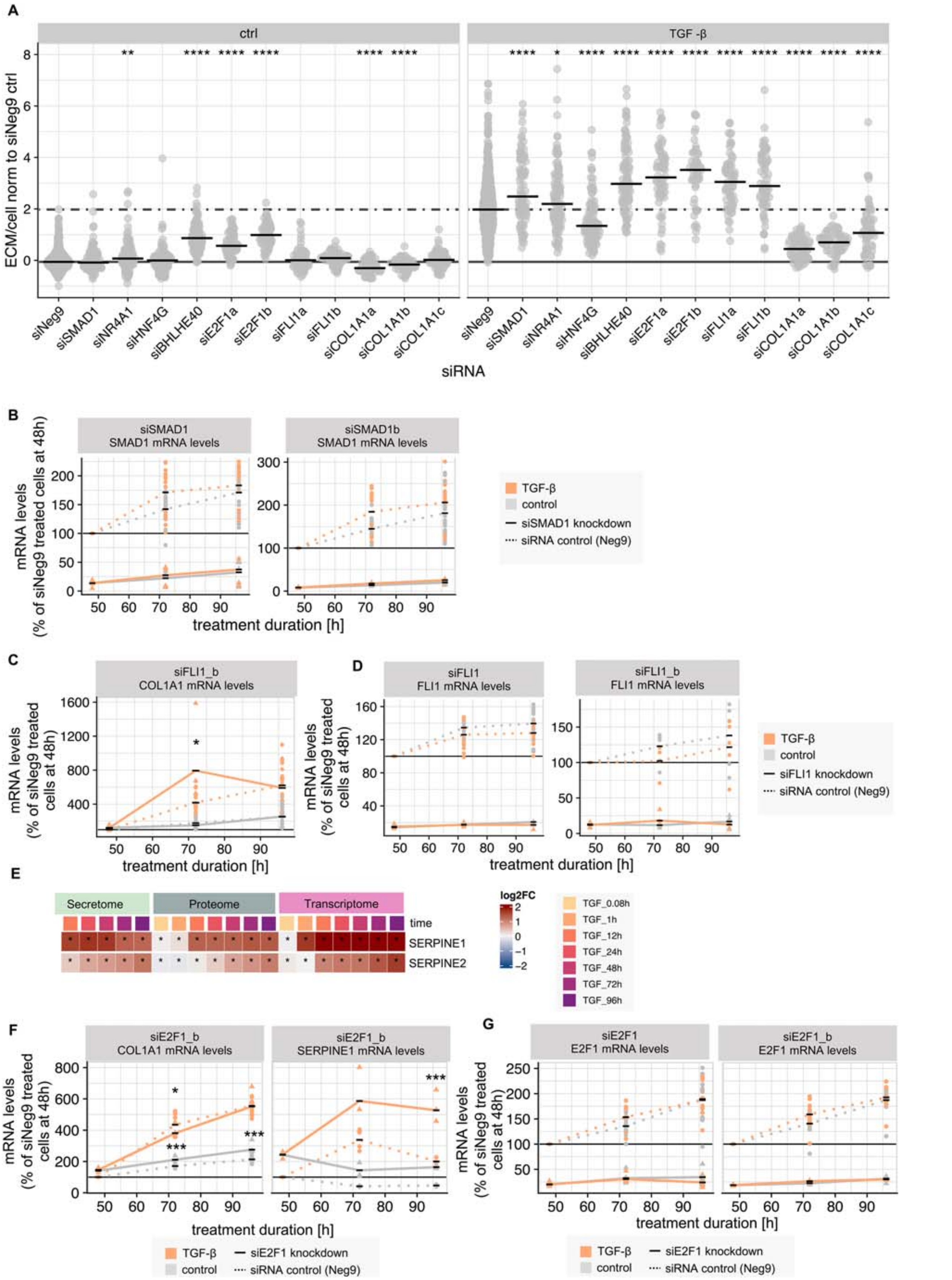
A
ctrl
TGF -β
ECM/cell norm to siNeg9 ctrl
siRNA
siNeg9
siSMAD1
siNR4A1
siHNF4G
siBHLHE40
siE2F1a
siE2F1b
siFLI1a
siFLI1b
siCOL1A1a
siCOL1A1b
siCOL1A1c
B
siSMAD1
SMAD1 mRNA levels
siSMAD1b
SMAD1 mRNA levels
mRNA levels
(% of siNeg9 treated cells at 48h)
treatment duration [h]
TGF-β
control
siSMAD1 knockdown
siRNA control (Neg9)
C
siFLI1_b
COL1A1 mRNA levels
D
siFLI1
FLI1 mRNA levels
siFLI1_b
FLI1 mRNA levels
siFLI1 knockdown
E
Secretome
Proteome
Transcriptome
time
SERPINE1
SERPINE2
log2FC
TGF_0.08h
TGF_1h
TGF_12h
TGF_24h
TGF_48h
TGF_72h
TGF_96h
F
siE2F1_b
COL1A1 mRNA levels
siE2F1_b
SERPINE1 mRNA levels
G
siE2F1
E2F1 mRNA levels
siE2F1_b
E2F1 mRNA levels
siE2F1 knockdown

◀ **Figure EV7. Functional validation of key transcription factors regulating ECM deposition and fibrotic gene expression.**

(**A**) Fluorescence intensity of ECM for both untreated (ctrl) and TGF-β stimulated conditions for all performed knockdowns (96 h knockdown followed by 48 h +/- TGF-β treatment). Intensities of each knockdown were compared to the siNeg9 control condition (+/- TGF-β treatment) using a two-sided unpaired *t* test ($n = 2$–3 biological replicates, each with ~ 36 images, * corresponds to *P* value < 0.05, ** corresponds to *P* value < 0.01, *** corresponds to *P* value < 0.001, exact *P* values as they appear in the plot: 0.46, 0.0073, 0.67, 2.2e-16, 2.2e-16, 2.2e1-6, 0.32, 0.05, 2.2e-6, 0.42, 8.7e-6, 0.023, 1e-6, 1.7e-13, 2e-10, 4.1e-11, 2.3e-10, 8.5e-9, 2.2e-16, 2.2e-16, 1.2e-9). For panels B-D and F, color indicates TGF-β stimulation (orange) vs control (gray). Line type distinguishes between siNeg9 control (solid) and target gene knockdown (dashed). (**B**) RT-qPCR data showing SMAD1 knockdown efficiency for various mRNAs across different time points ($n = 3$–5 biological replicates). (**C**) RT-qPCR results demonstrating the effect of FLI1 knockdown (using a second set of siRNA) on its potential downstream target COL1A1 at different time points. Significance has been tested per time point and treatment condition using a two-sided unpaired *t* test ($n = 4$ biological replicates, * corresponds to *P* value < 0.05, ** corresponds to *P* value < 0.01, *** corresponds to *P* value < 0.001, exact *P* values as they appear in the plot: 0.0128). (**D**) RT-qPCR data confirming FLI1 knockdown efficiency for multiple mRNAs at various time points ($n = 3$–4 biological replicates). (**E**) Differential expression analysis results for SERPINE1 and SERPINE2 in the secretomics, proteomics, and transcriptomics data per time point. Significance is indicated by one star (limma *t* test, adjusted *P* value < 0.05, absolute log2 fold change > log2(1.5) or absolute log2 fold change > log2(2) for transcriptomics data, Exact *P* values are listed in Dataset EV1). (**F**) RT-qPCR data to confirm E2F1 knockdown effect on its potential downstream targets COL1A1 and SERPINE1 + /-TGF-β stimulation at different time points using a second set of siRNA.Significance has been tested per time point and treatment condition using a two-sided unpaired *t* test ($n = 4$ biological replicates, * corresponds to *P* value < 0.05, ** corresponds to *P* value < 0.01, *** corresponds to *P* value < 0.001, exact *P* values as they appear in the plot: 0.03, 0.000239, 0.00000151, 0.000298, 0.00676, 0.000000510). (**G**) RT-qPCR data to confirm E2F1 knockdown efficiency for different mRNAs (columns) +/-TGF-β stimulation at different time points ($n = 3$–4 biological replicates).

© The Author(s)
